# People use fast and flat simulation to reason about new games

**DOI:** 10.1038/s41586-026-10722-1

**Published:** 2026-07-15

**Authors:** Katherine M. Collins, Cedegao E. Zhang, Lionel Wong, Mauricio Barba da Costa, Graham Todd, Adrian Weller, Samuel J. Cheyette, Thomas L. Griffiths, Joshua B. Tenenbaum

**Affiliations:** 1 Massachusetts Institute of Technology, Cambridge, MA USA; 2 Princeton University, Princeton, NJ USA; 3 University of Cambridge, Cambridge, UK; 4 Stanford University, Stanford, CA USA; 5 New York University, New York City, NY USA; 6 The Alan Turing Institute, London, UK

**Keywords:** Human behaviour, Computer science

## Abstract

Games have long been a microcosm for studying planning and reasoning in both natural and artificial intelligence, often focusing on expert-level or even super-human play^1–6^. But real life also pushes human intelligence along a different frontier, requiring people to flexibly navigate decision-making problems that they have never thought about before. Here we use novice gameplay to study how people reason about new problem settings. Through a series of large-scale behavioural studies with over 1,000 participants and 121 two-player strategic board games (almost all novel to our participants), we show that people are systematic and adaptively rational in how they play a game for the first time or evaluate a game (for example, how fair or how fun it is likely to be) before they have played it even once. We explain these capacities via a computational cognitive model that we call the ‘Intuitive Gamer’: a model based on mechanisms of fast and flat (depth-limited) goal-directed probabilistic simulation. Our work offers insights into how people rapidly evaluate, act and make suggestions when encountering novel problems, and could inform the design of more flexible and human-like artificial intelligence systems that can determine not just how to solve new tasks but also whether a task is worth thinking about at all.

## Abstract (web summary)

Large-scale behavioural studies show that people approach a new game systematically and adaptively rationally. A cognitive model called the ‘Intuitive Gamer’ offers insights into this process and may inform the design of more flexible AI systems.

## Main

Games occupy a special space in psychology and and computer science^1–4,6–12^ for good reason: games are systems of rules and reward that hold a mirror to the structures, patterns and challenges that reality confronts us with, across familiar and merely possible experiences. Mancala lets us sow and capture abstract resources. Chess lets us strategize over bloodless battlefields. Go lets us encircle and claim territories with silent stone troops. A good game lets us thrill to uncertainties and losses that we might shy from in real life.

Substantial work in cognitive science focuses on the nature and development of expertise, using games as a testbed^3,6,13^. Human experts, correspondingly, have long served as goalposts in the quest to build intelligent machines, with a particular focus on emulating and ultimately beating humans at their own games, using more game-playing data and raw computational horsepower than any human expert could acquire in their lifetime^2,4,14,15^.

But people are not experts at most of the problems they encounter throughout their lives, or most systems they are thrown into before they need to decide on an action. Human cognition is flexible enough to consider many potential problems and many potential systems of rule and reward. Thinking about whether a system of rules and reward makes sense, whether to participate, or what actions to take first—across a wide range of novel situations—is arguably at least as important for everyday human cognition (if far less studied) than the cognitive processes that lead a small number of humans to become expert players in any one game.

**Fig. 1:**
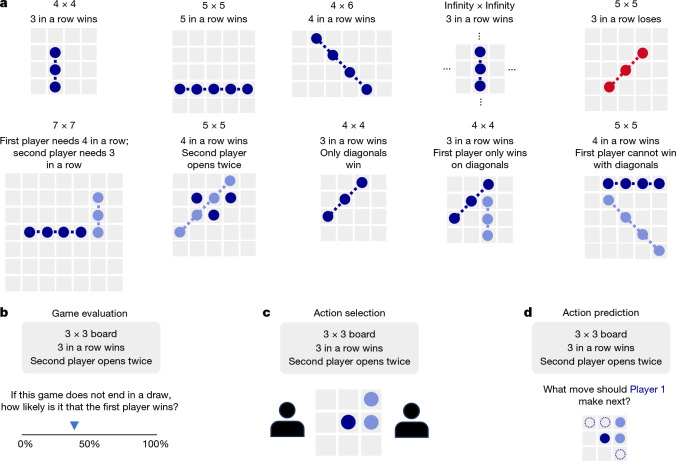
Our novel game dataset and suite of game tasks. **a**, Ten example games from our 121 game dataset. Games vary in board sizes and rules, such as what it takes to win and how many pieces any player can place on their opening move. **b**–**d**, We assess people’s reasoning about novel games through three behavioural studies designed to test: how people reason about games before they even play a single game (**b**); how people decide what actions to make in their first instance of play (**c**); and how people predict others should play when watching them play (**d**).

Here we study the abilities of novice game reasoners across a range of over 100 novel games from a subclass of strategic grid-based board games that are not entirely unlike games they have probably seen before (for example, Tic-Tac-Toe or Connect-4), but vary in their rules and underlying dynamics (see examples in Fig. 1a and Extended Data Table 1). In a series of large-scale behavioural studies, each with hundreds of participants, we assess people’s thinking about novel games in three reasoning settings (Fig. 1b–d): game evaluation (determining how rewarding a game is likely to be before playing); action selection (choosing moves when playing for the first time, with another first-time player); and action prediction (judging the likelihood of moves other first-time players might make without having played directly themselves). Our setting is different from much previous work on games, which usually focuses on one or a small set of games and many rounds of play in that game; here, we consider many games and little (or even no) experience.

**Fig. 2:**
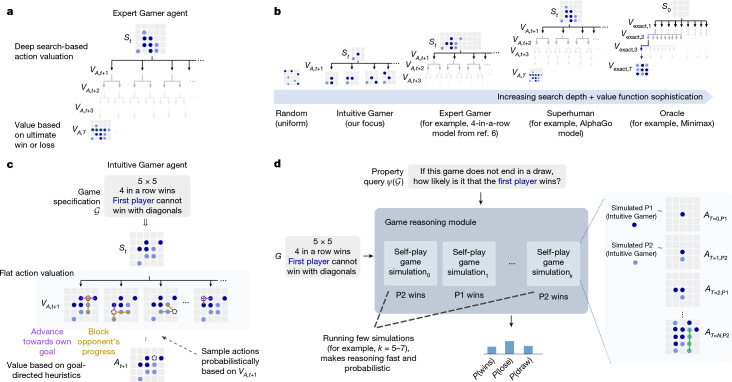
The Intuitive Gamer model compared with previous models of game reasoning. **a**, Previous work modelling expert gameplay often involves deep tree search to determine what move to make, given a board state *S*_*t*_ (refs. ^4,6^). It is unlikely that novice human reasoners conduct such computationally expensive search and state evaluation before deciding whether to engage with the problem at all. **b**, We consider what novice reasoners might be doing instead. Gameplay agents can differ in the amount of compute and expertise brought to bear to reason about any game (differing in search depth and value sophistication). Our proposal is that people reasoning about problems with which they have no experience sit at the lower end of this spectrum, but not the lowest. **c**, The Intuitive Gamer conducts depth-limited (‘flat’) search with game-general abstract goal-directed value functions that have yet to encode game-specific features. The Intuitive Gamer gameplay agent conducts no more than a single step of lookahead when deciding what action (*A*_*t*+1_) to take from a given board state (*S*_*t*_) at a given move turn *t*. The Intuitive Gamer is goal-directed, assessing whether any action would advance the player’s own goal (purple) and how much it might block the progress of the opponent’s goal (yellow). The final action is selected probabilistically by sampling from a softmax distribution over the estimated values per action. **d**, To reason about any new game query *ψ* for a given game description *G*, we posit that people conduct only a few (*k*) self-play simulations between the gameplay agent (as depicted in **c**) to answer the query, which could be run to termination or probabilistically stop early. In summary, the Intuitive Gamer model is fast (low *k*), flat (low search depth), goal-directed (in the value function) and probabilistic (in action selection), and involves mental simulations of gameplay. See Methods for a detailed formalism of the model.

Our core contribution is a computational account of how people reason about these novel problems. Expert models of gameplay typically involve deep tree search with potentially thousands of evaluations of possible states (Fig. 2a). It seems unlikely that a novice reasoner is engaging in such intensive search and evaluation before they have extensive—or any—experience. But neither are people likely to be completely unsystematic. We hypothesize that novice thinking occupies a valuable intermediate point between these extremes (Fig. 2b): people are non-random in assessing new problems and run computations analogous to those that underlie state-of-the-art artificial intelligence (AI) game systems and cognitive models of expert human gameplay^4,6,14^—but scaled down to a level that is more realistic for everyday thought.

We instantiate and test this hypothesis in an Intuitive Gamer model that makes judgements and decisions by running mental simulations of gameplay that are (1) fast, (2) flat, (3) goal-directed and (4) probabilistic. The Intuitive Gamer model accounts very well—and significantly better than alternative models—for how people evaluate these games before play. Features computed from the same model can capture people’s judgements about the game’s objective value, as well as more subjective evaluations such as a game’s expected ‘funness’. Action choices simulated by this same model generally capture how novices actually play in games and how they make predictions about unfolding games they have never played themselves.

## The Intuitive Gamer model

The capacity to evaluate properties of a new game and decide whether the game is worth engaging with might intuitively feel like it comes before developing a policy for actually playing the game. However, our intuition as modellers is the opposite: game properties such as expected values depend on both the rule specifications of a game and the policies adopted by the players. We hypothesize that this could be true for novice human game reasoners—before taking a single action in the game—if their policies are extremely simple to construct, fast to execute and support probabilistic sampling of reasonable action choices. If so, a policy can be queried just a small number of times to simulate gameplay, from which the game reasoner can draw reasonable inferences about the game’s properties using these simulated traces.

We instantiate this hypothesis with our Intuitive Gamer model. The model consists of two modules: a ‘flat’ game-playing agent, which runs an extremely depth-limited search to select actions probabilistically on the basis of abstract yet locally evaluable ‘goal-directed’ heuristic functions (Fig. 2c); and a ‘fast’ game reasoner that makes probabilistic inferences about game propositions and their expected values using a small number of simulated games (Fig. 2d).

### Player module

A long tradition of modelling human behaviour in games has used some combination of game-state evaluation functions and search over the decision tree of game states^16,17^, as depicted in Fig. 2b, to construct a policy. Our approach broadly falls into this tradition. However, existing accounts typically assume highly tuned or expert-designed value functions and deep simulation^2,6,14,18^, which by definition cannot be expected of a novice. Rather, we propose a model that combines a general-purpose abstract value function—which could be easily constructed from understanding the rules of the game—with depth-limited search. Instead of simulating the downstream effects of a move via extensive forward search, the Intuitive Gamer player module uses only a single step of ‘look-ahead’ and selects an action probabilistically by evaluating the resulting board states according to a small collection of general heuristics (Fig. 2c). These heuristics are built on the assumption that players understand and pursue their goals (as defined by the game rules) and attempt to prevent their opponents from doing the same, subject to constraints on computational resources^19,20^. The Intuitive Gamer player module then is a more general, compute-bounded version of prior game-based agents (Methods).

### Reasoning module

Whereas the Intuitive Gamer player model captures action choice during a game, the Intuitive Gamer reasoning module captures judgements about game properties by nesting the player module within a sample-based probabilistic inference procedure (Fig. 2d). For any game, the reasoning module infers answers to a query (for example, what is the likelihood that the game will end in a win, loss or draw for a given player?) by simulating *k* game playouts and iteratively calling the player module at each turn of self-play. Each simulation ends when the game reaches a win or draw state, or may terminate early with some probability. Computations over the simulated gameplay traces then inform the resulting distribution over the query values (for example, game outcomes). We assume simulated games are independent, though this could be relaxed in the future (see ‘Discussion’). In keeping with our focus on fast, resource-limited reasoning, only a small number of game simulations are used (which we estimated empirically, see results and Methods) in line with previous evidence that people use a relatively small number of simulations to form beliefs and make decisions^21–26^.

### Alternative models

Our Intuitive Gamer framework naturally extends to model players with more or less competence and motivation. We can think of the Intuitive Gamer as operating under the same mechanisms as more expert models, but scaled down (offering, therefore, a path to scale back up). We can vary parameters in our framework (value function and search depth) to instantiate a version of the kind of cognitive models that have been successful at capturing human experts in a single ‘four-in-a-row’ game^6^. We refer to this variant as the ‘Expert Gamer’, which differs from the Intuitive Gamer by conducting a deeper search (approximately depth-5) and using a more sophisticated value function (Methods). On the other end of the spectrum, we consider unmotivated players who select actions uniformly at random (Fig. 2b), which we refer to as the ‘Random Gamer’. We also compare reasoning judgements to Monte Carlo tree search (MCTS)^4,27,28^, an alternative tree-search approach that is even more computationally intensive and popular in AI systems for strong general game playing (see Methods for contrast with our Intuitive Gamer and other cognitive models). Finally, we compare against models that do not involve explicit structured game simulation, operating either over pre-computed linguistic features from the game description alone (Methods and Supplementary Information section 5) or more flexible linguistic computable models, for example, language models (Supplementary Information section  5).

### Resource-rational reasoning

The Intuitive Gamer occupies an interesting point on the Pareto frontier of compute efficiency and game reasoning sophistication. On the one hand, the Intuitive Gamer is orders of magnitude faster than more sophisticated game reasoners (MCTS and the Expert Gamer) in wall clock time and number of board states evaluated (Extended Data Table 2a and Supplementary Information). The Intuitive Gamer is approximately 700× faster than the Expert Gamer in wall clock time, with approximately 500× fewer board evaluations, and is nearly 40,000× faster than MCTS, with almost 10,000× fewer node evaluations, as computed under self-play game simulations for the respective models. On the other hand, although not as close to game-theoretic optimal play as the Expert Gamer or MCTS, game evaluations under the Intuitive Gamer are still well-correlated with game-theoretic optimal analyses and far more aligned with game-theoretic optimal play than random play (Extended Data Table 2b and Methods). The Intuitive Gamer’s mechanisms of fast, flat goal-directed probabilistic simulation thus constitute a highly efficient and resource-rational approach to game reasoning, which we hypothesize people could plausibly and productively engage when encountering new games.

### Games to study novice game reasoning

To explore how people reason about new games within the bounds of a behavioural study, we need a broad collection of games that are novel without requiring lengthy demonstrations or explanations. To that end, we implement a range of two-player, grid-based strategy games derived from classic *M*–*N*–*K* games (examples include tic-tac-toe and gomoku, in which players take turns placing pieces on an *M* × *N* grid trying to make *K* pieces in a row—a subgenre of games frequently studied in previous work^6,29,30^). Our set of 121 distinct games covers a variety of environment specifications (for example, the size and shape of the board), transition dynamics (for example, the number of moves a player makes on their turn) and win conditions (for example, whether completing a line results in a win or a loss). In addition, games vary in their duration and balance under optimal play (Methods). In each case, however, the core mechanic of the game (that is, placing a piece into an empty grid cell) remains both consistent and familiar. See examples in Fig. 1a and Extended Data Table 1.

## Reasoning about games before any play

We first assess how people evaluate a game (Fig. 1b), from ‘just thinking’ about the game alone—before any play. We consider two kinds of game evaluation: how people assess the expected outcomes of a game and a more subjective assessment of whether the game is likely to be engaging to play (its funness).

### Is the game likely to be fair?

We recruited 238 participants to evaluate the expected outcomes of the game in a ‘zero-shot, zero-experience’ experiment. Participants ‘just thought’ about the games, estimating both the likelihood of a draw and the likelihood that the first player would win if the game did not end in a draw for games solely on the basis of a natural language description of the rules and a depiction of a blank board. These estimates define an expected payoff of the game from the perspective of the first player (Methods), which in turn encodes an intuitive notion of fairness (that is, whether a game is biased towards a particular player).

**Fig. 3:**
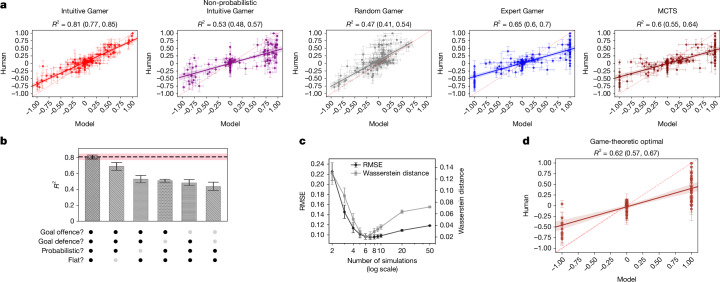
Evaluating games without ever playing. A total of 238 participants judged the expected payoff of games drawn from our 121-game suite. **a**, Expected payoff predicted by humans and by alternative models for each game. Each point represents the payoff for one of the *n* = 121 game stimuli. Error bars show 95% CIs around the mean human estimate and the mean model prediction from *k* = 6 simulations sampled from 20 simulated participants. **b**, Variance explained (*R*^2^) by the full Intuitive Gamer and lesioned variants. Removing flatness, probabilistic simulation or goal-directedness reduces the fit to human payoff judgements. The dashed line indicates the mean and 95% CI split-half *R*^2^ on the human-predicted payoffs, indicating that the full Intuitive Gamer model captures essentially all of the explainable variance that is not due to noise. **c**, Fit to the variance of human payoff judgements as a function of the number of simulations *k*. A small number of simulations (*k* ≈ 5–7; we use *k* = 6) best captures the variability in participants’ judgements. Fit is measured by the root mean squared error (RMSE) between human and model variance by game and by Wasserstein distance between their variance distributions across games. Full scatterplots are shown in Extended Data Fig. 1, and details of our sample complexity analysis are in the Methods. **d**, Expected payoff predicted by humans and by game-theoretic optimal analysis for the 78 games (out of 121 games) for which an optimal payoff could be computed. Error bars depict 95% CIs around the bootstrapped mean human prediction per game.

We assessed how each characteristic of the Intuitive Gamer model (fastness, flatness, goal-directedness and probabilistic simulation) contributes to people’s payoff predictions by comparing with alternative models that increase or decrease the sophistication of each component (Fig. 3a). The Intuitive Gamer model correlates well with human estimates (*R*^2^ = 0.81 (95% confidence interval (CI), 0.77, 0.85)), matching the total explainable variance from the human data as estimated by split-half correlations (*R*^2^ = 0.82 (95% CI, 0.77, 0.86)). The Intuitive Gamer captures human judgements significantly better than alternative models that vary in sophistication. It outperforms the Random Gamer model (*R*^2^ = 0.47 (95% CI, 0.41, 0.54)), Expert Gamer model (*R*^2^ = 0.65 (95% CI, 0.60, 0.70)), MCTS baseline (*R*^2^ = 0.60 (95% CI, 0.55, 0.64)) and a variant of the Intuitive Gamer that is not probabilistic (*R*^2^ = 0.53 (95% CI, 0.48, 0.57)).

**Extended Data Fig. 1:**
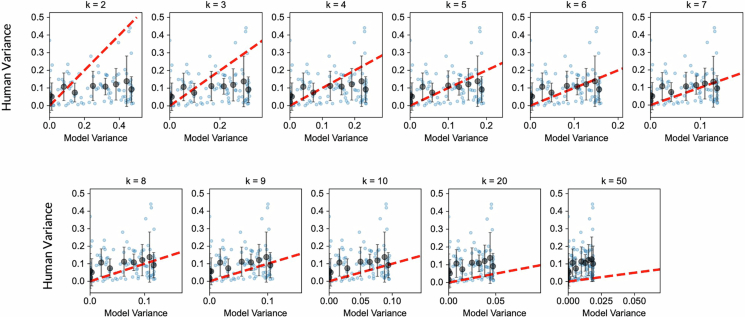
Sample complexity analysis, based on the variance of human and model payoff predictions with varied compute budget (number of simulations, *k*) for the Intuitive Gamer model). The variance over each approximately 20 human participant payoff judgments per game and compare against the average variance under simulated sets of *N* = 20 simulated participants each drawing *k* simulations from the model. Model variance is binned into quintiles along the horizontal axis and the average human variance is computed for points in that bin (the black dot overlaid on the scatterplot). Error bars are standard deviation of the variance for points in that bin.

**Extended Data Fig. 2:**
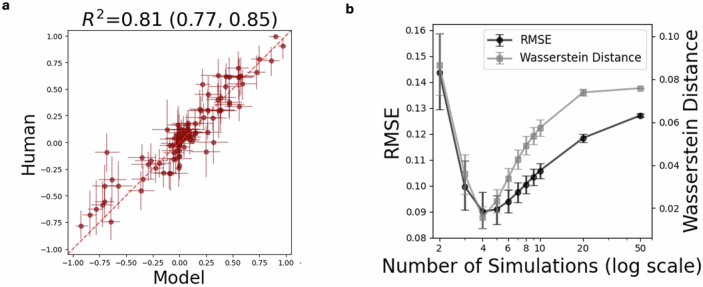
Partial game simulations in the Intuitive Gamer reasoning module. All main results are reported under the assumption that the game reasoning module runs simulations to the end. While most games do not involve a large number of moves, under the Intuitive Gamer player module, for the game to end—it is possible that people are engaging even cheaper partial simulations when evaluating novel games for the first time. **a**, Exploratory analyses into running the Intuitive Gamer reasoning module under a probabilistic stopping rule equivalently captures the majority of the explainable variance in human payoff judgments as running the Intuitive Gamer reasoning module under full simulations (see Fig. 3a). For direct comparison with the full simulation model, *k* = 6 partial simulations were run to estimate game payoff. The partial simulations were computed as follows. For each simulated match of a game, a stopping time was sampled from a uniform distribution over board size and terminated early if the game did not end before that time. If the game terminated before a winner or draw was called, the game counted as a draw. **b**, To assess the impact of compute budget in the number of simulations used to estimate payoff, the variance of payoff under different number of partial simulations *k* was again compared with the variance in people’s payoff evaluations (as in Fig. 3c and Extended Data Fig. 1). The best fitting number of samples (*k*) is similarly greater than one and less than ten when using partial simulations, and perhaps even a bit less (e.g., *k* = 4) compared to using full game simulations (see Fig. 3c).

To more directly probe each component of the Intuitive Gamer model, we conducted a series of experiments in which we varied key model components singly and in combination (see Supplementary Information section 1 for a complete summary). The base model can be parameterized by a series of binary choices: whether to be goal-directed in value assessments; whether to be probabilistic; and whether to be flat. Modifying any of these factors relative to the full model impairs the fit to human payoff judgements (Fig. 3b). ‘Fastness’ can be further varied by modulating the number of simulations the Intuitive Gamer reasoning module takes when assessing payoff. A small number of simulations (*k* more than one, but less than ten) best captures the variance in human prediction (Fig. 3c and Extended Data Fig. 1; see complexity analysis details in Methods). These comparisons suggest that human payoff judgements of novel games are well-modelled by efficient and compute-limited simulation rather than purely random exploration of game states or deeper search and computationally intensive simulation. For simplicity, this estimate assumes that all simulations are independent and run to the end of the game; allowing simulations to probabilistically terminate early (Methods) yields similar fits in the expected payoff evaluations and number of mental simulations per participant (Extended Data Fig. 2).

We were able to estimate the optimal game-theoretic payoff for many (78 of 121; Methods) of the games, allowing us to compare people’s subjective judgements with a fully objective game evaluation. Human judgements are reasonable relative to the game-theoretic optimal (*R*^2^ = 0.62 (95% CI, 0.57, 0.67)). Although human judgements generally track the direction of the game-theoretic optimal (for example, estimating a payoff greater than zero when the first player should definitely win), the Intuitive Gamer provides a superior qualitative and quantitative fit to human judgements.

### Is the game likely to be fun?

**Fig. 4:**
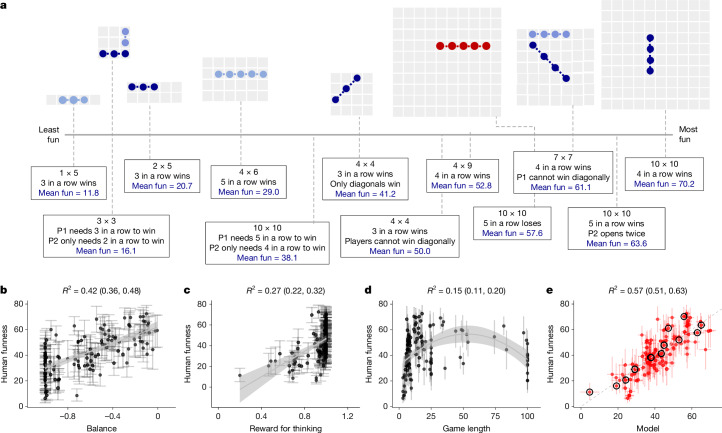
Evaluating whether games are likely to be fun before ever playing them. A total of 246 participants judged whether they thought the games would be fun. **a**, Representative games rated by people as more or less fun, with position on the spectrum arranged approximately according to the mean of participants’ funness ratings. **b**–**d**, These funness ratings are related to several game features that can be read out under Intuitive Gamer model simulations: game balance (**b**); predicted advantage over a random agent given the Intuitive Gamer model’s gameplay (reward for thinking; **c**); and expected game length, as fit with a quadratic term to account for a nonlinear relationship to funness (**d**). **e**, A regression model with all features predicted under the Intuitive Gamer model (balance, reward for thinking, and length) captures much of the explainable variance in the human funness ratings (*R*^2^ = 0.57 (95% CI, 0.51, 0.63) compared with split-half *R*^2^ = 0.60 (95% CI, 0.51, 0.68)). Error bars depict 95% CIs around the human mean funness per game; error bars for the model depict 95% CI over the predicted mean for each of the fit models (models are fit to each bootstrap subsample). Black circles show games from **a**, highlighting that funness judgements under the Intuitive Gamer generally align with the ordering of funness of games judged by people. A full listing of the games and their average human funness scores are in Supplementary Information section 8.

**Extended Data Fig. 3:**
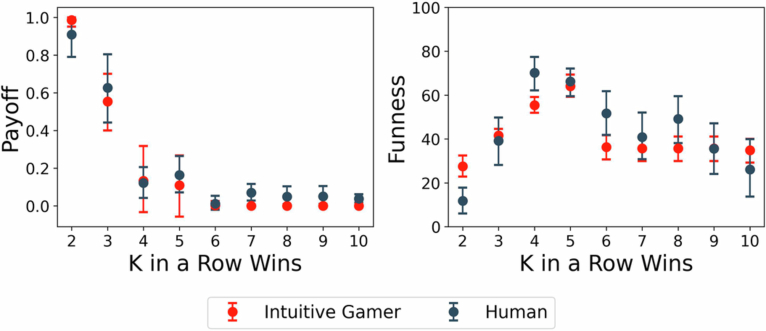
Systematic variation in human and model predictions with change in game rules. Human and model judgments follow similar qualitative trends in **a**, payoff and **b**, funness predictions as *K* varies for a 10 × 10 board. Human and model average predictions are shown. Error bars depict 95% CIs over the mean (1000 bootstraps). Funness model error bars (**b**) are computed over repeated model fits to 50% splits of the games, holding out the subset of 10 × 10 games of varied *K*(∈ {2, . . . , 10}) from each train split. These data reveal systematic and interpretable patterns in the human data (e.g., estimated payoff plateauing towards zero with increased *K*; funness peaking at a intermediate values of *K*). In particular, when *K* is too small (3 or less) the game is unbalanced, as the first player can easily win and the Intuitive Gamer simulates this easily. For too large values of *K*, the game is hard for either player to win and there is little reward for thinking; the Intuitive Gamer also simulates this.

We ran a parallel ‘zero-shot zero-experience’ study as described above on a new group of 246 participants who were asked to make a subjective estimate of how fun a game is likely to be, before ever playing, to test whether the Intuitive Gamer’s reasoning process can also account for how people make more subjective evaluations about new problems. Games varied widely in their judged funness (Fig. 4a and Supplementary Information section 8), and funness judgements generally varied more across individuals than judgements of expected payoff, which is reasonable given that funness is both more vague and a more subjective evaluation. Games that were judged most fun tended to have larger boards (often 10 × 10) and win conditions connecting a moderate number of pieces in a row (often 4 or 5), but there is no simple relation between these features and funness: many 10 × 10 games were judged below average (Extended Data Fig. 3b), and the single most fun game was a 5 × 5 board with a misère rule, where 3 in a row loses.

We hypothesized that several features of a game that can be read off easily from our model’s fast and flat probabilistic simulations—how balanced a game is, how much a game rewards thinking, and how long a game is—could quantitatively explain much (or even most) of the variance in people’s funness judgements. Our measure of balance captures the notion that people prefer games they expect will be decisive (unlikely to end in a draw), where both players have an equal chance to win. Our thinking reward captures the relative advantage of playing strategically (‘thinking’) versus moving haphazardly (‘without thinking’). The effect of game length is modelled as an inverted U-shape of expected number of moves to a draw or win, reflecting the intuition that longer and more involved games tend to be more fun, unless they extend unnecessarily. (For details of how these game features are computed, see Methods.)

**Extended Data Fig. 4:**
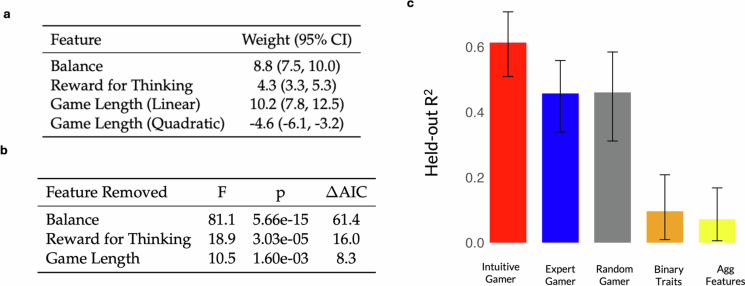
Funness model feature importances and generalization test comparing funness under alternate Gamer agents and non-simulation based funness models. **a**, Funness model feature weights reveal that games that are more balanced are generally considered more fun, as are games that are more rewarding to think about (though they contribute less per unit to the funness than balance). Game length has an inverted-U shaped relationship to fun (as evidenced by the positive linear weight and negative quadratic weight; games that are too short or too long are less fun). Weights of the features over 1000 models fits to bootstrapped mean human funness predictions, over all games. Coefficients are over normalized features (see Methods). **b**, Ablating each linear feature in the primary funness model reveals that all features matter. Higher *F*-statistic means that ablating the feature explains less of the variance in the human data as the full model. ΔAIC is AIC_ablated_ - AIC_full_ (a higher value indicates that ablating the feature impairs fit to human data, relative to the full model). **c**, Simulations under the Intuitive Gamer model better capture human judgments than simulations under models varying the computational sophistication (Random and Expert Gamer) in a generalization test, where the parameters of each model are fit on 50% of the games and tested on the other 50% of games. Funness models based on explicit simulation better capture human judgments than models based only on non-simulation based linguistic features (e.g., binary game traits—whether the game has asymmetric win conditions, non-square boards, etc) and aggregate features based binary traits (counts of how many are “on” to capture “novelty” relative to Tic-Tac-Toe; and board size, encoded as number of rows  × number of columns). Error bars depict 95% CIs of the held-out *R*^2^ over 500 bootstrapped train/test splits.

Each of these three features computed under the Intuitive Gamer model captures significant variance in human funness judgements (Fig. 4b–d and Extended Data Fig. 4a,b). A simple regression model that combines these features (including a quadratic game length term) attains *R*^2^ = 0.57 (95% CI: 0.51, 0.63) (Fig. 4e), which approaches the total variance explainable in the human data based on bootstrapped split-half *R*^2^ from participants over all games (*R*^2^ = 0.60 (95% CI: 0.51, 0.68)). This model also quantitatively explains the qualitative trends we observed in funness judgements, capturing the nonlinear relation between funness, board size and the number *K* in a row to win (Extended Data Fig. 3).

Finally, a generalization test (Methods) showed that these fits are not sensitive to the choice of games, and that game features estimated from Intuitive Gamer simulations predict funness judgements better than those from the Random or Expert Gamer models, using more or fewer compute resources (depth of thinking), or models that relied exclusively on surface-level game features (Extended Data Fig. 4c and Supplementary Information section 5.7).

Taken together, these two experiments in which participants ‘just think’ to rate both objective measures of a game (for example, its payoff or fairness) and more subjective evaluations (for example, its enjoyability) lend support to our hypothesis that these kinds of novice judgements are best captured by a reasoning module that is goal-directed but limited in its computational cost (that is, fast and flat) compared with alternatives that vary in terms of computational demands and game reasoning sophistication.

## Decision-making in first-time gameplay

Relative to the flat, single-step thinking that our Intuitive Gamer model posits people use to evaluate new games, conventional models of how people actually play games^6^ perform much deeper tree search, simulating multiple future turns by both players to evaluate potential moves before choosing the best move at each step of a game. But these models have been evaluated only on players who are either experts in a game or have at least played a game many times. Here, we consider what human players do when they are playing a game for the very first time. That is, we ask whether they search deeply, as previous models might suggest, or whether they use only a fast and flat decision mechanism, as our Intuitive Gamer model does—and as our results suggest they do when merely imagining how a game might unfold before playing it.

### What moves do people make?

To test whether the Intuitive Gamer player module captures how people actually play a game for the first time, we recruited 302 participants to play each other in a subset of 40 of the 121 game variations, plus tic-tac-toe (Fig. 1c). Each participant played only a single round of any given game.

**Extended Data Fig. 5:**
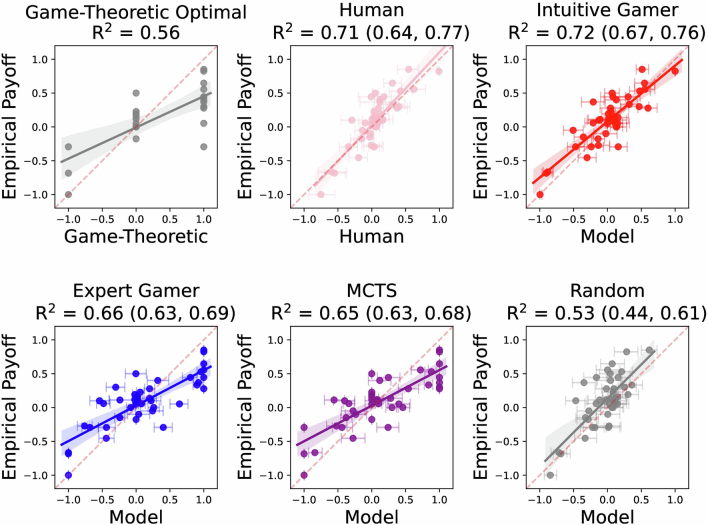
Empirical observed payoffs in actual human-human play against human- and model-predicted payoffs. The empirical payoffs (based on the observed human-human play game outcomes) generally follow the payoffs predicted by people and the Intuitive Gamer model in the “just think” experiment (without any live play). The empirical payoffs do not align with what one would expect under optimal play, nor play under relatively more sophisticated expert models (MCTS or the Expert Gamer) or game agents that use less compute (random).

Initial evidence that the Intuitive Gamer model captures not only how people think about new games but also how they play them for the first time comes from comparing the distribution of actual game outcomes (first player win, second player win or draw) in this gameplay experiment with the outcome distributions to be expected if both players make move decisions according to the model. The model captures most of the variance in actual game payoffs with novice players (*R*^2^ = 0.72 (95% CI: 0.67, 0.76)) and predicts these payoffs as well as human estimates of expected game payoffs do, from our earlier game evaluation experiment (Extended Data Fig. 5).

**Fig. 5:**
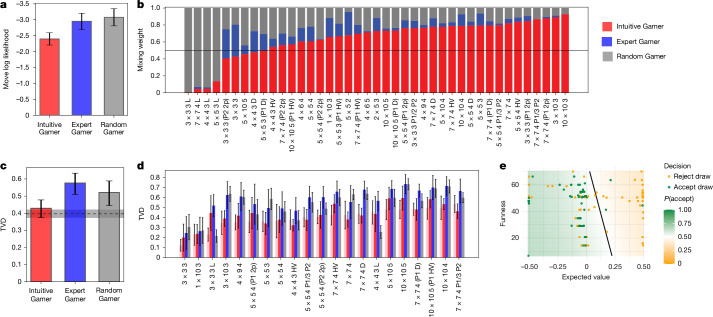
Modelling people’s actions and distribution over predicted actions in the first encounter with a new game. **a**, The average log-likelihood of human moves under three variants of the game player module. Data come from 302 participants playing a single round each of 5 novel games (along with tic-tac-toe); across participants, 40 of our novel games were tested. The Intuitive Gamer model captures players’ moves significantly better than alternatives. Error bars depict 95% bootstrapped CIs. **b**, The estimated contribution of each model in a probabilistic mixture (admixture model; Methods) fit to participants’ moves for each game. The Intuitive Gamer model is the component that best explains participants’ moves in approximately 90% of games (37 of 41); games in which people would not be novices (tic-tac-toe) or some particularly atypical games (for example, misère games, where the first person to get *K* in a row loses, listed with an ‘L’ as in ‘7 × 7 4 L’) are comparatively less well captured by the Intuitive Gamer compared with alternative models. Game descriptions are lemmatized to show board size (for example, 7 × 7) and key features (for example, HV indicates only horizontal and vertical wins are allowed; P2 2p means player 2 can move twice on their first turn; the number after board size indicates the number in a row needed to win). A full description of lemmatized game codes are in Methods. **c**, TVD (lower is better) between human and model-predicted distributions over next moves for the same three models. Judgements come from a new group of 314 participants who estimated where a participant should move next in frozen videos from the gameplay experiment. The dashed line is the split-half human TVD (effectively the noise ceiling). **d**, Mean TVD by game. The Intuitive Gamer generally best matches human judgement distributions, with similar failures to the play data (for example, for misère games). Error bars depict standard deviation over boards for each game. **e**, Decisions of whether to accept or reject a draw when requested are captured by the expected value of the player’s current position, but participants tend to be willing to reject a draw and play out a game with a slightly lower expected value if the game is judged to be fun.

A more direct test is to compare predicted move choices that the Intuitive Gamer model makes at each turn of each game with the actual moves that new players made at the same game steps when they were playing these games for the first time. Because there is a strong element of randomness in people’s move choices as well as the model’s predictions, we evaluated the likelihood of people’s moves under the Intuitive Gamer relative to alternative models that are either more or less sophisticated and computationally intensive, the Expert Gamer and Random Gamer models from our previous study, respectively (Fig. 5a). The Intuitive Gamer model best predicts players’ moves in aggregate, as measured either by average per-move log likelihoods or per-match log likelihoods (summed over all moves within a match), across 1,808 distinct matches with a total of 9,892 moves. All of these differences are highly significant as measured by paired *t*-tests (mean differences in total per-match log likelihood for the Intuitive Gamer versus Expert model = 2.83, *t*_1807_ = 29.4, *P* < 0.001; versus Random model = 3.73, *t*_1807_ = 26.4, *P* < 0.001; mean differences in per-move log likelihood for the Intuitive Gamer versus Expert model = 0.51, *t*_9891_ = 39.9, *P* < 0.001; versus Random model = 0.68, *t*_9891_ = 42.3, *P* < 0.001).

**Extended Data Fig. 6:**
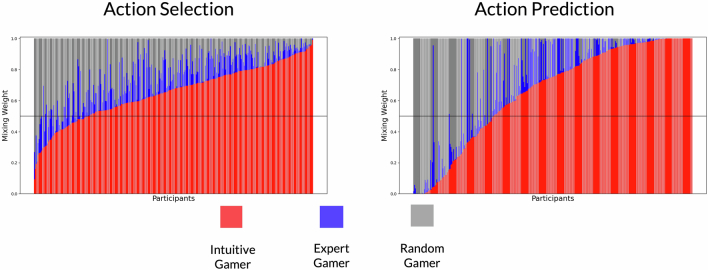
Modeling individual game players. A probabilistic mixture model (admixture) is fit for each individual participants’ action choices (left) and action predictions (right). Bar heights represent the estimated contribution of each model in the probabilistic mixture. The Intuitive Gamer is the dominant component for most participants; however, there is variability across individual participants’ behavior—some participants make action choices more aligned with a Random Gamer or more sophisticated Expert Gamer model.

We also fit these three models of action choice to the distribution of all moves people made in each individual game and the distribution of all moves an individual player made across the different games they played using probabilistic (admixture) models (Supplementary Information). At a per-game level, the Intuitive Gamer model generally, but not always, fits human play better than the Expert or Random models: it captures more than 50% of the probability distribution of moves in 32 out of 41 games, and a plurality of moves in 5 of the remaining games (Fig. 5b). Individual player’s distributions of moves are also best fit by the Intuitive Gamer: it captures more than 50% of the probability distribution of moves for 243 out of 302 players, and a plurality of moves in 16 of the remaining players (Extended Data Fig. 6).

Post hoc exploratory analyses into intermediate stages of gameplay suggest that the Intuitive Gamer model best fits early and mid-stage play, although in later-stage play, greater depth variants are more comparable (Supplementary Information). This could be due to some people being more motivated to think more deeply at the end of the game or finding it easier to think more deeply with a smaller space of moves to consider (as there are fewer legal moves at the end of the game).

**Extended Data Fig. 7:**
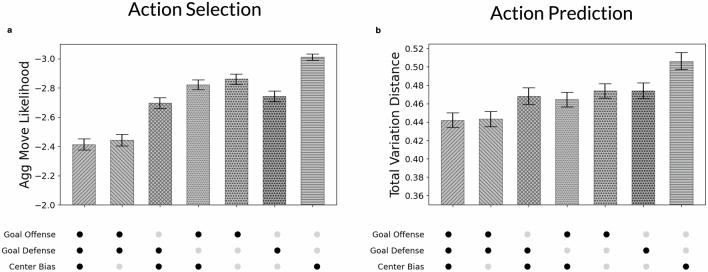
Heuristic value function ablations. To assess the importance of components of the Intuitive Gamer model to fitting participant data, key features of the full Intuitive Gamer value function $\widetilde{{\mathcal{V}}}$ are lesioned by setting the component weight to zero and leaving the others fixed at one. **a**, Aggregate log probability under each variant of the human played moves from the “play” (action selection) experiment; **b**, aggregate TVD comparing the model and human predictor distributions per game stage made in the “watch-and-predict” experiment. Error bars depict bootstrapped 95% CIs over the mean measure per setting. Ablating the connect weight or the block weight has major effects on how well the model explains human data in all three experimental conditions. However, ablating the center weight only has effects in the play experiment. The lack of impact of the center bias in the “watch-and-predict” data may arise from the center bias only mattering in the first moves (and participants are only asked to provide judgments over the third move onwards for the “watch-and-predict” task).

In addition, as in the game evaluation experiments, both aspects of goal-directedness in the Intuitive Gamer value function (offensive progress towards your goal and defensive assessment of how to block your opponent’s progress) matter for capturing human behaviour in novel games (Extended Data Fig. 7). Overall, our results support the hypothesis that when playing a novel game for the first time, people adopt a similar fast, flat and goal-directed approach to choosing their next move as they appear to do in mentally simulating games before they start to play.

### What move should someone else make?

In the previous experiment, as in almost all studies of gameplay, participants made only a single choice for each move, and they played each game only once. Yet our model posits that people think about new problems by running probabilistic mental simulations, letting them form graded expectations about the value of a range of possible actions. To assess how well the Intuitive Gamer player module captures people’s probabilistic expectations, we recruited a new group of participants to predict distributions of likely actions when watching videos of other novices playing these games (Methods and Fig. 1d).

**Fig. 6:**
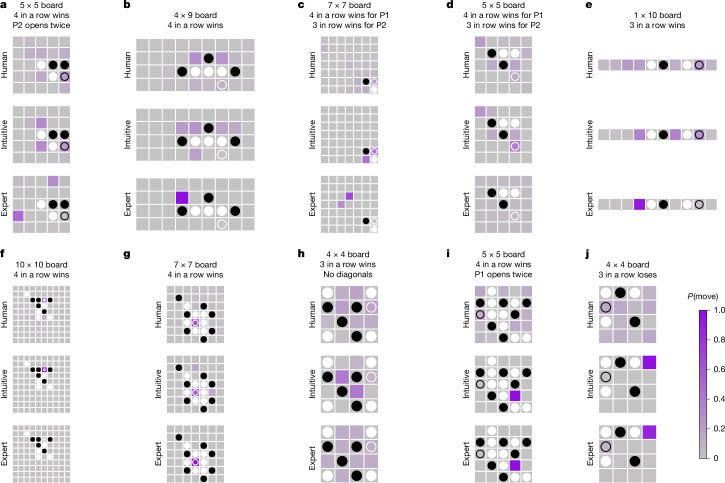
Example human- and model-predicted distributions over the next action in real games. **a**–**j**, Each panel shows a representative state taken from a different game between two human players, along with the actual move one player took at the next step and the predictions made by other humans and two models for how that move should have been made. Filled black and white circles show the moves both players took prior to this point in the game. The one open circle on each board indicates where the human participant whose turn it was on this move (white or black) actually played. The top, middle and bottom boards show the predicted next-move distributions from human participants in the watch-and-predict study, the Intuitive Gamer and the Expert Gamer, respectively. The purple shading gives the predicted probability of choosing each empty square. Humans often distribute probability over several plausible moves (**a**–**e**), although sometimes they strongly favour a single move (**f**,**g**); the Intuitive Gamer often captures this pattern. The Expert Gamer model fits human predictions less well, in some cases because it allocates high probability to a small set of high-utility moves that are often counter-intuitive to naive players (**a**–**c**,**e**); and in other cases by allocating diffuse probability across many states when deep search indicates a sure loss under perfect play (**d**,**f**). The Intuitive Gamer does not always fit best: the Expert Gamer can sometimes better match human confidence (**g**), and the Intuitive Gamer can be too sharp in its predictions (**h**). In some cases, both models are sharper than humans, including crowded boards where people may miss a winning or blocking move (**i**) and misère variants (**j**). The full set of predictions (for 249 moves across 41 distinct games) is included in Supplementary Information.

The Intuitive Gamer model generally better captures the distribution of moves predicted by people compared with alternative models across the 249 game boards for each match and game stage, as measured by the total variance distance (TVD) between model and people’s predicted distribution, and is near the expected noise ceiling (computed via split-half) human judgements for most game boards (Fig. 5c). Differences in distance (where lower means closer to people’s predicted action distribution) are highly statistically significant as measured by paired *t*-tests in the TVD per game board for the Intuitive Gamer versus Expert model = −0.15, *t*_248_ = −15.0, *P* < 0.001 and versus Random model = −0.09, *t*_248_ = −7.5, *P* < 0.001. Exceptions for poor fits align with those found in the play experiments (for example, on misère games; see Fig. 5d and example boards in Fig. 6). Fits are robust to choice of distributional measure (Supplementary Information). People assign similar log probability on the move actually played by the human player to that of the Intuitive Gamer, further highlighting alignment of the Intuitive Gamer to people’s probabilistic judgements (Supplementary Information).

### When is a game worth continuing?

Game evaluation is important not only when deciding whether a game is worth playing but also for assessing—during play—whether a game is worth continuing to engage with. In our gameplay study, participants had the option to request a draw at any point in play. If they received a draw request from another player, they had the choice of whether to accept the draw and immediately terminate play or reject the draw and keep playing. Over the 1,808 matches participants played, players made 142 draw requests, of which 83 were accepted and 59 were rejected. We next considered how people decide whether or not to keep playing when they receive a draw request.

**Extended Data Fig. 8:**
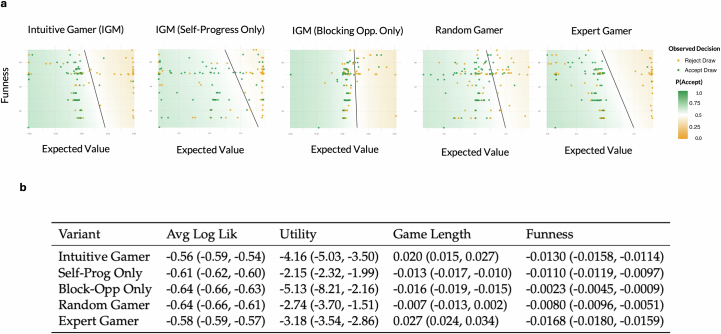
Modeling people’s decisions about whether to keep playing when they receive a draw request. **a**, Logistic regression models predict whether a player would accept or reject a draw request if received are fit to the expected value of continuing to play under the respective player module variants, and the expected funness of games (as predicted by people in the just-think experiments). Decision surfaces are plotted, averaged over 100 bootstrapped samples of expected value calculations over *k* = 6 simulations. The Intuitive Gamer and Expert Gamer qualitatively encode the intuition that games that have higher expected value from continuing or are expected to be fun are games where players are more open to continuing to play. **b**, Coefficients for draw request models, depicting 95% CIs over bootstrapped subsamples of *k* = 6 game simulations. The actions taken by a player (whether to accept or reject a draw) are assessed under the models by computing the log likelihood of doing the action, given the features computed over the particular board. The model was trained to predict whether a draw request would be accepted; therefore, negative weights for features mean that a higher value of that feature is more predictive of rejecting a draw (i.e., wanting to continue a game). Log likelihood is computed as the averaged likelihood assigned to each decision and averaged over bootstrapped model fits. Higher likelihood means better fit. Using the expected value of continuing as computed under Intuitive Gamer and deeper Expert Gamer simulations similarly explain people’s decisions of whether to accept or reject a draw. Lesioning either or both of the goal-directed components of the Intuitive Gamer value function substantially impairs fit to people’s decisions, particularly when lesioning the offensive component (i.e., the component specifically driving a player to make progress towards their own goal). This may be because, upon receiving a draw request, a player decides whether to accept or reject from a more offensive stance.

We modelled these choices as probabilistic value-based decisions trading off a player’s expected reward of winning with the expected cost of continuing to play, both of which can be computed under the Intuitive Gamer reasoning module conditioning on the current board state (rather than a blank opening board, as in our earlier game evaluation studies; Methods). In exploratory analyses, we also hypothesized that a player might be willing to keep playing a game even if the expected cost of continuing outweighs the potential objective monetary reward, if playing provides an alternative subjective reward, for example, if it is likely to be fun. A logistic regression model fit to participants’ draw request decisions using these three features (Methods) captures the intuition that players’ game-time evaluations are probabilistically rational under limited resources (time) and sensitive to subjective factors such as the engagement of games in addition to their objective value (Fig. 5e). Ablations of the Intuitive Gamer evaluation function lead to significantly worse qualitative and quantitative fits to human draw request choices (Extended Data Fig. 8a,b).

## Discussion

Systems of rules and rewards govern many aspects of human behaviour—from jobs and institutions to games and geopolitics. A core question is, how can people think reasonably about any such system or problem for the first time, so flexibly and quickly? We formalized and empirically evaluated a computational model of an Intuitive Gamer, which draws on fast and flat goal-directed probabilistic mental simulations to make rich quantitative predictions about how novice players judge the objective value of a previously unseen game, as well as to make more subjective evaluations. Our model also captures how people act within these games, and does so better than alternative models with more and less computational power. The same model generalizes across these different tasks and the many different games we study.

Our work differs from most previous studies of gameplay in cognitive science and AI, which focus on how expert play is achieved, and typically consider only expertise in a single game. By contrast, we study a class of 121 strategic games with varying dynamics and highlight the phase of ‘pre-expertise’: the critical first moments before people can rely on the benefits of repeated experience, but when they must nonetheless take reasonable actions and even decide whether to play at all—or to continue playing. Studying pre-expertise across varied domains is important not just for our understanding of cognition generally, but also to construct more accurate models of human behaviour in game theory under novel economic mechanisms^31,32^ or alongside unfamiliar agents such as AI systems^33,34^. The Intuitive Gamer approach could inform the design of more efficient AI systems that make reasonable judgements in new multi-agent settings despite using much less compute than conventional expert-level models.

Our work also differs from prior studies of funness in games, which have focused on identifying factors that lead people to experience a game as fun. By contrast, we have focused on modelling the intuitive theories people have about how they would expect to experience a game as fun, before playing it for the first time. Features identified in the previous literature, such as strategic depth^35^ and competence^36^ reflecting the need for challenge, or learning progress^37^ and thinking progress^38^, do align with those in our model, especially the reward for thinking component. Future work could study whether other aspects of motivation identified in previous gaming literature—autonomy, social connectedness, immersion and ease of control^36^—also contribute to people’s intuitions about funness, and whether these could also be modelled using a generalization of our Intuitive Gamer model.

As a model of human reasoning, our work at present is limited to a subset of perfect information, two-player competitive board games, and these games reflect novelty within a genre that many people are familiar with rather than completely novel experience. It is an open question how the heuristics our model uses could generalize to more complex games in the same class, such as Go or chess, and to entirely new classes of games. Many extensions are possible, such as generalizing the contiguous-line features that we consider towards connectivity or territory metrics that reflect partial or graded progress towards a game’s win conditions. Natural language could be used to suggest short programs expressing such heuristics, as in verifiable code-based rewards in reinforcement learning^39^. Although we hypothesize that key features of the Intuitive Gamer reasoning module—being fast, with a few probabilistic simulations of play, where play is assumed to involve goal-directed agents—would extend to other game classes, it is an open question how ‘flat’ reasoning might be and what more general heuristics might capture intuitive reasoning in the wider range of games that characterize human social life. Understanding peoples’ general game reasoning on a wider class of games beyond competitive settings, for example, cooperative or multi-agent contexts, is necessary to move towards a general model of people’s ‘intuitive theory of games’, analogous to the computational models of intuitive theories of physics or intuitive theories of mind that cognitive scientists have constructed^40,41^.

In addition, there is a need for more fine-grained process-level and individual-level accounts of how people reason about novel games. More work should study how people judge the expected outcome of a game if they stop imagining play before reaching a win or draw, if their simulations are temporally dependent, or when they do not simulate at all—and how models can best capture these complexities. Our model also does not address how people may learn and adapt such heuristics within or across games, limitations that suggest important directions for future work. How mental computation for reasoning about familiar games may shape thinking about a new game^42^, especially in high-stakes situations^26^ or when time is limited or thinking is costly^19,20^, and how in-game experience^43,44^ and preferences for different kinds of play and strategy^45^ influence game reasoning, will also probably be important for modelling variability in peoples’ thinking.

Finally, people also make new games and new problems, either by modifying old ones to make them more challenging or more fun, or by creating entirely novel goals and pursuits—which has led to some of humanity’s greatest discoveries and innovations^38^. In ongoing work, we are exploring whether models based on fast and flat simulation can also explain the moves people make in these ‘meta-games’ of game-creation and modification.

Science itself is such a ‘meta-game’: a family of games against nature^46^, where scientists set the rules that lead to the most productive research^47^. Similarly, mathematicians may explore conjectures and proof strategies by imagining adding or relaxing the logical rules of how a problem is formulated^48^. Suppose, more speculatively, that these and other creative activities at the frontiers of human knowledge do in fact share something deeply in common with intuitive reasoning about games, or even children’s play. Then, perhaps mechanisms such as the fast probabilistic mental simulations studied here could help to explain aspects of how researchers choose to approach a problem, or which problems to think about in the first place. Whether scientific thinking can be meaningfully captured by such models is very much an open question, but one we are now better positioned to investigate. At the very least, it feels like a game worth playing.

## Methods

### Game construction

We manually constructed the 121 two-player competitive strategy game variants played on *M* × *N* grids, where *M* is the number of rows and *N* is the number of columns. One goal in creating these games was to ensure there is enough diversity in board size and rule structure as well as systematic variance. To that end, we designed a series of variations on square boards: on 10 × 10 boards, we have *K*-in-a-row for *K* from 2 to 10; for 3-in-a-row, we have *M* by *N* = *M* boards from 3 to 10; we also include a few 5 × 5 boards with varying *K*. We additionally included a few other square boards varying in complexity, as well as rectangular boards ranging in size from from 1 × 5 to 5 × 10, and we have integer 2 ≤ *n* ≤ 6 for *K*-in-a-row. To assess how people reason about games that are not physically realizable, we included three ‘infinitely’ sized games with *K* = 3, 5 and 10 for *K*-in-a-row. These categories give us 41 games with typical ‘*M*–*N*–*K* rules’ (for example, where the players take turns and have the same objective: ‘make *K* in a row, where horizontal, vertical, or diagonal all count’). This set includes the standard tic-tac-toe as well as 4 × 9, 4 in a row wins from ref. ^6^. We then created a number of games with varied game rules. We kept the selection of board sizes and *K*-in-a-row fixed across categories (ten games within each category). The selection included 3 × 3, 4 × 4, 5 × 5 and 10 × 10 boards and *n* ∈ {3, 4, 5, 10}. We also designed games with more atypical rules, varying the winning conditions (for example, *K*-in-a-row loses and diagonal connections do not count as wins) and first-mover dynamics (for example, player 1 can place two pieces on their first turn). These categories give us an additional 80 games, totalling the 121 in our dataset. We manually implemented an automated win condition checker that permits flexible game assessment over all game types.

#### Game name codes

Games are expressed in abbreviated form throughout the paper. Games are described by their board size (rows × columns) and the number *K* in a row to win. For example, ‘4 × 4, 3’ means the game is played on a 4 × 4 grid and the first person to get 3 pieces in a row wins. Unless otherwise stated, horizontal, vertical and diagonal all count. If only horizontal and vertical 3 in a row count, the game would be written as ‘4 × 4, 3 HV’. If only diagonal counts, then the game will be written as ‘4 × 4, 3 D’. If the constraint only applies to one player (for example, P1 can only win horizontally and vertically, and P2 can win any way), that is represented as ‘4 × 4, 3 (P1 HV)’. If one player can go twice on their turn, for example, the second player (P2) can play twice, that is written as ‘4 × 4, 3 (P2 2p)’. A misère game (where first to *K* in a row loses) is written with an ‘L’, for example, ‘4 × 4, 3 L’. In our dataset, multiple rule modifications cannot co-occur, so each game can be expressed in this abbreviated way.

The full game natural language game descriptions were provided to participants (for example, as shown in Extended Data Table 1). The game codes are used only for ease of presentation in this paper.

### Human experiments

All human experiments were conducted under prior approval from the institutional review board at the Massachusetts Institute of Technology through the Computational Cognitive Science Lab. All participants provided informed consent.

#### Zero-shot outcome evaluation experiment

We recruited 238 participants from Prolific^49^ to judge novel games. Each participant was randomly presented with 10 games sampled from our 121 diverse game stimuli, as well as regular tic-tac-toe (won by making 3 in a row on a 3 × 3 board) to set baselines for game judgements. We collected approximately 20 judgements per game stimulus for each game reasoning query. Participants were paid at a base rate of US$12.50 per hour with an optional bonus up to US$15 per hour; the full experiment approximately took 25 minutes.

Participants were instructed to evaluate likely game outcomes. Specifically, participants produced judgements on a continuous 0 to 100 probability scale to predict the likelihood of a first player win (“If the game does not end in a draw, assuming both players play reasonably, how likely is it that the first player is going to win (not draw)?”) and a draw (“Assuming both players play reasonably, how likely is the game to end in a draw?”). Judgements were made using sliders. Both game outcome question sliders appeared on the same page.

Participants produced judgements about each game based on a linguistic game specification. We additionally provided participants with an interactive scratchpad board that they were told they could, but were not required to, use to inform their judgements. The scratchpad was automatically sized to the board of the game specified; infinite boards were presented on a 13 × 13 grid with dashed lines indicating the board could continue. The scratchpad permitted automatically placing pieces of different colours (‘red’ and ‘blue’ to simulate different players); participants could force the the next play to be made by the same player (for example, colour red twice in a row) by pressing the spacebar. Two buttons appeared below the scratchpad, permitting the user to either undo their last move or clear the screen to begin a new ‘game’. See Supplementary Information section 4 for examples of the experiment interface. Participants were required to consider each game for at least 60 s before being allowed to make their game judgements. Outliers were determined as the 10% of the judgements farthest from the mean judgements of other participants (in terms of the summed distance from the two queries) and filtered out for each game.

After answering all game reasoning queries, we additionally asked participants to create a new grid-based game variant that they would find fun. Participants wrote a linguistic game specification, describing the board size and win conditions. As in the game judgement queries, participants were again provided an optional scratchpad and required to spend 60 s before submitting a response. The scratchpad enabled participants to try out the game they intended to create. After specifying a game, participants were asked to answer the same game reasoning query (either game outcomes or game fun) about their own game. These game-generation responses were not studied here and are actively being explored in a follow-up study. Example screenshots of experiment interfaces are included in Supplementary Information section 4.

#### Zero-shot funness evaluation experiment

We repeated the same methodology above with a new group of 257 participants, replacing the game outcome questions with a question about game funness. Participants instead assessed the expected funness of the game (“How fun is this game?”) on a confidence scale spanning 0 (the least fun of this class of game) to 100 (the most fun of this class of game). 11 participants were filtered due to having provided non-effortful or AI-generated games in the creation stage, resulting in a total of 246 participants, and the same outlier filtering was applied per trial as in the outcome evaluation.

#### Zero-shot human–human play experiment

We recruited 302 participants to play these novel games in a pre-registered experiment. We selected a subset of 40 games from the full set of 121 to span a representative range of the gameplay variations (board shapes, board sizes and win rules) in the original dataset while generally favouring games that would not take very long to play in a live experiment. We randomly constructed eight batches of five of these games. Each participant additionally played one round of tic-tac-toe. The order of games was shuffled for each new set of participants.

Each participant was automatically paired with another player. We developed our interface using Empirica^50^, which supports synchronous human–human pairing. Participants played one round from five different games. Players were informed they would get a bonus of US$0.50 for every win. Participants had to spend at least 5 s reading the game description before they began. We appended ‘Horizontal, vertical and diagonal all count’ to all game descriptions where any direction was allowed after we noticed some participants in pilots were confused as to which line directions would result in a win. Players were randomly assigned to move either first or second and a corresponding piece colour (red or blue). Players took turns making moves on the synchronous game interface. Players had no time limit on their turn.

Players were also allowed to request a draw or decide to surrender using buttons at the bottom of the interface. If a player surrendered, the game ended immediately (and that player lost). If a player requested a draw, the other player was allowed to either accept the draw (after which the game ended immediately and no player won) or reject the draw (leading the game to continue being played). Draw requests appeared as a popup banner for the other player. We include screenshots of the interface in Supplementary Information section 4.

The match ended when either a player won, a player surrendered, the board filled up completely (draw), or the players agreed to a draw. Both participants were informed about the game outcome. After each match, players made a judgement about either the expected outcomes of that game overall (with a new set of reasonable players) or the game’s funness (in a new match against a new player). Each pair of players was randomly assigned to either the outcome or funness rating condition. Judgements were made on a slider. Players were also presented with a ‘frozen’ version of the match on an example board with which they could replay all of the moves they and their opponent had made. Players also indicated how skilled they thought their opponent was at this game (“Out of 100 other random new players, where do you think the opponent you just played would rank in skill for this game?”). After the judgements were made, the players continued to the next, new game. At the end of the study, they filled out a text-based survey providing general information on their strategy and how fun they found the experiment. We filtered out 18 participants who did not pass our quality control (that is, they provided judgements that were ‘standard’ values (near 0, 50 or 100) on 80% or more of judgements) for a total of 284 subjects.

#### Watching and predicting play experiment

We recruited a new set of 314 participants, in a pre-registered experiment, to reason about the games zero-shot from only indirect experience: watching two other agents play. We selected a subset of 20 of the games from the previous human–human play study to ensure representation across game rules and dynamics. We also included tic-tac-toe (totalling 21 games). Participants watched a series of videos of other agents’ gameplay. Each video involved two humans playing each other, sourced from our live human–human gameplay experiment. We sampled four human–human played matches randomly from each of the 21 games (owing to a randomized batching error, only three unique matches were sampled for tic-tac-toe; hence, 249 game boards over the matches from 21 games and three stages per game), after filtering out any matches that ended preemptively from a draw request or surrender. For each match, we sampled three specific boards to be evaluated corresponding to the beginning, middle and end of the match. For the beginning and end boards, we randomly selected either the third or fourth move and the second-to-last or third-to-last move, respectively. For the middle board, we selected the median move. We filtered out any match that ended before eight moves. Participants watched one match from five different games, plus tic-tac-toe.

Before each match, participants were informed of the game rules and required to think about the rules for 5 s before the video began. We again appended “Horizontal, vertical, and diagonal all count” to all game descriptions where any direction was allowed. Videos played forward at a fixed rate, as in ref. ^6^. We chose 2 s per move to give viewers enough time to process each move without taking too long overall. Each video was stopped at the three time points described above. At each stopping point, participants indicated their belief over where they thought the acting player should move next. Participants were given five clicks, which they could spread across the legal moves on the board to indicate their confidence that the player should move there. We chose five clicks to balance granularity of the elicited belief distribution against the burden on the participants. After each click, the opacity of the cell increased to indicate higher confidence. Participants were informed of the number of clicks they had left and could reset their clicks by clicking on a button below the interactive board.

After watching each video and indicating where they thought a player should move at each of the three timepoints, participants were then shown the remainder of the game as a board snapshot (cells indicated where players had moved and the order of play). Participants then answered either the same game outcome or funness judgements about the game overall, as described above. Judgements were made on a slider. Participants also indicated how skilled they thought each of the players was. We filtered out 10 participants who did not pass our quality control, leaving us with a total of 304 valid participants.

### Problem formulation

Formally, one can think of a system, problem, or game *G* in terms of a space of feasible states ${\mathcal{S}}$; possible actions ${\mathcal{A}}$; rules ${\mathcal{T}}$ specifying valid actions and state transitions, and governing the overall dynamics; and goal functions mapping from states ${\mathcal{S}}$ to possible rewards ${\mathcal{R}}$. We are interested in how a reasoner infers properties of a new game *ψ*(*G*) (for example, whether a game is likely to end in a draw or have a bias towards a particular player; whether the game is likely to be engaging and fun) as well as a policy *π*_*G*_(*a*_*t*_∣*s*_*t*_) for how to play (choosing actions given the current state at time *t*, to achieve their goal, for example, to win), without experience of actual traces of gameplay and relying instead on simulated or imagined traces. Our aim is to model how people approximate *ψ*(*G*) and *π*_*G*_ in a way that can (1) take in any *G* as input, and (2) do so with a limited compute budget and no direct experience with game *G*.

### Intuitive Gamer model specification

We first describe a formal account of the Intuitive Gamer player module, and then describe the reasoning module.

#### Intuitive Gamer player module

Formally, at a given board state (${s}_{t}\in {\mathcal{S}}$), the Intuitive Gamer player module scores all legal next actions (${a}_{t}\in {\mathcal{A}}$) according to a measure of immediate progress made towards a player’s own goal (*U*_self_) and a measure of progress blocked (*U*_opp_) towards the opponent’s goal (Fig. 2c). Progress is based on the extent to which an action connects more contiguous pieces towards a winning *K*-in-a-row configuration. These two utilities are designed to capture the general intuition that game players aim to make progress towards their goal while preventing their opponents from doing the same. In addition, we can consider other easily computable heuristic functions that may bear on the value of an action (*U*_aux_, for ‘auxiliary’). In our experiments, we consider an auxiliary heuristic based on proximity to the centre of the board. It encodes a ‘centre bias’—a preference for making moves near the centre of the board, which allows a piece to participate in more winning terminal configurations. These heuristics are drawn from features used by previous studies in similar games^6,29,30^, although we generalize them to our broader class of strategic games. The final heuristic value assigned to a given action on a particular board, $\widetilde{{\mathcal{V}}}({s}_{t},{a}_{t})$, is a sum of the three utility components described above:

1 $$\widetilde{{\mathcal{V}}}({s}_{t},{a}_{t})={U}_{\mathrm{self}}({s}_{t},{a}_{t})+{U}_{\mathrm{opp}}({s}_{t},{a}_{t})+{U}_{\mathrm{aux}}({s}_{t},{a}_{t}).$$

Our approach to action evaluation and choice reduces computational cost, as it is strongly local in both space and time: it considers only the progress immediately stemming from a specific action and does not account for either past or potential future returns. By contrast, it is common for value functions to be action-agnostic (that is, to depend only on *s*_*t*_ (ref. ^6^)) and to explicitly capture some notion of the game’s terminal rewards (for example, via a learned value network^4^). Intuitively, each of these alternatives represents a substantial increase in cognitive load: the former requires scanning over the entire board to evaluate any action, and the latter requires mental simulation all the way to the end of the game (or access to a function that is derived from such simulations) before deciding what action to take.

We next describe our specific implementation of each utility function. As mentioned, we came up with these heuristics via prior literature grounding, as well as expert intuitions. Concepts of progress and blocking are present in the classic study of ref. ^29^, and similarly progress patterns and central tendencies are used in the recent ref. ^6^, on which we base our expert model. More generally, features based on connections (or lack thereof), implemented differently based on games, is pretty common in game-playing AI. Most of the authors are expert cognitive modellers and some are expert board game players (and played many connect-*N* style games), so the combination of progress, blocking and centring is effectively the first modelling hypothesis that we came up with and judged to be plausible. More details on the lesioning of these heuristics are included in Supplementary Information section 3.3.

The first utility (*U*_self_) computes a measure of intermediate ‘goal progress’ of the active player, based on a feature *n*_1_ defined as the largest line of contiguous pieces created by the action that could be extended to result in a win. This means that actions which extend a line in an illegal direction (for example, a diagonal line in a game with only horizontal or vertical wins) or that are already blocked by an opponent’s piece or the edge of the board do not contribute to goal progress. If the action would result in a win for the active player (that is, *n*_1_ = *K*) then an additional 1 is added to the feature to magnify the value of winning actions. We note that our choice of feature only rewards actions that form contiguous lines of pieces—any piece that is not immediately adjacent to a previously placed piece from the active player has *n*_1_ = 0. Although other formulations (such as rewarding actions that form the ends of a line that is empty in the middle or detecting particular patterns of pieces on the board) are sensible, we choose a simple and easy-to-calculate function that reflects a straightforward intuition (that is, making *K* in a row by first making *K* − 1 in a row, *K* − 2 in a row and so on).

The second utility (*U*_opp_) computes a measure of ‘progress blocked’ for the opponent, based on a feature *n*_2_ that is largely symmetric with the goal progress feature above: it is computed exactly as the goal progress the opponent would obtain if they made the same move (that is, with respect to the opponent’s allowed winning directions). We subtract 0.5 from *n*_2_ to reflect people’s tendency to weigh offence over defence^29^, so blocking the opponent’s $\hat{K}$ in a row is not as good as making $\hat{K}$ in a row for oneself (but is better than making $\hat{K}-1$ in a row). However, if *n*_2_ equals the winning *K*, we do not subtract 0.5 in order to account for the importance of blocking winning threats. As above, there are other reasonable formulations of this feature that we leave to future work.

Finally, the third utility (*U*_aux_) is computed as the normalized Euclidean distance between the position of the action and the centre of the board, *ξ* ∈ [0, 1]. This reflects the intuition, applicable across our family of games, that people often place pieces around the centre of the board. This tendency may in part be explained by the fact that placing pieces closer to the centre of the board allows that piece to participate in the most possible winning terminal states for any *K*-in-a-row win condition. This measure generalizes to arbitrary rectangular boards. For example, on a 4 × 6 board, intuitively the ‘middle point’ is the centre, and the middle four cells are all closest to the centre (⟨2, 3⟩, ⟨2, 4⟩, ⟨3, 3⟩, ⟨3, 4⟩).

We assume each utility function is represented as an exponentiation of the respective feature:

2 $$\widetilde{{\mathcal{V}}}(s,a)={w}_{\mathrm{connect}}\times {2}^{{n}_{1}}+{w}_{\mathrm{block}}\times {2}^{{n}_{2}}+{w}_{\mathrm{centre}}\times {2}^{(1-\xi )}.$$

The choice to exponentiate some measure of progress for heuristic functions is common in gameplay modelling (for example, refs. ^51–53^). We chose base two on the basis of light initial exploration (prior to collecting any human gameplay data), under the goal of keeping our intuitive model simple. Future work should explore other modelling choices to capture how people might represent and combine multiple utility functions, including the role of learning in how people might come to flexibly synthesize these functions.

Moves are selected by following Boltzmann rationality^54–56^, sampling actions from a softmax function over their estimated value (based on the above goal-directed heuristics). We assume that players have already developed the capacity to account for multiple goals simultaneously, unlike potentially even more naive child-like game reasoners^29^. Concretely, the probability of choosing action $\hat{a}$ at state *s* is given by:

3 $$P(\hat{a}| s)=\frac{\exp (\widetilde{{\mathcal{V}}}(s,\hat{a})/\tau )}{\sum _{a}\exp (\widetilde{{\mathcal{V}}}(s,a)/\tau )}.$$

We fix temperature (*τ*) at 1 for our game reasoning and action experiments (with the exception of marginalizing over *τ* only for the admixture analyses in the ‘play’ experiment). We set the weights of each component (*w*) to 1 for all experiments. We use the same settings for the Expert Gamer model (as it uses the same value function and softmax-based action selection). We find that equal weights is a reasonable fit for the Intuitive Gamer model to human payoff predictions (Supplementary Information section 3.3). When lesioning a component of the value function, we set its weight to zero.

#### Intuitive Gamer reasoning module

The Intuitive Gamer player module is queried as part of the game reasoning module. The Intuitive Gamer reasoning module nests player module-based simulations to compute a series of gameplay traces ($\{({s}_{0}^{0},{s}_{1}^{0},\ldots ,{s}_{T}^{0}),\ldots ,({s}_{0}^{k},{s}_{1}^{k},\ldots ,{s}_{{T}^{{\prime} }}^{k})\}$). These simulations involve self-play between the same player module type (with the exception of the funness reward for thinking computation, see below), where each agent is trying to make progress towards their own goal (which may be different, for example, player 1 trying to make 4 in a row only diagonally, while player 2 can win in any direction). From these simulations, a probabilistic judgement of the expected outcomes can be made. That is, for each game *G*, *k* game simulations are sampled, producing a set of *k* outcomes *o* ∈ {−1, 0, 1} encoding whether the first player won (1), lost (−1), or the game ended in a draw (0). From these outcomes, we can compute a payoff of a game *G*:

4 $$\begin{array}{c}\psi (G)=(1)P(\mathrm{win}| \neg \mathrm{draw})\cdot P(\neg \mathrm{draw})\\ \,+(-1)\cdot P(\mathrm{lose}| \neg \mathrm{draw})\cdot P(\neg \mathrm{draw})+(0)\cdot P(\mathrm{draw}).\end{array}$$

5 $$\psi (G)=P(\neg \mathrm{draw})\cdot [P(\mathrm{win}| \neg \mathrm{draw})-P(\mathrm{lose}| \neg \mathrm{draw})].$$

Our game reasoning module is constructed to represent the reasoning of any one participant. Therefore, we draw *k* simulated matches for *N* = 20 simulated participants (as each game has, generally, 20 participant responses; see ‘Analysis methods’ below for details on selecting *k*).

#### Partial game simulations

Our primary game reasoning module assumes that mental simulations are conducted until the end of the game is reached: either a player attains the win condition, or all open board positions are filled and the game is called a draw. While many of our games do not take many moves to reach an end, people might not mentally simulate all the way to the end of the game in each of their *k* simulations. We conduct a preliminary exploration into the impact of partial game simulations in fitting peoples’ game fairness evaluations by modelling the possibility that people halt any one of their *k* simulations early. We sample a ‘stopping time’ uniformly from 1 to the size of the board and end the game after that many turns. To determine the outcome of games that are stopped early, we apply a simple rule and treat each of them as a draw (allowing us to explore encoding a weak prior towards games ending in draws; Supplementary Information section 5.4). We repeat the same exploration of variance (see ‘Analysis methods’ below) and find that the optimal number of samples is similarly more than 1 and less than 10, but may be closer to *k* = 4 (Extended Data Fig. 2). We leave further analysis of the distributions over stopping times and how people assign outcomes for partial games to future work.

#### Funness model

To estimate a game’s funness, we consider three features derived from playouts under the Intuitive Gamer player module: balance, reward for thinking and game length. We define a game’s balance as the difference between the probability distribution of observed outcomes and an ideal outcome distribution relative to a game where exactly half of the games end in wins and losses and none end in draws (measured by the Earth Mover’s Distance^57^). As described in the main text, this feature captures the notion that players prefer decisive games (that is, those that do not end in draws) that are also balanced across players^58–60^. We measure game length as the expected number of moves until a game ends in a draw or win, computed from the same Intuitive Gamer simulations. The effect of game length on funness is modelled using a quadratic function, based on the intuition that the most fun games will be neither too short nor too long. We define a game’s reward for thinking as the proportion of simulated games won by the Intuitive Gamer model playing against the Random Gamer player module, that is, a player that chooses actions uniformly at random from any legal move. This feature captures the intuition that players prefer games that they expect will challenge their thinking^35,38^ and reward them for at least some strategic thinking, rather than just responding arbitrarily or without any strategy.

We use 120 game simulations for each feature (to align with the *k* = 6 simulations for each of 20 participants), randomizing whether the Intuitive Gamer plays first against the random agent in the simulations used to assess reward for thinking. Game simulations are run to the end of the game (where either a player wins or the game ends in a draw); future work can explore variants of the funness model in which features are computed under partial game simulations. We use the same features and number of simulations when comparing to alternative models (see below in ‘Fitting regression models to funness judgements’).

### Alternative models

We next detail several alternative models.

#### Expert Gamer

We compare our Intuitive Gamer model against an Expert Gamer model that differs along two key dimensions: (1) sophistication of the value function, and (2) depth of search. This model is closely based on the model of human expert play for 4-in-a-row on a 4 × 9 board in ref. ^6^, which empirically estimates tree search depth from human players after hours of continuous gameplay experience. As in ref. ^6^, the depth is controlled by a probabilistic ‘stopping parameter’ governing how many the search tree is expanded; we run all simulations by expanding the search tree a fixed number (*k*_iterations_ = 636) of iterations, where *k*_iterations_ = 636 is the empirical mean value of this parameter estimated from the ref. ^6^ data. This setting corresponds to approximately depth-5 search. On each iteration, the Expert Gamer selects a node (corresponding to a board state). We then expand this node, considering all legal actions, wherein we compute the value of each action as outlined below. The Expert Gamer model then conducts best-first search over the game tree, using its state value function defined above. Specifically, it probabilistically expands nodes in the search tree by repeatedly sampling actions from a softmax policy governed by temperature *τ* over its current action estimates based on these computations, expanding unexplored states in the search tree, and backpropagating utilities estimated at future states. As in ref. ^6^, any node that ends in a definite win or loss is assigned ±*σ* based on whether the move would result in a win for the current play (+) or loss (−). We set *σ* = 1,000.

We next detail the Expert Gamer value function. Following ref. ^6^, we compute the value of any move by looking at features over the entire board state, rather than locally circumspect around the possible move position in question (like the Intuitive Gamer model). This is more computationally intensive, particularly for larger boards. Specifically, for any open legal position *p*, we imagine a state *s*′ that has that position played by the current player. We then sweep over all played positions with that board state. For each played position *p*′, we compute the same novice, partial-progress value function. We then define the value of that state *s*′ as the difference in cumulative value from the played positions by the current player minus the opponent. We set the value function feature weights (*w*_connect_, *w*_block_, *w*_centre_) using the fit values from the IntuitiveGgamer model. It is worth noting that the Expert Gamer is itself an important contribution—it is a more general version of a relatively deep goal-directed model. We leave validation of how well the Expert Gamer captures human expert reasoning and play for future work.

#### Random Gamer

The Random Gamer player selects actions uniformly over the space of legal moves. Games are simulated to the end (for example, until either player wins or the game ends in a draw).

#### Monte Carlo tree search

We additionally implement a standard upper-confidence-bound MCTS^27,28^ agent to act as an approximate gameplay ‘oracle’ (pseudocode can be found in Supplementary Information section 2). Unlike the Intuitive Gamer and Expert Gamer models, MCTS does not use game-specific heuristic features and instead estimates intermediate utilities by expanding a search tree guided by repeated random rollouts to terminal states. MCTS algorithms are commonly used to approximate optimal gameplay in arbitrary games^28^, as they are empirically both efficient and accurate. Specifically, the MCTS implementation we consider here uses a large number of tree expansions (10,000) to model the behaviour of a near-perfect player in our novel games. Owing to computational costs, we estimate the expected payoffs using 50 simulations per game; these samples are then bootstrapped in their fit to people, as with the other models.

#### Comparison of MCTS and Intuitive Gamer

We briefly clarify the relation between MCTS—the planning algorithm behind many AI systems that achieve expert-level gameplay such as AlphaGo and AlphaZero—and the contribution the Intuitive Gamer model offers as a model of human reasoning. One could potentially view the Intuitive Gamer’s player module as a particularly restricted or shallow variant of MCTS (as MCTS is also compatible with constraining computational resources and adopting heuristic functions). However, for several reasons we think it is important to distinguish between these classes of models—recognizing that this is in part a matter of scientific emphasis and interpretation rather than algorithmic innovation.

First, framing MCTS so generally as to include the Intuitive Gamer’s play would also include almost any kind of stochastic tree search, including other models in our comparison set, for example, the Random Gamer. More deeply, it would leave out core features of MCTS that have made it so powerful in AI systems as well as the most distinctive features of the Intuitive Gamer as a cognitive model. MCTS made such an impact in AI gameplay and reinforcement learning^4,14,27,28^ precisely because it could achieve very strong play without the need for heuristics to guide search. Instead, it uses extensive inference-time computation and sophisticated tree traversal arithmetic (backtracking, node visit counting) to effectively explore a very large, unstructured game tree. This is in contrast to the Intuitive Gamer, which does not require any sophisticated algorithms and uses very minimal computation, although it does rely on a small number of abstract heuristics when assessing the value of next moves. Such a design leads the Intuitive Gamer to be a highly efficient if less than optimal player, and one which we believe much better captures how people reason (as evidenced in our behavioural action selection and action prediction studies). As described in the ‘Resource-rational reasoning’ section of the main text and captured in Extended Data Table 2b, the Intuitive Gamer is orders of magnitude more efficient than either MCTS (run for 10,000 iterations, as described above) or the Expert Gamer, in terms of wallclock time and number of board states evaluated.

#### Variations on the full Intuitive Gamer model

We compare the full Intuitive Gamer model against several variants that modulate whether it is flat, goal-directed or probabilistic. The non-flat version of the Intuitive Gamer model performs a deeper search over possible moves when selecting actions. The non-goal-directed version of the Intuitive Gamer model ablates one or more of the value function components (that is, player progress or blocking progress—see Supplementary Information for ablations of the centre-bias component). The non-probabilistic version of the Intuitive Gamer model replaces the softmax element of action selection with a deterministic choice (or equivalently, softmax with temperature zero). We further vary fastness by conducting a sample complexity analysis wherein we modulate the number of simulations drawn (*k*) from the gamer model, over which the game reasoning engine computes the expected payoff (Extended Data Fig. 1).

#### Game-theoretic optimal payoffs

Many of the games in our set have known game-theoretic ground-truth outcome values assuming optimal play from both players. We describe how we filter which games of the 121 are estimatable via a game-theoretic optimal payoff. We first include those with known game-theoretic optimal payoffs (either definite player 1 win, definite draw, or definite player 2 win) according to ref. ^61^. Then, to approximate a larger set of games’ optimal payoffs, we additionally include games for which MCTS converged to {−1, 0, 1} in its payoff predictions (noting that MCTS estimates are not guaranteed to be perfect). This process results in a set of 78 out of the full 121 games for which we have estimated game-theoretic payoffs.

### Analysis methods

We next provide additional details on experimental analyses.

#### Comparing payoff predictions

Participants answered two questions in the game outcome reasoning experiment: how likely the first player wins given the game does not end in a draw (*P*(P1 wins∣not draw)), and how likely the game is to end in a draw (*P*(draw)). Together, these responses yield all information we need to compute an expected payoff. We compare the expected payoff from participants against those of models and report the *R*^2^. Specifically, we bootstrap subsample with replacement from the human participant samples, per game, and compare models against the mean payoff per sample. We also bootstrap subsample *k* for 20 simulated participants per model from the pool of model simulations. Unless otherwise stated, we run 1,000 bootstrap samples.

#### Sample complexity analyses

We select *k* (the number of simulations sampled for each simulated participant) by inspecting the variance of the payoff predictions over the simulated set of 20 participants against the empirical variance observed in the human data. We measure the root mean squared error between the model- and human-predicted variance for each game, as well as the Wasserstein distance between the distribution over model and human variances (Fig. 3c). We compute the latter to better capture the poor match of high *k* to people’s variances (that is, with increased *k*, variance collapses to zero). We find that that generally approximately *k* = 5–7 full game simulations reasonably well-captures human variance (Fig. 3c and Extended Data Fig. 1) under both measures. We report all main results with *k* = 6.

#### Fitting regression models to funness judgements

We fit a linear regression model to these features using the lmer package in R. Features are normalized to zero-mean and unit standard deviation. We fit the models to 1,000 bootstrapped subsamples of the human data for all games. Models are tasked with predicting the mean of participants’ funness judgements for each game. We compare models via an ANOVA test and AIC in Supplementary Information section 5.7.

In addition, we ran a generalization test wherein we fit regression models on 50% (59) of the 118 games studied (infinite boards were removed) and tested each model on the held-out 50% of games. We report both results in Extended Data Fig. 4 and include additional details in Supplementary Information section 5.7.

#### Assessing contribution of non-simulation-based features to funness judgements

In exploratory analyses, we assess the potential role of non-simulation-based features that can be read off of the game description alone. We compute a series of binary game traits that capture ways in which a game may differ from the base tic-tac-toe. For instance, a game may not be a 3 × 3 board; the game may end with *K* ≠ 3 pieces in a row to win; the second player may have a different win condition than the first player. We consider the following binary features: if the game has constrained win conditions (for example, no diagonals allowed); if the game has asymmetric win conditions (between players 1 and 2); if the game has asymmetric play dynamics (for example, player 2 can place two pieces on their first turn); if the board is not square; *K* ≠ 3 pieces in a row to win; if the board is larger than 3 × 3; if the game ends when the first player to achieve *K* in a row loses (that is, misère variants). We combine the binary features into a single aggregated measure of ‘approximate novelty’, which is the sum of the number of binary features present in any one game. We also explore the incorporation of board size (encoded as the number of rows × number of columns) as part of non-simulation-based game features that reasoners may consider when assessing the funness of games. We assess how well participants’ funness judgements can be explained by these features alone by fitting bootstrapped sets of linear regression models to these features in the same 50/50 train/test splits over the games. In addition, we explore the relative benefit of adding all binary features or any of the aggregate features to the simulation-based model over the full set of games (see Supplementary Information section 5.7 for more details and additional analyses).

#### Assessing game-time action selections

To assess how well models capture how people play, we condition the model in question on an intermediate board state of the actual human-versus-human game. All moves in the game are considered (encompassing early-, mid- and late-stage play). We computed the model’s predicted distribution over next moves. We assess the fit of this predicted distribution in two ways. First, we compute the log likelihood of the actual players’ moves against those predicted by the model. To handle cases where any model may place at or near-zero probability on any given position, thereby skewing the log likelihood, we incorporate a ‘slop’ parameter *α* that captures the probability that a player makes a move from the primary model (1 − *α* that they make a random move on that turn^62^). We sweep over *α* between 0.5 and 0.95 in increments of 0.05and compute the average log likelihood for each model over the settings of *α*. We show per-game stage and per-game results in Supplementary Information section 6. We conduct a one-sided paired *t*-test over each individual move as well as the aggregated move likelihood per game, comparing the Intuitive Gamer with the Expert Gamer and Intuitive Gamer with the Random Gamer, respectively.

#### Fitting admixture models to actions

We then consider two different admixture models: one over all moves made for a game, and one over all moves made for each player. Admixture models captures the notion that a player may play according to either the Intuitive Gamer, Expert Gamer or Random Gamer model on any turn. We jointly fit the weights of the Intuitive Gamer and Expert Gamer models and take the weight of the Random Gamer model to be the remaining mass that brings the weights to 1, over bootstrapped samples over the entire population of players. As the weights are fit jointly across the models, we need to be particularly careful about the magnitude of the results for the respective models. To that end, we jointly sweep over the temperature used in the action selection (with temperature ranging from 0.5 to 3.0 to produce a distribution over weights). We also fit the admixture over each individual, estimating the relative contribution of each model to the way they play. As each player plays only six games (and one round per game), we fit these weights to all of their moves across all games. We use scikit-learn for fitting, with the SLSQP optimizer.

#### Computing aggregate human distribution over predicted next action

For our ‘watch-and-predict’ experiments, we have access to finer-grained predictions from any one participant. We again extract distributions over likely next moves, conditioned on the observed intermediate board state from the novice and alternative models; however, we now compare the distributions directly with the suggested distribution of moves made by participants. We construct an aggregate move distribution over the participants, treating each of the five clicks per participant as contributing some mass to the ‘aggregate human’ distribution.

#### Comparing model- with human-predicted distributions over next actions

We compare distributions using the TVD; we fix temperature at 1 and sweep over *α* for both models and people. We conduct a similar one-sided paired *t*-test over the TVD across the three game stage boards seen per match and compare the Intuitive Gamer and Expert Gamer, and the Intuitive Gamer and Random Gamer. We compute split-half TVDs per stage, per match, per game by randomly splitting the participants’ distributions who saw that board, averaging those boards into two aggregate distributions and computing TVD between them. This forms our split-half human estimate, which we use to normalize the other models’ TVD. We demonstrate the robustness of our results to our choice of distributional measure by repeating the analyses with the Jensen–Shannon divergence in Supplementary Information section 6.2. We additionally run an admixture model over both participant- and game-level predicted watch distributions. These are optimized against the Jensen–Shannon divergence.

#### Comparing model- with human-predicted distributions over next actions to people’s played actions

We secondarily assess how well the distribution over suggested moves made by the watcher captures how people actually played by treating the suggested distribution of clicks by the human watcher akin to the move distributions from the models. We again assess the likelihood of the move played by the active player to the moves suggested by the predictors, the Intuitive Gamer model, and alternative models by sweeping over and averaging out a slippage parameter (*α*). We conduct these analyses in aggregate (over all board stages, matches and games), as well as at a per game level (over board stages and matches).

#### Modelling draw requests

We take initial steps to analyse evaluations made during the game by looking at players’ decisions of whether to accept or reject a draw, when offered. Draw requests could take place at any point during the game. For each board where a draw request was made, we run 40 simulations to the end of the game under the Intuitive Gamer agent model. We compute the expected payoff over bootstrapped subsampled sets of *k* = 6 outcomes (simulating a single player; previously computed under one of our *ψ*(*G*) game reasoning queries), as well as the expected remaining length of the game *ℓ* from state *s*_*t*_. We compute expected payoff here with respect to the player who is deciding whether or not to accept the draw request. Players receive a bonus payout of US$0.50 only if they win (nothing if they lose or draw); we can then compute an expected value that is the payoff ×0.5. For each set of bonus-adjusted expected payoffs and expected match length remaining, we fit a logistic regression model to predict whether a player would accept or reject the draw request, based on expected payoff and length, as well as the average predicted game funness predicted by people in the ‘just think’ game reasoning experiment. We fit the logistic regression model using the glm R package. We also explore the same computations under different agent models varying in depth (for example, the Expert Gamer model) and goal-directedness of the value function (for example, ablating the goal progress component).

We show the decision boundaries in Extended Data Fig. 8a and report the bootstrapped parameter fits in Extended Data Fig. 8b. For plotting, we use a collapsed measure of expected value which we call the ‘expected value of continuing’ (*C*) from state *s*_*t*_:

6 $$C({s}_{t})=(\psi (G)\times 0.5)-({\beta }_{{\rm{l}}{\rm{e}}{\rm{n}}{\rm{g}}{\rm{t}}{\rm{h}}}/{\beta }_{{\rm{u}}{\rm{t}}{\rm{i}}{\rm{l}}{\rm{i}}{\rm{t}}{\rm{y}}})\cdot {\ell }.$$

This measure combines the expected value (bonus-adjusted payoff) with the opportunity cost of continuing (based on expected length remaining).

### Reporting summary

Further information on research design is available in the Nature Portfolio Reporting Summary linked to this article.

## Online content

Any methods, additional references, Nature Portfolio reporting summaries, source data, extended data, supplementary information, acknowledgements, peer review information; details of author contributions and competing interests; and statements of data and code availability are available at 10.1038/s41586-026-10722-1.

## Supplementary information

Supplementary Information Supplementary Information, containing the following sections: 1. Summary of experimental variations on full intuitive gamer model; 2. Model pseudocode; 3. Additional model details; 4. Example experimental interfaces; 5. Additional analyses into human and model game evaluation; 6. Additional analyses into human and model action selection and prediction; 7. Exploratory analyses with a intermediate depth model; and 8. Game list.

Reporting Summary

Supplementary Data Data files associated with the main figures and extended data figures.

Peer Review File

## Figures and Tables

**Extended Data Table 1:**
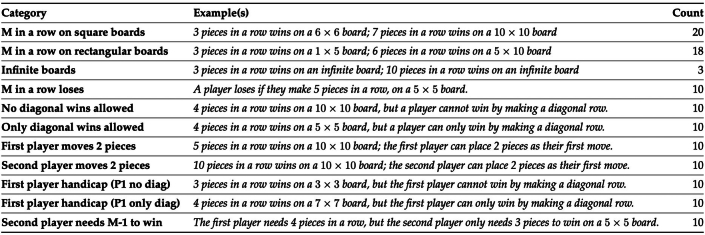
Diverse set of novel games

Wide variety of 121 two-player grid-based strategy games designed to study how people play and think about games when they are novices. Games vary in the board shape (e.g., square versus rectangular), win conditions (e.g., first to *K* in a row wins versus loses), and game dynamics (e.g., one player can play two pieces on their turn). Counts of games in each category, as well as one representative example game description for each.

**Extended Data Table 2:**
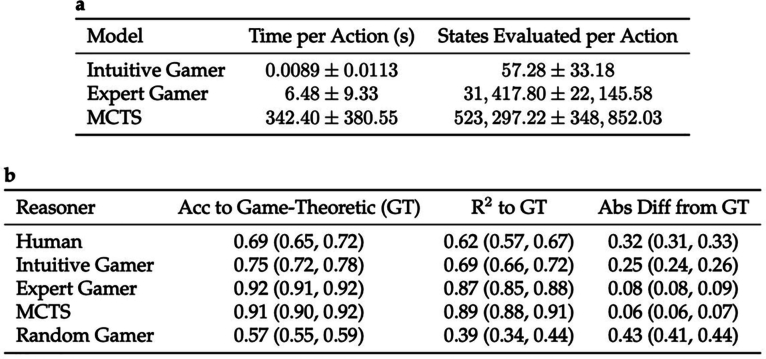
Resource-rational game reasoning, comparing compute efficiency and similarity of payoff estimates to estimates of game-theoretic optimal payoff

**a**, The Intuitive Gamer is more efficient than more sophisticated game reasoners, both in terms of average time to evaluate before deciding what action to select and average number of game states to evaluate before deciding what action to select. represent mean ± standard deviation over all moves when simulating evaluations for the game reasoning payoff predictions, for a subset of 41 of the 121 games. Additional details on cost comparisons are included in Supplementary Information 3. **b**, Human and model judgments are compared to the 78 of the 121 games where the game-theoretic optimal payoff is estimatable. Accuracy between predicted payoff and the approximate game-theoretic optimal is computed by taking the predicted payoff as “correct” if the game reasoner predicted a payoff  > 0.5 and the expected payoff is 1; correct if the predicted payoff is  < − 0.5 and the expected payoff is  − 1; correct if the predicted payoff is between  − 0.5 and 0.5 and the game is expected to end in a draw. *R*^2^ correlation is computed between the raw predicted payoffs and the game-theoretic optimal values, as well as the average absolute difference between the expected predicted payoff and approximate game-theoretic payoff (lower is closer to “correct”). Bootstrap 95% confidence intervals (CIs) are shown in parentheses, where bootstraps are over bootstrapped samples of participants (with replacement) for people or model simulations.

## Data Availability

Anonymized human data game evaluation, action selection and action prediction data are hosted via GitHub at github.com/collinskatie/intuitive-game-reasoning.git.

## Code availability

Code is available via GitHub at github.com/collinskatie/intuitive-game-reasoning.git.
