# Supplementary material for: People use fast and flat simulation to reason about new games
Source: Nature. 2026 Jul 15;655(8123):598–607. doi: 10.1038/s41586-026-10722-1 (PMC13372689; doi:10.1038/s41586-026-10722-1)
Supplement: Supplementary file 1 — Supplementary Information, containing the following sections: 1. Summary of experimental variations on full intuitive gamer model; 2. Model pseudocode; 3. Additional model details; 4. Example experimental interfaces; 5. Additional analyses into human and model game evaluation; 6. Additional analyses into human and model action selection and prediction; 7. Exploratory analyses with a intermediate depth model; and 8. Game list. [file 41586_2026_10722_MOESM1_ESM.pdf]

nature portfolio

https://doi.org/10.1038/s41586-026-10722-1

Supplementary information

People use fast and flat simulation to reason about new games

In the format provided by the authors and unedited

Supplementary Information

1 Summary of experimental variations on full Intuitive Gamer model **34**

2 Model pseudocode **34**
2.1 Intuitive Gamer reasoning module 34
2.2 Intuitive Gamer player module 35
2.3 Expert Gamer player module 35
2.4 Monte Carlo Tree Search 37

3 Additional model details **38**
3.1 Computational cost of models 38
3.2 Heuristic quality of Expert Gamer model 39
3.3 Intuitive Gamer parameters 39

4 Example experimental interfaces **43**

5 Additional analyses into human and model game evaluation **43**
5.1 Participant scratchpad usage 43
5.2 Participant experience 43
5.3 Simulated game length 44
5.4 Decomposed game outcome prediction tasks 47
5.5 Predicting payoff from non-simulation based linguistic features 48
5.6 Language model payoff and game-theoretic optimal comparisons 49
5.6.1 Language model prompting 50
5.7 Reasoning about game funness 50
5.7.1 Component ablations 50
5.7.2 Linguistic features 51

6 Additional analyses into human and model action selection and prediction **52**
6.1 Action selection accuracy and rank 52
6.2 Robustness to choice of distributional measure 53
6.3 Modeling choices with a softmax-based model 53
6.4 Predicted probability of played move relative to human predictions 55
6.5 Additional human- and model-predicted distributions 55
6.6 Game lengths 56
6.7 Draw requests and surrenders 56
6.8 Evaluating games after a single exposure 56
6.8.1 Evaluating games after one round of play 64
6.8.2 Evaluating games after one round of watching 66

7 Exploratory analyses with a intermediate depth model **66**
7.1 Model definition 67
7.2 Results 67

8 Game list **68**

1 Summary of experimental variations on full Intuitive Gamer model

We next summarize how the experiments varying model components show the necessity of all the different aspects of the Intuitive Gamer model (that it is fast, flat, goal-directed, and probabilistic).

That simulations are flat: by "flat," we mean the Intuitive Gamer player module is depth-limited. Our comparisons to the Expert Gamer (which is approximately depth-5) in Figure 3a, as well as our comparisons to a more intermediate depth gameplayer (depth-3) in Figure 3b and Supplementary Information 52 show distinctly lower R^2 relative to human payoff predictions. We also find that computing the funness model features under a deeper gameplay agent (Extended Data Figure 4) does not capture human funness judgements as well as a depth-1 Intuitive Gamer agent. Similarly, we find that a depth-limited player module better captures people's prediction actions in-game and their judgments about others' likely play (see Figure 5, comparison between Intuitive Gamer and Expert Gamer).

That simulations are fast: by "fast", we mean that the Intuitive Gamer's reasoning is fast. This is in part because simulations are shallow, of course, but also because only a small number of simulations need to be run to make each judgment at the level of resolution or confidence we see in people's judgments. The model's judgments are always stochastic, and by decreasing or increasing the number of simulations that each run of the reasoning module conducts per trial, we can obtain more or less variable judgments about game fairness and payoff over different runs of the model. By comparing the variance in judgments across Intuitive Gamer model runs (each run simulating a single human participant) with the variance in judgments across a sample of actual human participants, and equating for number of actual and simulated participants, we can estimate the effective number of simulations people are running (to the extent they are well modeled by an Intuitive Gamer model). We find that using only around $5 - 7$ simulations from the Intuitive Gamer reasoning module best captures the variability in human judgments, and show this result in Figure 3c and Extended Data Figure 3.

That simulations are goal-directed: by "goal-directed," we mean that the Intuitive Gamer player module chooses actions in a motivated fashion, towards its own goal—and assumes the other player is also motivated to meet their goal (and hence, the Intuitive Gamer tries to block the opponent's progress). Ablating either or both of these two features—progress towards one's own goal — is necessary to capture people's payoff judgments (see Figure 3b), as well as their actual in-game moves and suggested moves for others (Extended Data Figure 9). Goal-directedness is also critical for capturing people's decisions about whether or not to accept a draw request (Extended Data Figure 10).

That simulations are probabilistic: by "probabilistic," we mean that actions are selected by the Boltzmann rule, sampled from a softmax distribution over the estimated value of actions (based on the above goal-directed heuristic). We also consider a deterministic action selection policy (that effectively takes the best action on each move), which we find neither qualitatively nor quantitatively captures the variance in people's payoff predictions (see Figure 3a and 3b). We conduct a wider range of temperature analyses below.

2 Model pseudocode

We include pseudocode for our primary and alternate models.

2.1 Intuitive Gamer reasoning module

We provide example pseudocode for the reasoning module applied to estimating the payoff of matches. Critically, the reasoning module simulates k games; based on these game playouts

various queries could be computed (e.g., estimating the payoff of the game, or other properties like the length of the match).

Algorithm 1 Reasoning Module

```
k ← number of simulated matches
outcomes ← []
for i = 1 to k do
    o ← PLAYERMODULE                          ▷ Simulate one match (self-play)
    outcomes.append(o)                        ▷ Store outcome
end for
return PAYOFF(outcomes)                       ▷ Estimate expected payoff from outcomes
```

2.2 Intuitive Gamer player module

We next provide pseudocode on the player modules.

Algorithm 2 Intuitive Gamer: MakeMove(s)

- n_1 ←length of longest *live* connection extended
- **if** n_1 is winning length **then**
  - $n_1 = n_1 + 1$
- **end if**
- n_2 ←length of longest *live* opponent connection blocked $- 0.5$
- **if** n_2 is winning length **then**
  - $n_2 = n_2 + 0.5$
- **end if**
- ϵ ← Euclidean distance to center of board, normalized so $d = 1$ for the corner pieces
- $\tilde{\mathcal{V}}(a, s) = 2^{(1-\epsilon(a))} + 2^{n_1(a)} + 2^{n_2(a)}$
- **return** Sample(Softmax($\{a : \tilde{\mathcal{V}}(a, s)$ for $a \in s.actions\}$))

As explained in our main text, our Intuitive Gamer player module is flat, goal-directed, and probabilistic. It incorporates shallow search and considers only a constrained set of heuristics ($\tilde{\mathcal{V}}$) for measuring intermediate game states. Actions are evaluated based on their proximity to the center of the board d, the length of the live connections they extend n_1, and the length of the opponent's live connections that they block. By *live connections* we mean connected pieces that have enough open positions on the corresponding direction such that they can be extended to form a winning configuration. Actions are then sampled from a softmax distribution over open-board states, weighted by the heuristic value computations.

2.3 Expert Gamer player module

We provide pseudocode for the Expert Gamer model (designed to generalize the 4-in-a-row model of human gameplay from (74)). Its algorithm repeats three sub-procedures, SelectNode, ExpandNode, and Backpropagate, within the main procedure MakeMove, which ultimately makes a move by sampling from a softmax distribution.

The function Node is the constructor for the nodes of the tree search. As described in the "Methods" section, in our simulations we repeat the procedure in MakeMove for $num\_steps = 636$ iterations, drawing on results from an empirical parameter fit in (74) which found that expert human players (playing a specific 4-in-a-row variant) were well modeled by a heuristic search procedure whose number of iterations is geometrically distributed with a mean stopping parameter of $1/636$. Our model does not use a stochastic stopping parameter and just runs

Algorithm 3 Expert Gamer: MakeMove

```
root ← Node(s)
for i = 1 to num_steps do
    n ← SelectNode(root)
    ExpandNode(n)
    Backpropagate(n, root)
end for
return Sample(softmax({c : c.val for c ∈ root.children}))
```

deterministically to $num\_steps = 636$ iterations for all simulations. In the following, *player_type* $\in \{X, O\}$ and *move_number* is the index of the current move over the entire game (to account for variants which specify that a given player goes twice as their opening move).

Algorithm 4 Expert Gamer: SelectNode(n)

```
root ← n
while n.children ≠ ∅ do
    if rand()< ϵ then
        n = unirandom({c : c.val for c ∈ n.children})
    else
        if n.player_type = root.player_type then
            n = rand-argmax({c : c.val for c ∈ n.children})
        else
            n = rand-argmin({c : −c.val for c ∈ n.children})
        end if
    end if
end while
return n
```

Algorithm 5 Expert Gamer: ExpandNode(n)

```
s ← n.state
for all m ∈ LegalMoves(s) do
    b ← PlacePiece(s, m, n.player_type)
    i ← n.move_number
    n.children.append(Node(b, i + 1, Ṽ^EG(s)))
end for
```

Within the procedure ExpandNode, the function $\tilde{\mathcal{V}}^{\text{EG}}$ is an extension of our Intuitive Gamer heuristic function $\tilde{\mathcal{V}}$ defined above, and it represents a more sophisticated heuristic. To set a frame of reference, suppose player X is evaluating the utility of board state b. The heuristic $\tilde{\mathcal{V}}^{\text{EG}}$**(b)** is evaluated as

$$\tilde{\mathcal{V}}^{\text{EG}}(s) = \sum_i (2 \times \mathbb{I}\{\text{the } i\text{'th move is done by } X\} - 1)\tilde{\mathcal{V}}(p_i, s_i). \tag{7}$$

where b_i is the i'th board observed in the game and p_i is the i'th move played in the game. The value function is defined symmetrically when O acts. We make this modification so that $\tilde{\mathcal{V}}^{\text{EG}}$ incorporates information from all potential future actions on the board, unlike $\tilde{\mathcal{V}}$ which only incorporates local information about an action-state pair (p, b).

Algorithm 6 Expert Gamer: Backpropagate$(n, root)$

if $n.player\_type = root.player\_type$ **then**
  $n.val \leftarrow \max_{c \in n.children} c.val$
else
  $n.val \leftarrow \min_{c \in n.children} c.val$
end if

2.4 Monte Carlo Tree Search

Similar to the "Expert Gamer" model, Monte Carlo Tree Search (MCTS) repeats four sub-procedures: SelectNode, ExpandNode, DepthCharge and Backpropagate, but the SelectNode, ExpandNode, and Backpropagate sub-procedures are different from their corresponding counterparts in the human expert model. We run MCTS for $10,000$ steps, which we find balances a close approximation of the optimal for many games while keeping the runtime of any single game trial to less than two days.

Algorithm 7 MCTS: MakeMove

$root \leftarrow$ Node(s)
for $i = 1$ to num_steps **do**
  $n \leftarrow$ SelectNode$(root)$
  ExpandNode(n)
  $game\_outcome =$ DepthCharge$(n, root)$
  Backpropagate$(n, root, game_outcome)$
end for
return $\arg\max_{c \in root.children} c.val$

Algorithm 8 MCTS: SelectNode(n)

while $n.children \neq \emptyset$ **do**
  $n = \arg\max_{c \in n.children} c.UCB$
end while
return n

To balance exploring and exploiting in the MCTS SelectNode sub-procedure, we use a standard upper confidence bound (UCB) (75).

$$c.UCB = \arg\max_{c \in root.children} \left(\frac{c.val}{c.visits} + \sqrt{2 \frac{\log(c.parent.visits)}{c.visits}} \right).$$

Algorithm 9 MCTS: ExpandNode$(n, root)$

$s \leftarrow n.state$
for all $m \in$ LegalMoves(s) **do**
  $b \leftarrow$ PlacePiece$(s, m, n.player_type)$
  $i \leftarrow n.move\_number$
  $n.children.append($Node$(b, i + 1))$
end for

Finally, as noted above, our implementations of the Expert Gamer and MCTS algorithms always parameterize simulation with a formal specification of the rules of any given game

Algorithm 10 MCTS: Backpropagate($n, root, game\_outcome$)

```
n.visits ← n.visits + 1
if n.parent then
    if n.parent.player_type == root.player_type then
        n.val ← n.val + game_outcome
    else
        n.val ← n.val + (1 − game_outcome)
    end if
    Backpropagate(n.parent, root, game_outcome)
end if
```

Algorithm 11 MCTS: DepthCharge(n)

```
s ← n.state
i ← n.move_number
while not HasTerminated(s) do
    player_type ← n.player_sequence[i]
    m ← RandMove(s)
    i ← i + 1
    s ← PlacePiece(s, m, player_type)
end while
if IsWin(s, root.player_type; game_rules) then
    return 1
else if IsDraw(s; game_rules) then
    return 1/2
else
    return 0
end if
```

(e.g., restrictions on whether a given player can win only in certain directions on the board). We implement $\tilde{\mathcal{V}}$ and DepthCharge as described in the main text so that intermediate value functions and win conditions are calculated with respect to the specific game dynamics and win conditions for each game variant. *MakeMove* similarly takes in the current *move_number* move index to account for games in which player order is not strictly alternating, such as games in which the first or second player goes twice as their opening move.

3 Additional model details

We report additional details on the models, including compute usage, model-model simulations, and other design choices informing the Expert Gamer model[†].

3.1 Computational cost of models

We assess the relative compute demands of different gameplay models on two measures: (1) the time it takes each model to select an action and (2) how many board states have their value assessed in the process of the model deciding what action to take. We measure the first metric as the average time per move counts the time, in seconds, that each model takes selecting an action of where to move. This quantity is then averaged across all games, game trials, and actions

[†] One of the 121 games did not complete and save for the infinite board where 10 in a row is needed to win; we filter out this game from the "just think" game fairness comparisons for the Expert Gamer model. Infinite boards are not used in the funness, play, nor watch and predict experiments.

within a game. Table 2 shows the average wall-clock time per move for the three models broken down by game. We note that wall-clock time is potentially limited by the exact implementation and hardware used. To reduce some of these confounds, all models utilize a shared codebase and are run on the same hardware. We measure the second quantity by counting the number of nodes in the constructed tree search for each move. This quantity is then averaged across game trials and moves within a game trial. Table 1 shows the average number of nodes whose heuristic values are evaluated in the search tree for each move for the Intuitive Gamer, Expert Gamer, and MCTS models. Each row in the table corresponds to one of the 41 games used in the "human-human play" experiment. All game simulations involve self-play with that same model.

3.2 Heuristic quality of Expert Gamer model

The Expert Gamer model is more sophisticated than the Intuitive Gamer across two axes—search depth and heuristic sophistication. While it is easy to verify that the Expert Gamer model has a deeper search depth than the Intuitive Gamer, it is difficult to compare the heuristic sophistication of the two models without knowing the objective value of each board state of each game. To this end, we simulate game trials of the Expert Gamer model against a faithful implementation of the Intuitive Gamer model that incorporates search depth. We observe that the Expert Gamer model dominates regardless of whether or not it plays as Player 1 or 2. Our results are reported in Table 3. The Ablated Expert removes the mechanism that incorporates features of the global board state instead of the purely local features. In particular, the value function of the ablated expert differs from the value function of our expert model as given by Equation 7. The value function of the ablated expert is given by:

$$\mathcal{V}^{\text{EG, abl}}(s') = (2 \times \mathbb{I}\{\text{the } i\text{'th move is } X\} - 1)\mathcal{V}(a, s), \tag{8}$$

where s is the board state that immediately preceded s' on the game tree and a is the action that takes board state a to state s'. The value function of the ablated expert is clearly very myopic as it depends only on the most recent action of the board state.

We incorporate an exploration component to our Expert Gamer model by setting a probability ϵ with which we explore a uniformly random child node. We have the Expert Gamer model play against the Intuitive Gamer (flat and depth-3 variants, as described at the end of the Supplement) on the subset of 41 games used in the play experiment with different values of ϵ' and set ϵ to be the ϵ' that yields the greatest expected advantage over the two models. Results are reported in Table 4, which shows the expected payoff averaged across the expert's opponent. Choice of ϵ has a minimal impact (Table 4); still, we take the maximum shows, this value turns out to be $\epsilon = 0.0001$.

3.3 Intuitive Gamer parameters

We additionally assess the sensitivity of the Intuitive Gamer model to choice of parameters (w) for the heuristic value function ($\tilde{\mathcal{V}}$). We fix the temperature $\tau = 1$ and vary w_{connect}, w_{block}, and w_{center}. We sweep over w_{connect}, w_{block}, and w_{center} between 0 and 2 in steps of 0.2 respectively, and run 50 simulations of our model for each setting (which we bootstrap subsample from 100 times, simulating 20 simulated participants running $k = 6$ mental simulations each). We then compute the R^2 under each variants' predictions relative to the human predicted payoffs. We observe in Figure 17 higher sensitivity to the parameterization of the goal-progress and goal-blocking components, highlight the importance of "goal-directedness" in the Intuitive Gamer model and appropriately balancing offense and defense. The sweeps also underscore that our selection of simply weighting all values with 1 is a reasonable modeling choice.

Game	Intuitive Gamer	Expert Gamer	MCTS
3x3 3 P1/2 P2	7.71 ± 1.09	340.79 ± 421.19	$6,946.28 \pm 7,373.94$
3x3 3	6.72 ± 2.00	489.21 ± 477.21	$11,769.89 \pm 13,908.48$
3x3 3 (P2 2p)	6.41 ± 2.23	$923.50 \pm 1,062.07$	$12,168.06 \pm 13,930.55$
3x3 3 (P1 2p)	6.73 ± 1.99	152.83 ± 73.01	$13,445.12 \pm 14,038.39$
3x3 3 L	6.46 ± 2.17	380.69 ± 547.15	$13,618.89 \pm 15,778.72$
2x5 3	7.04 ± 2.43	218.78 ± 172.61	$18,019.79 \pm 18,641.68$
1x10 3	6.93 ± 2.50	259.58 ± 219.57	$18,860.72 \pm 19,800.13$
4x4 3 L	11.63 ± 3.43	$3,618.14 \pm 2,540.70$	$62,150.06 \pm 44,828.95$
4x4 3 D	11.45 ± 3.70	$3,367.23 \pm 2,507.85$	$64,164.73 \pm 41,121.09$
4x4 3 HV	12.87 ± 2.90	$3,171.31 \pm 3,045.33$	$74,368.59 \pm 41,785.08$
5x5 2	24.02 ± 0.81	606.18 ± 470.70	$75,410.55 \pm 56,977.89$
5x5 3 L	18.23 ± 5.03	$7,547.74 \pm 3,772.27$	$124,690.63 \pm 71,792.33$
4x6 5	16.41 ± 5.70	$6,569.44 \pm 3,984.28$	$127,823.20 \pm 62,653.84$
4x6 4	16.75 ± 5.56	$6,310.25 \pm 4,502.73$	$129,860.14 \pm 64,085.15$
5x5 4 D	17.20 ± 5.83	$7,599.20 \pm 4,259.91$	$134,135.65 \pm 64,384.30$
5x5 4 HV	17.93 ± 5.46	$6,464.76 \pm 4,240.71$	$136,459.28 \pm 64,148.43$
5x5 4 (P2 2p)	17.89 ± 5.60	$7,103.19 \pm 5,034.45$	$136,702.71 \pm 64,837.41$
5x5 4	18.05 ± 5.36	$6,618.10 \pm 4,702.53$	$144,846.18 \pm 62,310.97$
5x5 3 (P1 HV)	22.57 ± 1.86	$6,168.12 \pm 6,553.56$	$153,006.93 \pm 71,992.53$
5x5 3 (P1 D)	22.18 ± 2.22	$6,355.83 \pm 6,316.61$	$153,930.07 \pm 71,405.38$
5x5 4 (P1 2p)	19.14 ± 4.72	$6,984.52 \pm 5,777.07$	$155,149.51 \pm 57,176.56$
5x5 3	22.54 ± 1.93	$6,167.83 \pm 6,553.92$	$157,519.28 \pm 71,077.72$
5x5 4 P1/3 P2	21.94 ± 2.29	$7,475.19 \pm 6,392.12$	$176,543.76 \pm 45,694.49$
3x10 3	27.25 ± 2.24	$7,697.46 \pm 7,894.15$	$204,352.35 \pm 82,146.78$
4x9 4	26.77 ± 7.70	$13,698.40 \pm 7,066.52$	$240,662.55 \pm 85,097.34$
7x7 4 L	33.72 ± 11.05	$17,258.99 \pm 7,808.52$	$301,968.11 \pm 119,135.63$
5x10 5	34.18 ± 11.61	$17,794.77 \pm 8,438.64$	$313,634.43 \pm 120,188.42$
7x7 4 HV	38.99 ± 9.33	$17,756.62 \pm 8,252.03$	$377,773.04 \pm 101,370.85$
7x7 4 D	36.39 ± 10.42	$18,122.45 \pm 8,075.41$	$378,955.54 \pm 111,335.85$
7x7 4 (P1 D)	42.87 ± 4.78	$20,734.93 \pm 10,316.27$	$387,077.75 \pm 89,789.16$
7x7 4	42.49 ± 5.05	$21,326.28 \pm 10,600.34$	$387,483.47 \pm 110,703.17$
7x7 4 (P1 HV)	41.06 ± 6.16	$19,509.22 \pm 11,271.34$	$387,637.26 \pm 100,578.80$
7x7 4 (P2 2p)	42.86 ± 5.22	$20,767.11 \pm 10,223.01$	$388,333.98 \pm 109,289.19$
7x7 4 (P1 2p)	43.00 ± 4.69	$17,274.72 \pm 12,929.50$	$397,684.27 \pm 92,618.19$
5x10 4	42.84 ± 5.23	$21,163.57 \pm 10,751.05$	$397,818.92 \pm 109,009.24$
7x7 4 P1/3 P2	45.42 ± 2.84	$16,165.24 \pm 13,332.40$	$399,809.50 \pm 91,272.35$
10x10 5 (P1 D)	82.90 ± 13.98	$44,812.99 \pm 16,198.57$	$865,140.51 \pm 103,573.52$
10x10 3	97.43 ± 1.99	$33,495.23 \pm 25,525.04$	$872,437.53 \pm 177,821.03$
10x10 5 (P1 HV)	77.77 ± 17.70	$44,346.05 \pm 16,328.17$	$874,752.68 \pm 111,721.08$
10x10 5	83.64 ± 15.43	$48,697.30 \pm 16,778.13$	$885,028.95 \pm 117,608.47$
10x10 4	92.99 ± 6.52	$43,112.32 \pm 24,438.41$	$897,954.52 \pm 136,325.31$

Table 1: Efficiency, measured by number of states evaluated, across game reasoning modules. Average game board states explored per move across all game configurations. Values represent mean ± standard deviation.

Game	Intuitive Gamer	Expert Gamer	MCTS
3x3 3 P1/2 P2	$4.11 \times 10^{-4} \pm 1.02 \times 10^{-4}$	0.05 ± 0.02	16.25 ± 4.00
3x3 3 (P1 2p)	$4.10 \times 10^{-4} \pm 1.46 \times 10^{-4}$	0.03 ± 0.01	17.61 ± 5.01
3x3 3	$4.25 \times 10^{-4} \pm 1.73 \times 10^{-4}$	0.08 ± 0.03	18.85 ± 6.30
3x3 3 (P2 2p)	$4.02 \times 10^{-4} \pm 1.47 \times 10^{-4}$	0.09 ± 0.05	19.00 ± 6.41
3x3 3 L	$4.08 \times 10^{-4} \pm 1.47 \times 10^{-4}$	0.08 ± 0.03	19.08 ± 6.07
2x5 3	$3.17 \times 10^{-4} \pm 1.36 \times 10^{-4}$	0.04 ± 0.02	19.13 ± 6.33
1x10 3	$3.38 \times 10^{-4} \pm 1.40 \times 10^{-4}$	0.05 ± 0.02	19.39 ± 6.11
4x4 3 HV	$4.37 \times 10^{-4} \pm 1.19 \times 10^{-4}$	0.21 ± 0.10	38.03 ± 6.55
4x4 3 L	$7.27 \times 10^{-4} \pm 2.67 \times 10^{-4}$	0.45 ± 0.20	38.34 ± 12.44
4x4 3 D	$5.54 \times 10^{-4} \pm 1.79 \times 10^{-4}$	0.22 ± 0.07	38.95 ± 11.64
5x5 2	$1.85 \times 10^{-3} \pm 4.62 \times 10^{-4}$	0.13 ± 0.03	51.45 ± 5.11
4x6 5	$1.11 \times 10^{-3} \pm 3.40 \times 10^{-4}$	0.39 ± 0.12	54.35 ± 16.23
5x5 4 HV	$7.20 \times 10^{-4} \pm 2.58 \times 10^{-4}$	0.46 ± 0.15	68.63 ± 19.58
5x5 4 D	$9.78 \times 10^{-4} \pm 3.28 \times 10^{-4}$	0.83 ± 0.27	70.84 ± 21.44
4x6 4	$1.17 \times 10^{-3} \pm 3.44 \times 10^{-4}$	0.61 ± 0.20	71.58 ± 21.57
5x5 3 L	$1.58 \times 10^{-3} \pm 4.87 \times 10^{-4}$	1.30 ± 0.55	72.12 ± 24.70
5x5 4	$1.55 \times 10^{-3} \pm 4.84 \times 10^{-4}$	0.84 ± 0.36	76.41 ± 21.90
5x5 4 (P2 2p)	$1.43 \times 10^{-3} \pm 4.42 \times 10^{-4}$	0.82 ± 0.25	77.91 ± 22.59
5x5 3 (P1 HV)	$1.18 \times 10^{-3} \pm 2.00 \times 10^{-4}$	0.60 ± 0.18	78.96 ± 12.05
5x5 3 (P1 D)	$1.51 \times 10^{-3} \pm 3.69 \times 10^{-4}$	0.71 ± 0.22	79.25 ± 15.16
5x5 3	$1.81 \times 10^{-3} \pm 4.57 \times 10^{-4}$	0.83 ± 0.27	80.16 ± 10.07
5x5 4 (P1 2p)	$1.45 \times 10^{-3} \pm 3.56 \times 10^{-4}$	0.74 ± 0.28	85.30 ± 19.61
3x10 3	$1.91 \times 10^{-3} \pm 4.44 \times 10^{-4}$	0.87 ± 0.22	89.06 ± 13.60
5x5 4 P1/3 P2	$1.73 \times 10^{-3} \pm 4.55 \times 10^{-4}$	0.84 ± 0.22	89.83 ± 13.46
4x9 4	$2.30 \times 10^{-3} \pm 6.36 \times 10^{-4}$	1.59 ± 0.33	129.93 ± 35.15
7x7 4 HV	$2.37 \times 10^{-3} \pm 6.76 \times 10^{-4}$	2.02 ± 0.46	189.08 ± 43.82
5x10 5	$3.81 \times 10^{-3} \pm 1.20 \times 10^{-3}$	2.73 ± 0.70	211.20 ± 68.08
7x7 4 L	$4.58 \times 10^{-3} \pm 1.42 \times 10^{-3}$	4.41 ± 1.25	231.47 ± 75.59
7x7 4 (P1 HV)	$4.02 \times 10^{-3} \pm 9.90 \times 10^{-4}$	3.00 ± 0.58	235.27 ± 46.61
7x7 4 P1/3 P2	$6.14 \times 10^{-3} \pm 1.62 \times 10^{-3}$	3.67 ± 0.65	239.62 ± 40.60
7x7 4 D	$3.24 \times 10^{-3} \pm 9.26 \times 10^{-4}$	3.56 ± 0.99	243.65 ± 47.14
7x7 4 (P1 D)	$4.75 \times 10^{-3} \pm 1.11 \times 10^{-3}$	3.68 ± 0.58	261.60 ± 49.34
7x7 4	$6.53 \times 10^{-3} \pm 2.22 \times 10^{-3}$	4.30 ± 0.56	261.65 ± 43.19
5x10 4	$5.74 \times 10^{-3} \pm 1.44 \times 10^{-3}$	3.98 ± 0.81	266.12 ± 35.31
7x7 4 (P2 2p)	$6.45 \times 10^{-3} \pm 1.57 \times 10^{-3}$	4.68 ± 0.92	270.42 ± 41.26
7x7 4 (P1 2p)	$6.29 \times 10^{-3} \pm 1.87 \times 10^{-3}$	3.65 ± 0.90	291.32 ± 46.20
10x10 3	$2.78 \times 10^{-2} \pm 6.36 \times 10^{-3}$	17.34 ± 2.94	862.42 ± 66.75
10x10 5 (P1 HV)	$1.49 \times 10^{-2} \pm 3.54 \times 10^{-3}$	15.06 ± 2.14	953.86 ± 263.76
10x10 5 (P1 D)	$1.97 \times 10^{-2} \pm 4.86 \times 10^{-3}$	17.58 ± 2.61	987.14 ± 265.40
10x10 4	$2.50 \times 10^{-2} \pm 5.11 \times 10^{-3}$	20.28 ± 4.42	1015.55 ± 124.69
10x10 5	$2.13 \times 10^{-2} \pm 3.28 \times 10^{-3}$	21.28 ± 2.43	1151.12 ± 169.73

Table 2: Efficiency, measured by wall-clock time per action, across game reasoning modules. Average time per move (seconds) across all game configurations. Values represent mean ± standard deviation. The values are sorted by the average time per move of the MCTS model.

Matchup	Wins (%)	Losses (%)	Draws (%)
Expert vs Ablated Expert	43.0	16.4	40.5
Ablated Expert vs Expert	19.3	41.4	39.3

Table 3: Comparing the Expert Gamer with inheritance in the value function against a lesioned variant. Game outcome percentages for Expert Gamer versus an Ablated Expert Gamer, which does not incorporate the entire global state matchups in is computation of the heuristic value of each state. Results are based on 40 match simulations over the subset of 41 games used in the play experiment, showing win, loss, and draw rates of Player 1 averaged over all simulations and games. Rows are formatted as Player 1 model vs. Player 2 model.

ϵ	Expected payoff for the Expert Gamer
0.0	0.0831 ± 0.863
1e-4	0.0845 ± 0.861
1e-3	0.0683 ± 0.866
1e-2	0.0651 ± 0.865
0.1	0.0328 ± 0.879
0.2	-0.00396 ± 0.887

Table 4: Selection of ϵ in the Expert Gamer based on payoff in cross-model gameplay. Expected payoff and standard deviation for the Expert Gamer model, averaged across opponent and whether the expert played first or second.

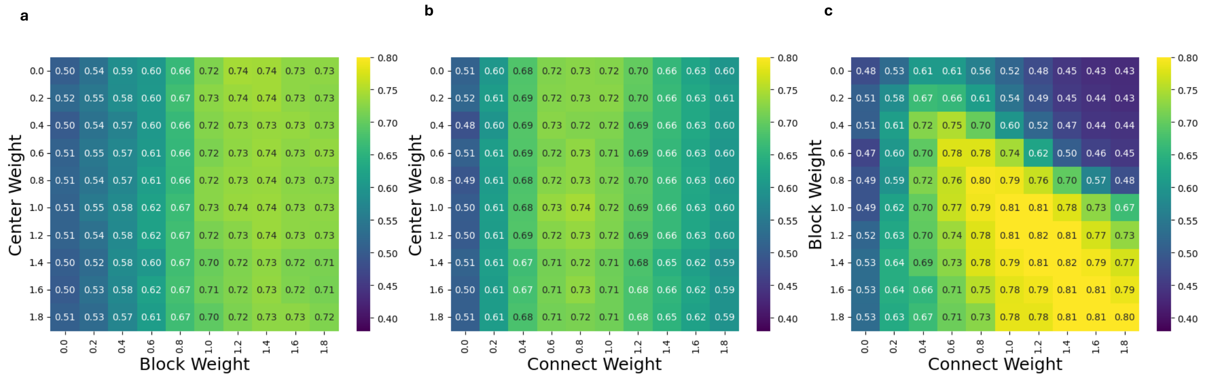

Figure 17: Parameter sensitivity analyses. Analyzing the impact of the goal progress (offensive "connect" component), goal blocking (defensive "block" component), and center weight on fit to people's judgments in the "just think" experiment, with subset of simulations for different settings of the heuristic value function weights. We run 100 bootstrapped subsamples of 20 participants from the "just think" experiment per game with $k = 6$, and compute R^2 between the average human payoff from the subset versus the average model payoff. Each cell averages over the held-out parameter (e.g., averaging over all settings of the connect weight in the leftmost plot). Fit to participants is substantially more impacted by the components controlling offense and defense, rather than center; interestingly, the defensive blocking component generally requires a value as high or more than the corresponding offensive component.

4 Example experimental interfaces

We include example interfaces for each of our main behavioral experiments. Figure 18 depicts example interfaces from the "just think" judging game outcome and game funness experiments. Figures 19 and 20 depict example interfaces from the human-human gameplay experiment. Figure 21 depicts examples from the indirect human watching and predicting play experiment.

5 Additional analyses into human and model game evaluation

5.1 Participant scratchpad usage

Before making their judgments, participants had the option to interact with an interactive version of the board to simulate self-play (a "scratchpad") to minimize demands on spatial working memory. Participants made on average 1.60 ± 1.46 SD rollouts for the payoff task and 1.36 ± 1.15 SD rollouts for the funness task. We define a rollout as the number of times a participant started interacting on a fresh board (i.e., zero rollouts indicates no interaction with the board; one rollout indicates on usage of the board; two indicates one restart of board clicks). We depict a histogram over rollouts per task in Figure 22. We do not draw a direct parallel between scratchpad rollouts and mental simulation (it is highly plausible that participants are doing more mental simulation than they are demonstrating on the scratchpad); we include the scratchpad to reduce the potential demands on spatial working memory of our task such that we can focus on studying reasoning. While from manual inspection, many participants using the scratchpads did appear to simulate play, some participants seemed to just click around without intention.

5.2 Participant experience

Participants were also asked at the end of the "just think" experiment how much prior experience they had with Tic-Tac-Toe, Connect-4, and Gomoku. Participants self-reported

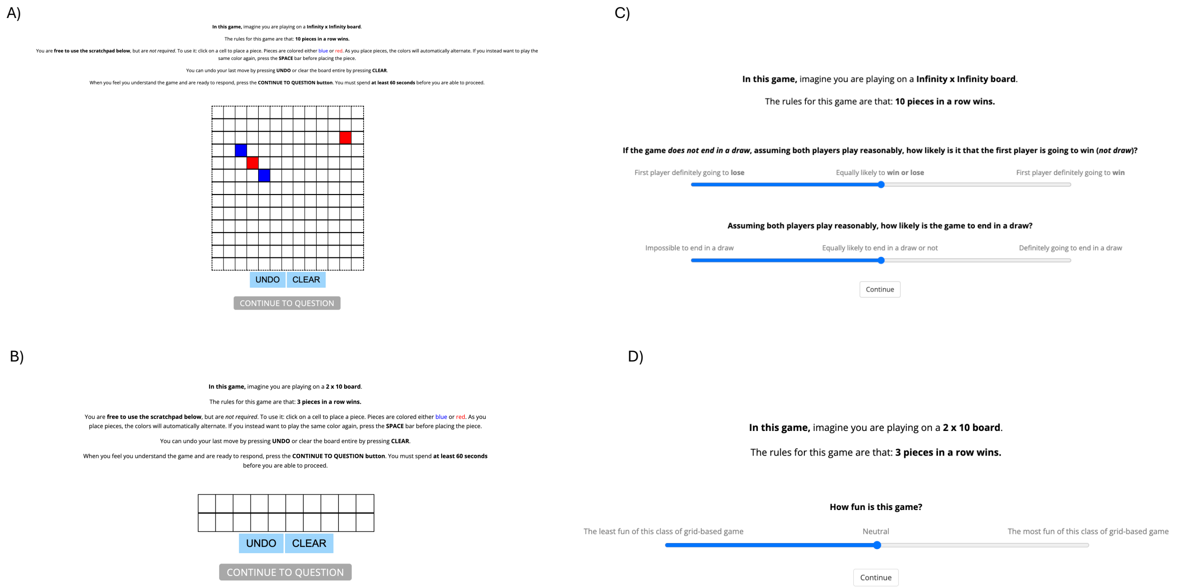

Figure 18: "Just think" evaluating games before any experience example interfaces. For each game, participants are given a scratchpad which they can optionally use while thinking about their response (**a-b**). Pieces will automatically change between blue and red upon each click, unless overridden (see Methods). Infinite boards **(a)** are shown with dashes along the edges. For each game, participants either judge game outcomes **(c)** or game funness **(d)** using sliders.

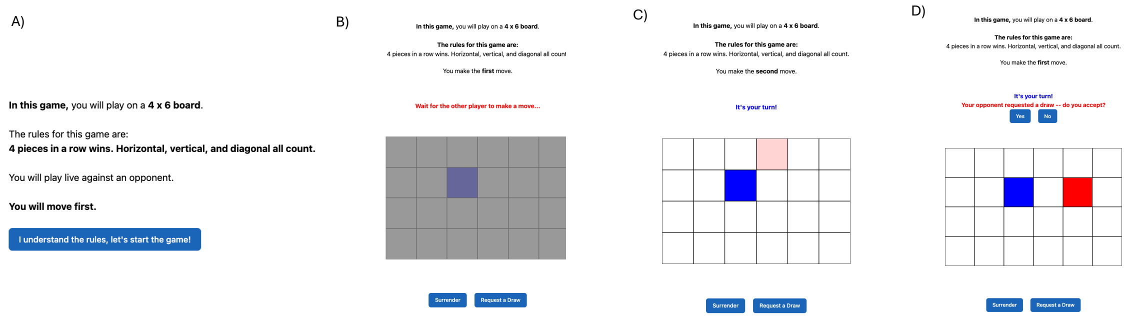

Figure 19: Example interfaces from the human-human gameplay study. a, Participants are first informed of the game rules and whether they will play first or second. **b,** The board is disabled and greyed-out when it is not their turn. **c,** When it is their turn, they see a hover (in the color of their piece) before they decide where to play. They decide to play by clicking on the board. **d,** When a player requests a draw, the other player is informed and allowed to accept or reject the draw.

their prior experience on three slider scales; one per game. Sliders ranged from 0 to 100, where 0 = "No prior experience," 50 = "Some prior experience playing", and 100 = "Substantial prior experience playing." Most participants self-reported experience with Tic-Tac-Toe; Experience with Connect-4 was more variable and few participants had any experience with Gomoku (see Figure 23). Future work can better investigate the interplay of game-specific experience and pre-play judgments.

5.3 Simulated game length

For the primary model, the Intuitive Gamer reasoning module simulates play to the end of the games. The median match length from the 121 set of games is 14.8 moves; approximately 75%

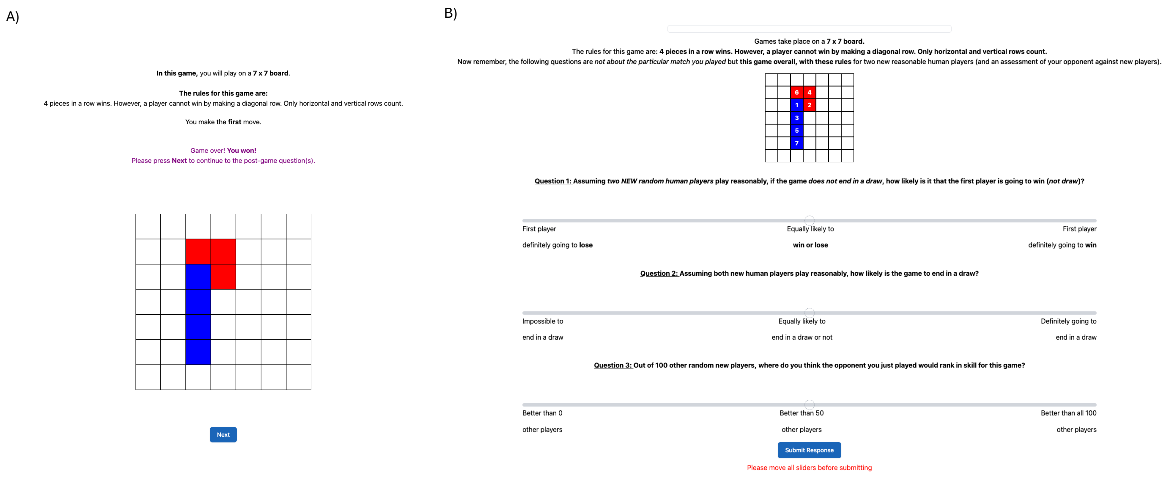

Figure 20: Additional example interfaces from the human-human gameplay study. a, After the game is over, both players are informed of the outcome. **b,** Each player then makes a series of judgments about the game via sliders and are shown a "snapshot" of how their match had unfolded.

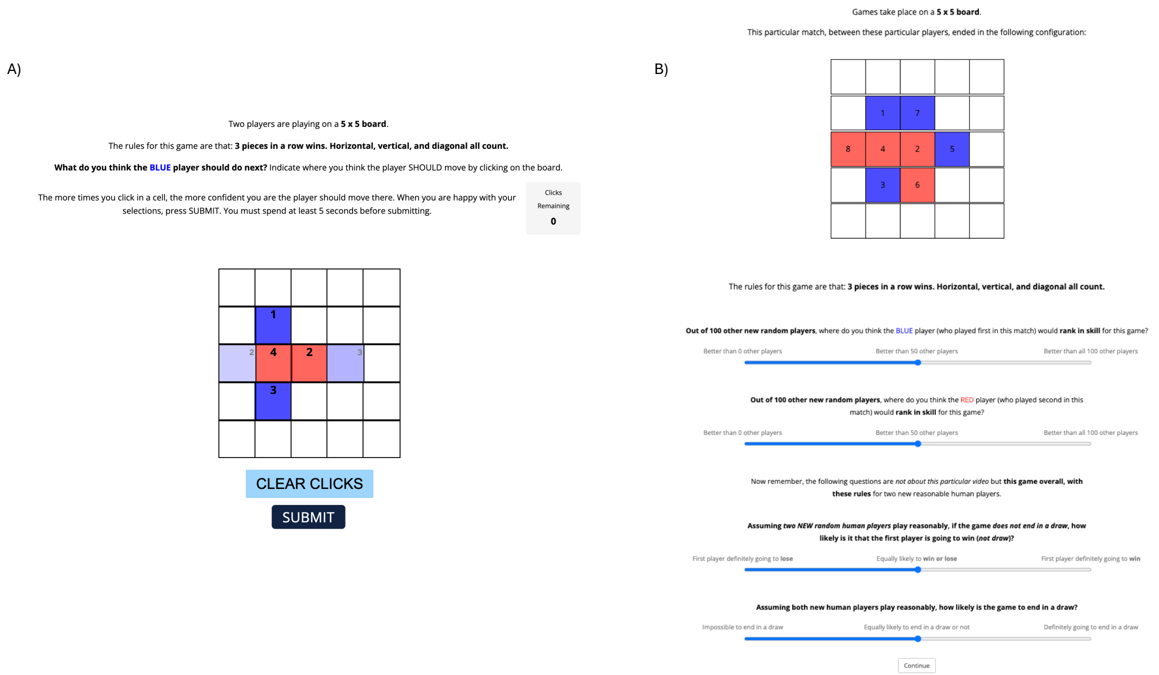

Figure 21: Example interfaces from the people watching others play and predicting moves study. Participants watch gameplay, which is frozen at three timepoints. At each timepoint, participants are tasked with predicting where a player should play by spreading 5 clicks over the board. **a,** The cells are colored in proportion to the number of clicks made by the participant, and the number of clicks remaining (out of 5) are shown to the participant. **b,** At the end of watching a full match, participants are shown a snapshot of the played match, and asked to make a series of judgments about the game via sliders.

of the 121 games (91) reach a termination condition (either Player 1 or Player 2 achieves their objective, or the board fills up and ends in a draw) within 30 moves. Simulated match lengths are shown in Figure 24.

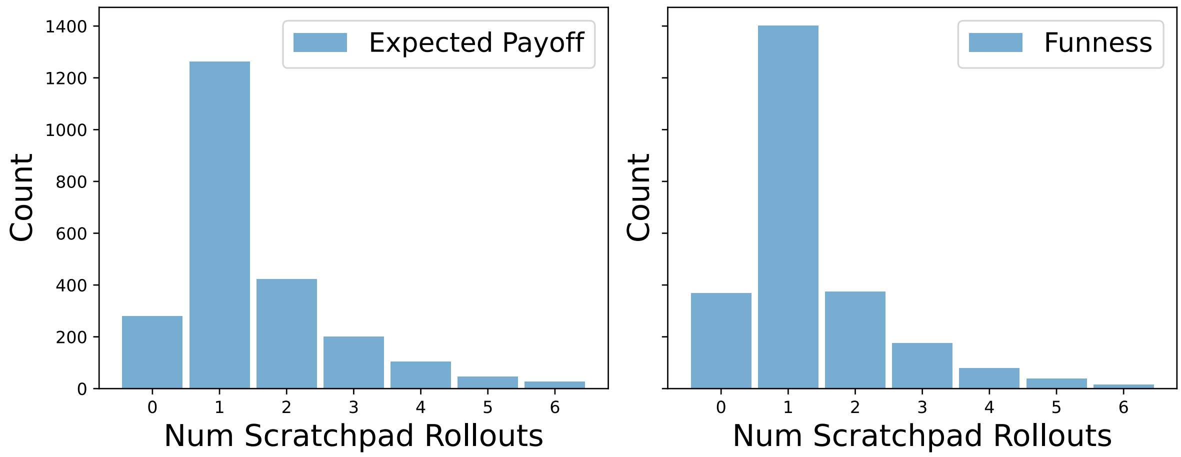

Figure 22: Scratchpad usage. Explicit scratchpad "rollouts" per game, per participant. A "rollout" is counted as any interaction with the scratchpad, involving at least one click. 2 rollouts means the participant pressed "RESET" once. Most participants engage at least once with any game, both when evaluating **a,** fairness and **b** funness; few conduct more than three explicit rollouts on the scratchpad.

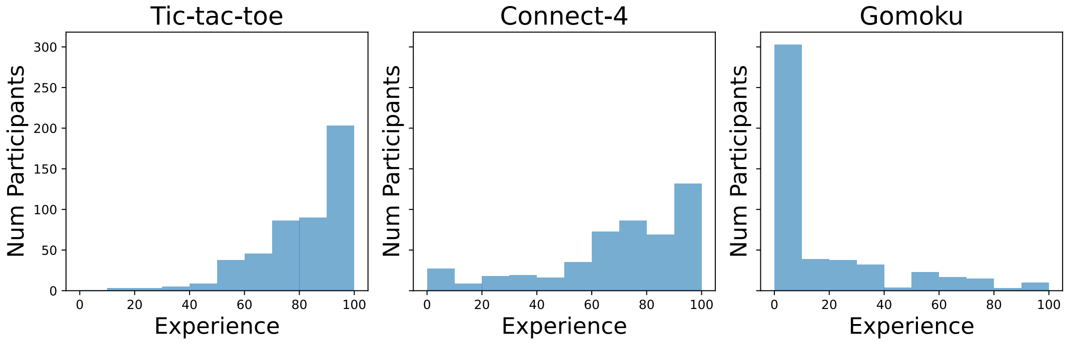

Figure 23: Self-reported prior game experience. Participants in the "just think" experiment indicated their prior experience with several existing games, namely, Tic-Tac-Toe, Connect-4, and Gomoku.

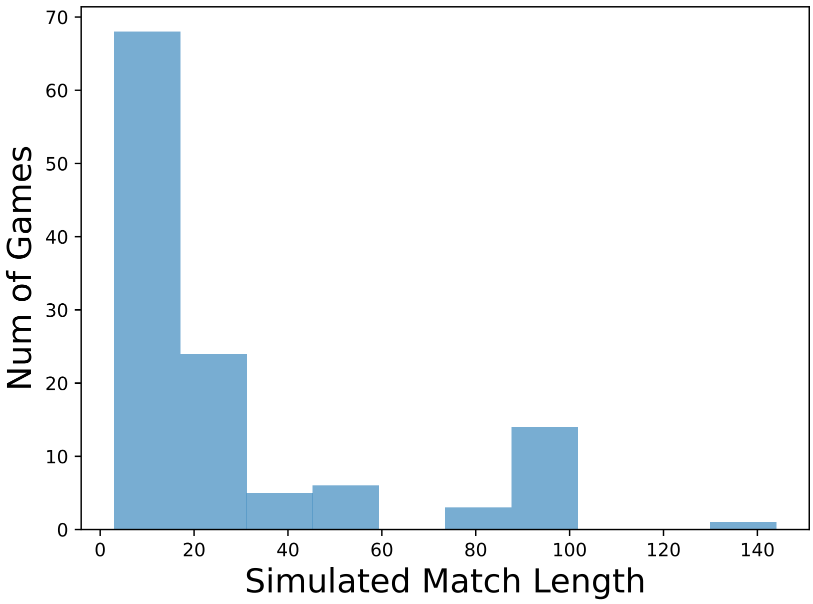

Figure 24: Intuitive Gamer simulated match length. Average simulated match length from the Intuitive Gamer reasoning module, for each of the 121 games. Simulations were run under the Intuitive Gamer player module.

5.4 Decomposed game outcome prediction tasks

Participants indicated their inferences about likely game outcomes about (1) if the game did not end in a draw, whether the first player was likely to win ($P(\text{P1 win} \mid \text{not draw})$); and (2) how likely the game was to end in a draw ($P(\text{draw})$). We compare the Intuitive Gamer model predictions against human judgments for these decomposed questions in Figure 25 and 26, respectively. The questions are critical together; e.g., if the participant thinks that the game will definitely end in a draw, the first question is ill-posed. This is accounted for in the payoff, and $P(\text{P1 win})$ when we combine $P(\text{P1 win} \mid \text{not draw}) \times P(\text{draw})$. We report participants' computed $P(\text{P1 win})$ and their reported $P(\text{draw})$ in Figures 25 and 26. We extract readouts of the corresponding questions to the the Intuitive Gamer model by counting the number of outcomes (in each bootstrapped set of $k = 6$ samples) that correspond to each query (e.g., the proportion of draws; or proportion of first player wins) and compare against alternate models using the same empirical outcome frequency in Figures 25 and 26, respectively. For cases where a game did not have any non-draw simulations, we take $P(\text{P1 win} \mid \text{not draw}) = 0.5$ (which is generally what we notice participants do; see Figure 27). In general, the Intuitive Gamer approaches the human split-half R^2 for the measures involving $P(\text{win})$ (i.e., split-half R^2 for $P(\text{P1 win}) = 0.82$ $[95\%\text{CI} : 0.77, 0.87]$; $P(\text{P1 win} \mid \text{not draw}) = 0.78$ $[95\%\text{CI} : 0.72, 0.83]$). However, the Intuitive Gamer (and alternate models) are all substantially sharper in $P(\text{draw})$ predictions than people and not near the noise ceiling (split-half human R^2 for $P(\text{draw}) = 0.78$ $[95\%\text{CI} : 0.72, 0.83]$). It is possible that people compute over a small sample of simulated games in a different way than counting outcomes, which may better correspond to the readouts they provided, or generally have a prior to believe that games are more likely to end in a draw. Our early explorations into the impact over the game reasoning module running only partial simulations, where simulations that stop early are taken to be a draw, may encode such a prior and improves fit to human draw judgments (Figure 28). However, we leave a close analysis of partial versus full simulations for future work, as it requires a deeper cross-comparison with alternate models and other stopping rules (which added substantial complexity to this first exploration of an Intuitive Gamer model).

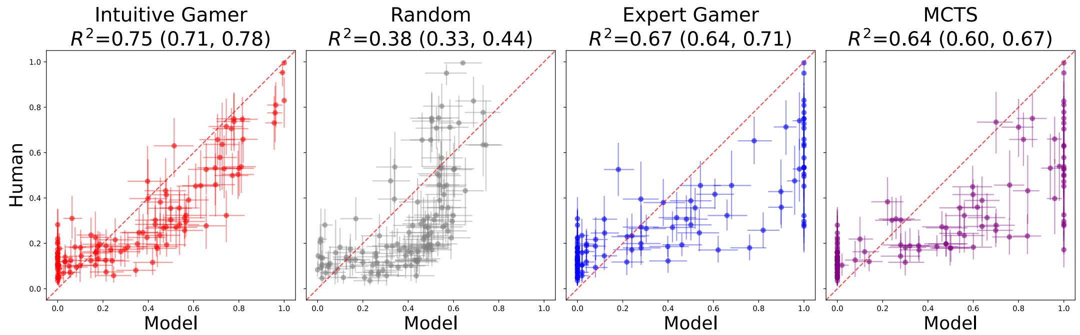

Figure 25: Empirical model comparisons for $P(\textbf{P1 win})$ against people. Bootstrapped 95% CIs over participants and samplings of $k = 6$ simulations for $N = 20$ simulated participants for each model.

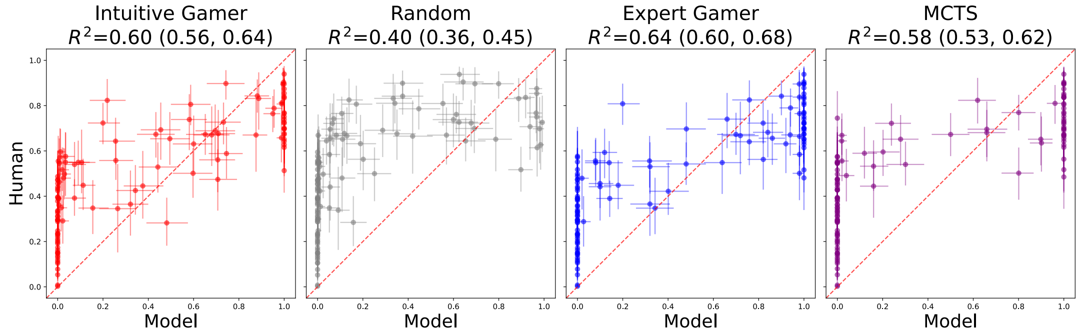

Figure 26: Empirical model comparisons for $P(\textbf{draw})$ against people. Bootstrapped 95% CIs over participants and samplings of $k = 6$ simulations for $N = 20$ simulated participants for each model.

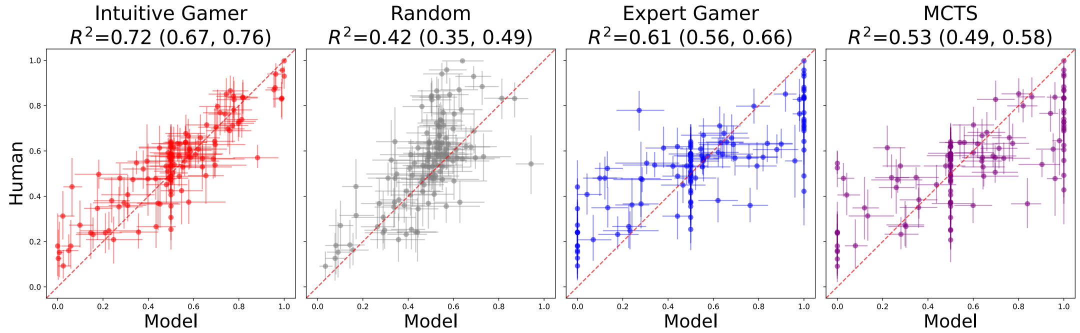

Figure 27: Empirical model comparisons for $P(\textbf{P1 win}|\textbf{no draw})$ against people. Games under the model that only have draws are imputed with 0.5. Bootstrapped 95% CIs over participants and samplings of $k = 6$ simulations for $N = 20$ simulated participants for each model.

5.5 Predicting payoff from non-simulation based linguistic features

A key component of our hypothesis is that people assess new problems by drawing on fast probabilistic mental simulations. This hypothesis demands a comparison then against non-

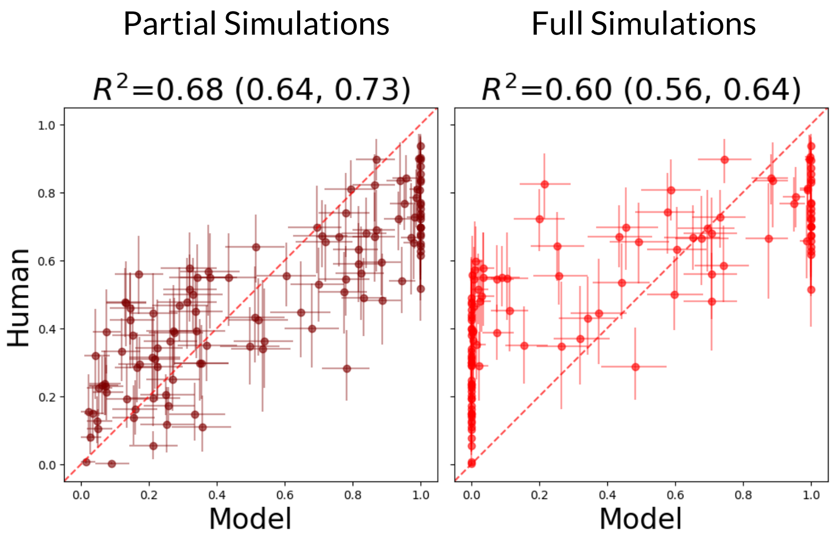

Figure 28: Full versus partial simulations to estimate P(draw). Comparing human and Intuitive Gamer model predicted P(draw) under partial versus full game simulations (both are computed with $k = 6$ simulations). Games that end early are deemed draws. Error bars depict 95% bootstrapped CIs over the average value for each game.

simulation-based alternate models. To assess the role of non-simulation-based features, we fit a linear regression model to the binary game traits (as introduced in the Methods). We fit to 70% of the games and test on the held-out 30%. We find that the model can capture some variance in human judgments $R^2 = 0.33$ [95% CI: $0.29, 0.37$] (see Figure 29) but comparatively less than models based on explicit simulation, as noted in the main text.

5.6 Language model payoff and game-theoretic optimal comparisons

We also compare against a series of language models—varying in prompting type (directly asking about game outcomes, or permitting "chain of thought" reasoning)—as well as a state-of-the-art reasoning model, o1 (76). We compare predictions under the language models against human predicted payoffs Figure 30 and against the "optimal" game theoretic expected payoffs in Main Text Table 2b. While o1 is closer to human judgments and a closer fit to the game-theoretic

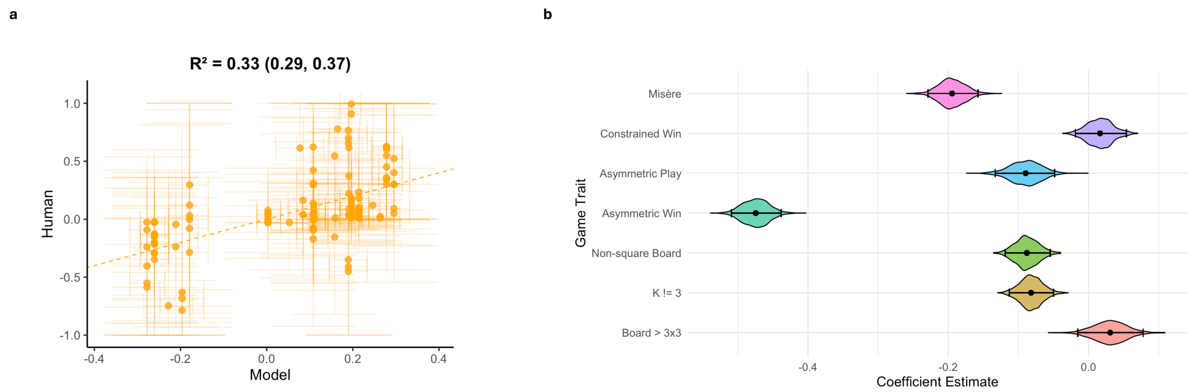

Figure 29: Predicting payoff from non-simulation game traits. Fitting a linear regression model to binary game traits captures some of the variance of payoff judgments. Each point in **(a)** is a game. The error bars are the 95% CIs over the human bootstrapped mean payoff and model-predicted mean payoff fit over bootstrapped subsets of 70/30% of games. **(b)** 95% CIs around the parameter fits per game trait.

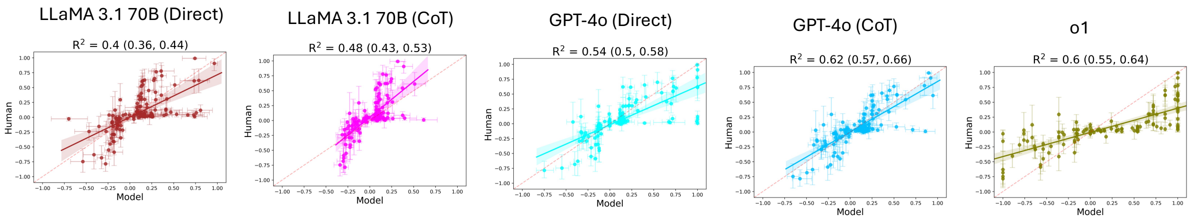

Figure 30: Comparing payoff predictions against language models. Correlations between bootstrapped samples of the human versus language (top) and "reasoning" (bottom) model predicted payoffs for the 121 games. 95% CIs are over 1000 bootstrapped subsamples.

optimal values, it is still a ways off of "perfect" highlighting that the games we consider here are non-trivial to reason about.

5.6.1 Language model prompting

All language and reasoning models are prompted with a variant of the instructions provided in the human experiment. We prompt models to provide (in a single response) with our two game outcome reasoning questions—how likely the first player will win if the game does not end in a draw, and how likely the game will end in a draw—directly following the questions asked of participants. We provide the models with the same $0 - 100$ slider labels as in the human study and ask the model to provide a value in the same range per question. We sample 20 rollouts for each model. We sample all models at the default temperature of 0.7, except o1, which we sample using its default fixed 1.0 temperature.

5.7 Reasoning about game funness

We next provide additional analyses into the funness model and features that matter for capturing the variance in the human data.

5.7.1 Component ablations

To assess the impact of the relative contribution of each funness model component, we lesion each component. Analysis of Variance (ANOVA) and Akaike information criterion (AIC) assessments highlight that each component contributes to the fit to human data (see Extended Data in the main text). We also assess the relative benefit for fitting each component with a non-linear spline in Table 5. We see a relative benefit only for the game length component. The balance feature collapses back to a single degree of freedom (linear fit).

Feature	F	p	ΔAIC
Balance	1.242	2.67e-01	-0.7
Reward for Thinking	1.082	3.00e-01	-0.9
Game Length	14.027	2.85e-04	11.8

Table 5: Impact of quadratic features in funness model. Comparing the inclusion of quadratic features on each of the simulation-based features in the funness model reveals that only a quadratic term for the game length feature significantly impacts fit to human judgments. ΔAIC is AIC_{lin} - $\text{AIC}_{\text{nonlin}}$ (higher indicates better fit from non-linear).

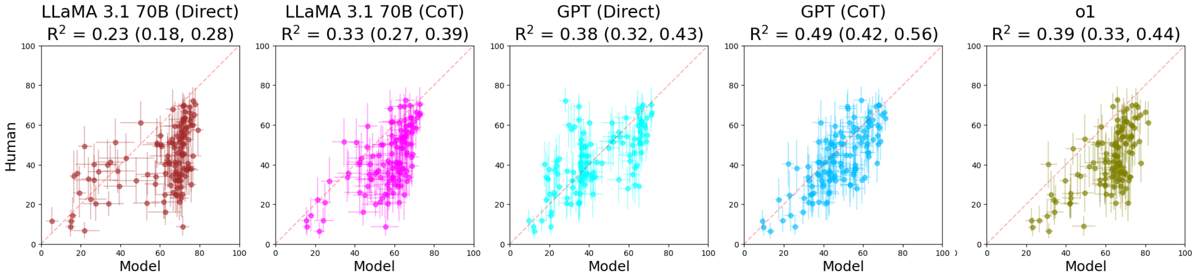

Figure 31: Language- and reasoning-model predicted funness per game, compared to people. Each point is one game. Error bars are bootstrapped 95% CI over the average human- and model-predicted funness.

5.7.2 Linguistic features

We additionally explore the role of linguistic non-simulation based features, as outlined in the Methods. Fitting a model to the binary traits alone captures a moderate amount of the variance in human funness judgments (Figure 32a) but comparatively less than the simulation-based models. Some of this may arise from relative correlations between the traits and the simulation-based features (Figure 32b), e.g., boards that have asymmetric win conditions are generally associated with unfair games (which could connect to the balance component). Adding in the binary traits to the simulation-based funness model does not parsimoniously improve fit to human data. We find minimal evidence for an effect from "novelty," computed as the number of binary traits (or deviations from Tic-Tac-Toe) that are "active." Adding in a linear component does induce a slightly better fit according to ANOVA and AIC tests (Table 6), but has a negligible on our generalization tests (Table 7)—that is, held-out fits when fitting to bootstrapped subsamples of 50% of the games. It is possible that some participants are primarily accounting for linguistic-only (non-simulation based) features when assessing funness; recall, we do see comparatively higher variability in participants' judgments for funness evaluations than payoff predictions. However, at the aggregate population level, accounting for simulation-based features dominates fits. We leave such participant-level modeling of funness for future work.

We also compare language- and reasoning-model predicted funness compared against human predicted funness in Figure 31. Models are prompted in the same way as in the payoff predictions described above; that is: using the default temperature of 0.7 and sampling 20 rollouts, under a slightly modified version of the full experiment instructions given to people, with the exception of o1 (sampled at its default 1.0). Most language models yield fits to human data that are comparably worse than the simulation-based features, with some exception to o1. We do not know what kind of simulation, if any, o1 is doing over these games; hence, the model at present does not support the kind of explanatory analyses we are interested in here. However, the comparisons point to potential value in using the human data collected here for benchmarking human-likeness of AI.

Added Feature	F	p	ΔAIC
Board Size	0.340	5.61e-01	-1.6
Approx Novelty	4.765	3.11e-02	2.9
Binary Traits	1.224	2.96e-01	-4.8

Table 6: Additions of linguistic non-simulation features to the funness model. Comparing the inclusion of linguistic non-simulation based features to the funness model reveals only a weak potential effect of incorporating non-simulation based features into the model. ΔAIC is $AIC_{sim\text{-}only}$ - $AIC_{expanded}$ (higher indicates better from including the additional feature).

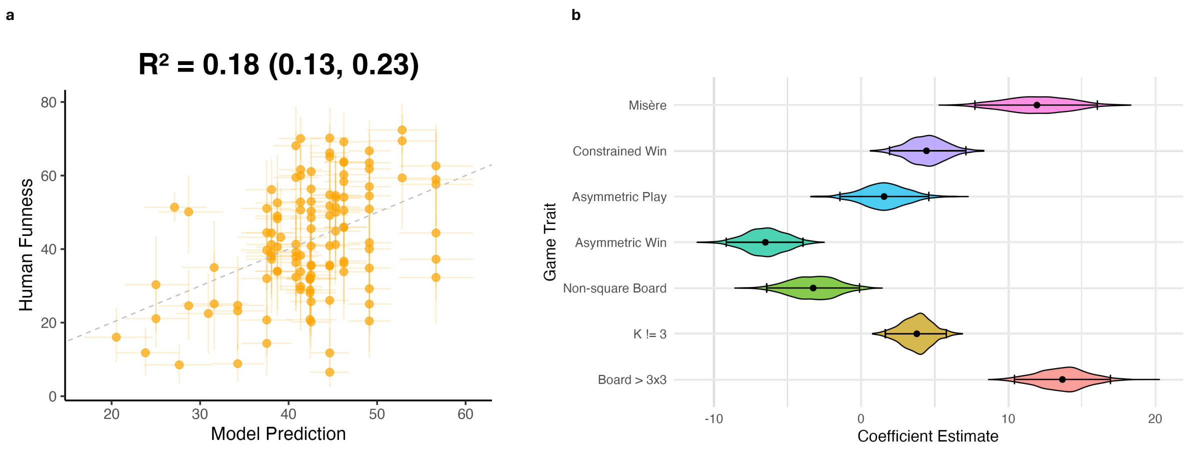

Figure 32: Predicting fun from non-simulation game traits. **a,** Modeling human funness judgments from non-simulation based binary game traits alone captures some of the variance in human judgments, however substantially less than the simulation-based model. **b,** Bootstrapped 95% CIs around the parameter fits from the funness model for each game trait.

Variant	Held-Out R^2
Simulation Only	0.62 (0.50, 0.71)
+ Linear Novelty	0.62 (0.53, 0.70)
+ Quadratic Novelty	0.61 (0.50, 0.69)

Table 7: Generalization test when incorporating non-simulation based features into funness model. Comparing held-out fits (fit on 50% of games, test on 50% of games) when including a "novelty" (linear or non-linear feature) ontop of the base regression model featuring only simulation-based features (balance; reward for thinking; quadratic length). This reveals that the additional linguistic feature does not meaningfully impact generalization.

6 Additional analyses into human and model action selection and prediction

We next present additional results from the "zero-shot human-human play" and "watch-and-predict" experiments. We report the full set of averaged log probabilities for each game in the human gameplay experiment in 33.

6.1 Action selection accuracy and rank

The Intuitive Gamer model generally is more accurate at capturing peoples' moves according to Top-1, Top-3, Top-5 accuracy of the moves people made (Figure 34). Accuracy is averaged over 100 bootstrapped samples to account for ties; for instance, to compute Top-1 accuracy, if the Intuitive Gamer assigns three moves to the same top probability, then accuracy is computed by sampling from that set. To account for ties, we also compute the average rank of the human played moves (including ties). However, random will appear best under this measure as all moves are assigned the same rank with uniform probability (therefore, appearing as if all played moves are assigned rank 1). To account for this, we also report the average number of moves assigned the same rank as the move played by people. Together, we see that generally the Intuitive Gamer assigns a relatively low rank to the played move (and not too many other moves to the similarly low rank).

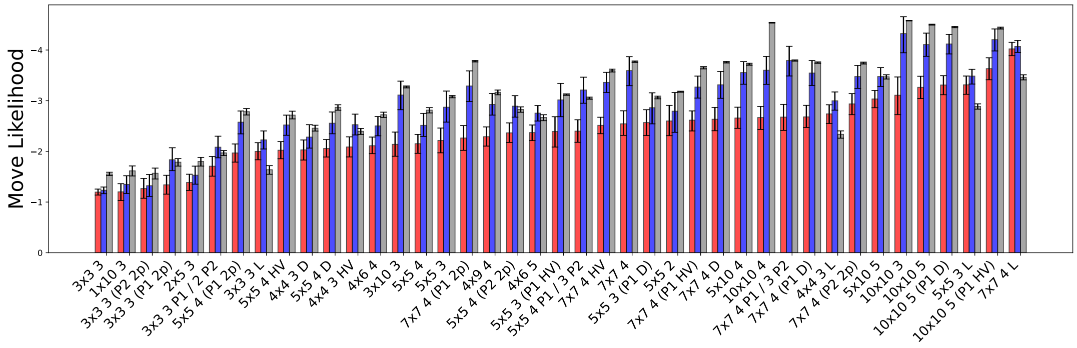

Figure 33: Aggregate move likelihood, per game, in the human-human play experiment. Aggregate move log probability (higher is better) for the moves people played in the human-human gameplay experiment under the Intuitive Gamer (red), Expert Gamer (blue), and random (grey) models, broken up by games. Error bars depict 95% bootstrapped confidence intervals around the mean log-likelihood over all moves for each game.

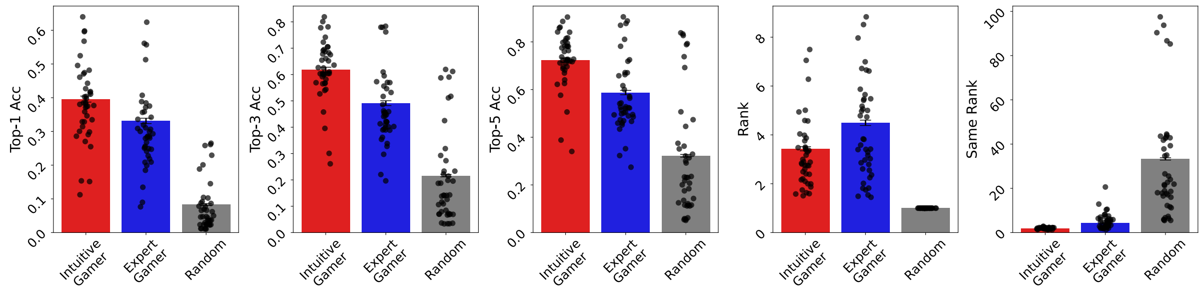

Figure 34: Accuracy and rank of human played move in human gameplay experiments. We additionally include additional statistics comparing the move distributions for the play experiments against observed play, particularly accuracy and the rank of the humans' played move relative to set of possible legal moves (ranks include ties). Each dot corresponds to the average for any one of the 41 played games.

6.2 Robustness to choice of distributional measure

To assess the robustness of our "watch-and-predict" experiment analyses to the measure of distributional similarity, we repeat our analyses using an alternative distributional measure: the Jensen-Shannon Divergence (77). The Intuitive Gamer again is generally at the human noise ceiling in average and similarly near the split-half noise ceiling for many (though not all, e.g., misere) games (see Figure 35). An admixture model over the model distributions to learn a mixing weights to fit the human watcher distributions (optimized using JSD) generally reveals that the Intuitive Gamer model is the dominant component across games—with exceptions for misere games and some other smaller-board games (see Figure 36), which warrants further investigation.

6.3 Modeling choices with a softmax-based model

As noted in the main text "Methods," when assessing how models capture the choices people actually made in games, we model participants' moves as some combination of the likelihood under the core model of interest (the Intuitive Gamer or Expert Gamer model) as well as some probability that they play randomly. We sweep over α between 0.5 to 0.95 in increments of 0.05. An alpha of 0.5 means that any move is equally weighted between the primary model (Intuitive Gamer or Expert Gamer) and random; higher alpha puts more weight on the primary model. The Intuitive Gamer model generally leads to better fits, independent of the choice of α. However, both models yield a better fit with human play and human watch distributions

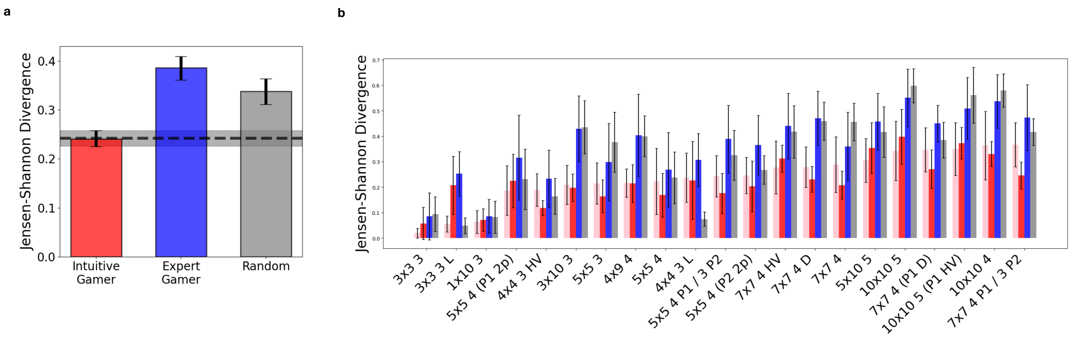

Figure 35: "Watch-and-predict" results are robust to the choice of distributional metric. Jensen-Shannon Divergence (lower is better) reveals that the Intuitive Gamer distribution are generally better aligned to the human watchers' distributions, over **a,** all game boards and **b,** at a per game level. Error bars in **a** depict bootstrapped 95%CI around the mean JSD for for all board; error bars in **b** depict standard deviation over boards per game.

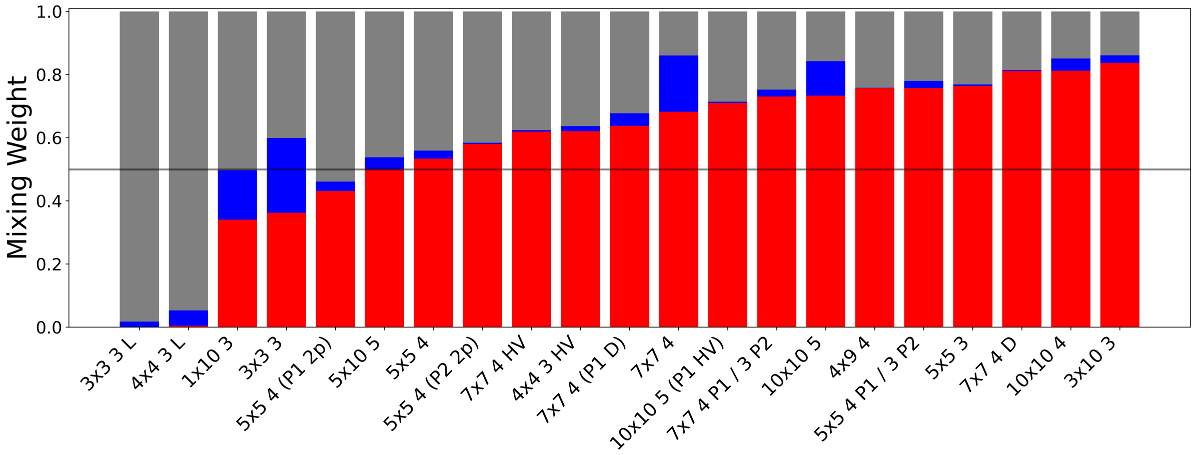

Figure 36: Average admxiture weights for each game, fit to the average human watch-and-predict distributions. The Intuitive Gamer is the dominant component for most games in an admixture fit over the models' distributions to the participants' distributions, optimized against the smoother Jensen-Shannon Divergence. Notable failure modes (e.g., misere games and comparable fits with the Expert Gamer model for Tic-Tac-Toe) align with places of weaker Intuitive Gamer model fits in the play data (see main text). Red means Intuitive Gamer, blue means Expert Gamer, grey means Random Gamer.

when mixed in some proportion with random. This aligns with the admixture results in the main paper, which show that fits for most games and most people are best modeled by some random component. A lower alpha (more weight to random) leads to a comparatively better fit when modeling human play and watch judgments under the Expert Gamer model, further indicating that the Expert Gamer model is likely more sophisticated than how people actually reason in new games for the first time.

In the admixture analyses, we also repeat admixture fits for different temperatures (τ), ranging in $\tau \in \{0.5, 1.0, 1.5, ..., 3.0\}$ where τ controls the action choice distribution softmax. We refit the admixture at each setting of τ and apply the same τ to both the Intuitive Gamer and Expert Gamer model. We do this to ensure that the relative fits in the admixture are as fair as possible to each model as the Expert Gamer model is often relatively sharp. As temperature increases, all models collapse toward random; however, as temperature decreases, we lose the probabilistic nature that is core to our Intuitive Gamer hypothesis. For most settings of τ, the Intuitive Gamer is the dominant mixing component; however, a higher τ appears to yield a slightly better fit for the watch distributional fits to human watch distributions relative to the

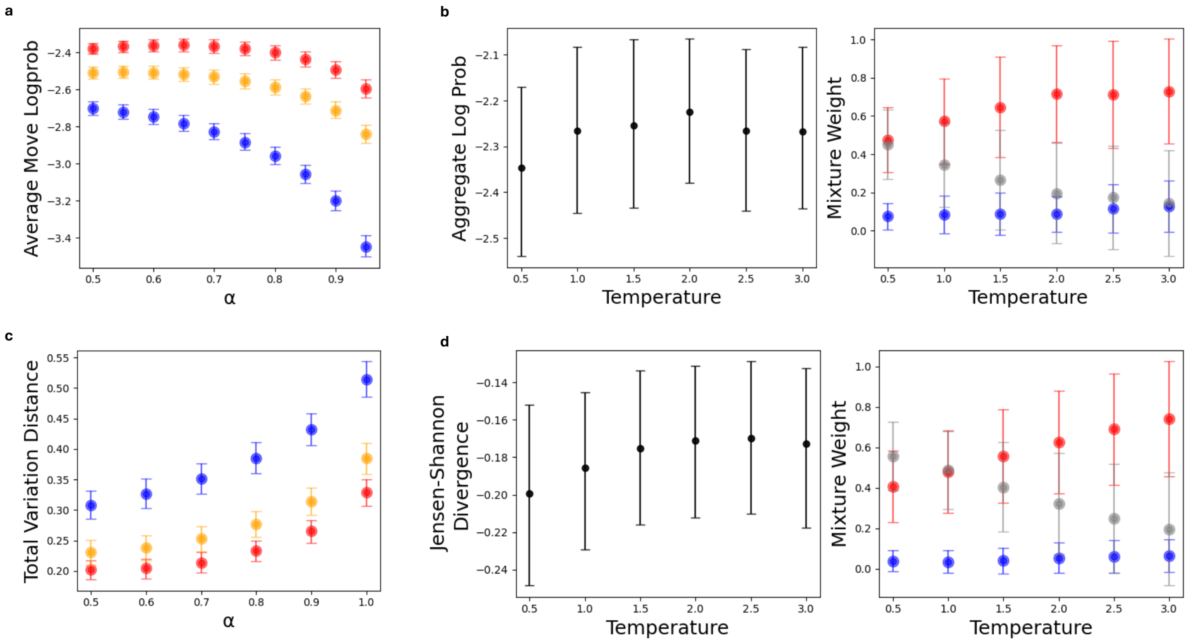

Figure 37: Mixture weight and temperature sensitivity in models' fit to people's action selection and prediction data. Varying the mixture weight α in the $\alpha-$softmax model for the core play **(a)** and watch **(c)** experiments. Error bars show 95% bootstrapped CI over the mean average move log probability (higher is better) and average Total Variation Distance (lower is better) relative to the human watcher move distribution. Red is the Intuitive Gamer model; yellow the depth-3 version of the Intuitive Gamer (non-flat); blue the Expert Gamer model. Admixtures are then fit over the flat Intuitive Gamer (red), Expert Gamer (blue), and random (grey) game agents for varied move distribution temperature for the play **(b)** and watch **(d)** experiments, respectively. Admixtures are fit for all data for each game. The left subplot depicts the average log probability of the moves in the resulting admixture for each temperature **(b)** and the resulting Jensen-Shannon Divergence (as the admixtures are optimized against JSD rather than TVD, given the smoothness of JSD) over the optimized mixture distribution. Error bars depict 95% CIs over the bootstrapped mean. The right subplot depicts the averaged mixing weights for each temperature value. Error bars depict standard deviation over the games.

played moves, which may be due to the seemingly higher variability in participants' move distributions.

6.4 Predicted probability of played move relative to human predictions

In the "watch-and-predict" experiment, participants were asked to predict where a player should move. To assess whether the distribution of moves people predicted captures how the human player actually played, the same aggregate analysis (as in the main "play" experiment), computing the average log likelihood of a player's action under the move distribution predicted by participants watching that match versus the distributions predicted under the Intuitive Gamer model and alternate models along a spectrum of expertise. Human watchers place similar probability on the move people actually made as the Intuitive Gamer model (Figure 38).

6.5 Additional human- and model-predicted distributions

We depict all aggregate human- and model-predicted distributions from the "watch-and-predict" study (see Figures 39- 45). Four matches, paused at three timepoints (during early-, middle-, and late-stage play) were shown for each game.

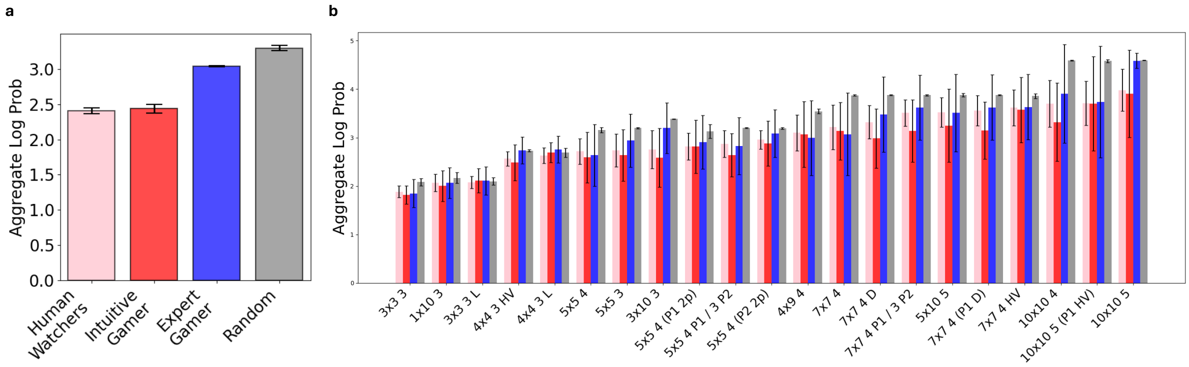

Figure 38: People's distribution of predicted moves capture real human player's actions comparably to the Intuitive Gamer model. a, From only a single indirect experience with a new game (watching one partial match), participants' distribution over predicted moves (pink) generally place similar probability on the move actually played to that predicted under the Intuitive Gamer model. The suggested moves made by the people predicting from watching videos (pink) to the actual move played by participants in each match and compare the log-likelihood under the human predictors' inferred move to the log-likelihood assigned by the respective models, marginalizing out α. Error bars depict bootstrapped 95% CIs over the averaged log-likelihood over boards, again sweeping over a "slop" parameter α (see "Methods"). **b,** Predicted move likelihood assigned to the move selected by the live player, broken down by game and ordered by lowest negative log likelihood (lower is better). Error bars depict standard deviation.

6.6 Game lengths

We compare the empirical observed game lengths in the play experiment against the expected game length under model simulations. Intuitive Gamer simulations show the highest correlations with empirical human game length data, while Expert Gamer is more correlated than the random agent (Figure 46). We observe that the Intuitive Gamer simulations rarely "overpredict", and there are several outlier games where it takes all models significantly longer to simulate than humans to finish.

6.7 Draw requests and surrenders

We next conduct exploratory analyses into draw requests and surrender decisions in individual matches against model and participant-predicted payoffs. Participants have high draw rates on matches that tend to be less fun and also less biased (see Figure 47a-b). This suggests that participants are sensitive to other features of games when making decisions about whether the request to end the game. Surrender rates are generally higher for less fun games, though show less of a trend based on game bias (see Figure 47c-d). Future work can work on richer process models of humans' draw and surrender decisions. The choice to request a draw instead of surrender is particularly worth investigating. If a player wanted to end the game, there should be no reason that they do not immediately surrender, as if they request a draw, the game only ends if the other player agrees to draw. Future modeling could explore other factors that people may consider when evaluating whether a game is worth playing (e.g., related to shame or dignity).

6.8 Evaluating games after a single exposure

After participants played a single match or watched and predicted actions in a single match, we asked them to either rate the expected payoff of the game overall or the funness. We also asked participants to rate the skill of their opponent (in the "play" condition) or the skill of both

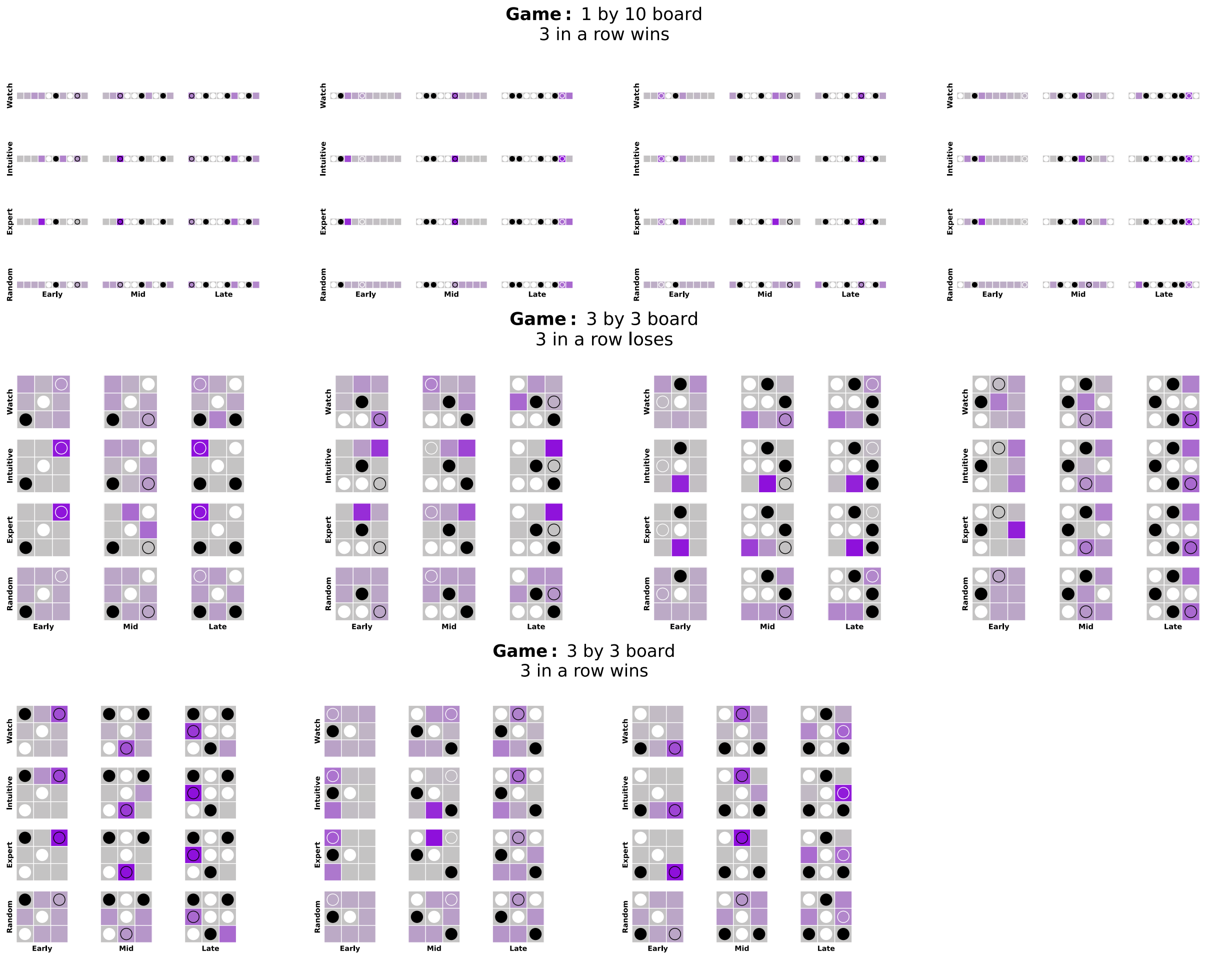

Figure 39: Full set of "watch-and-predict" distributions per match. As in the main text, the top entry in each panel depicts the aggregated human-predicted distribution over next moves, as determined by participants in the indirect play (watch-and-predict) study. The circle indicates where a person actually played. The color of each grid cell indicates where the player is predicted to move on that turn, under either the model- or human-predicted distribution; darker purple means a player is more likely to select that grid cell. We show all game boards and human- and model-predictions for each game type. The game board size and rules are written above each set of boards.

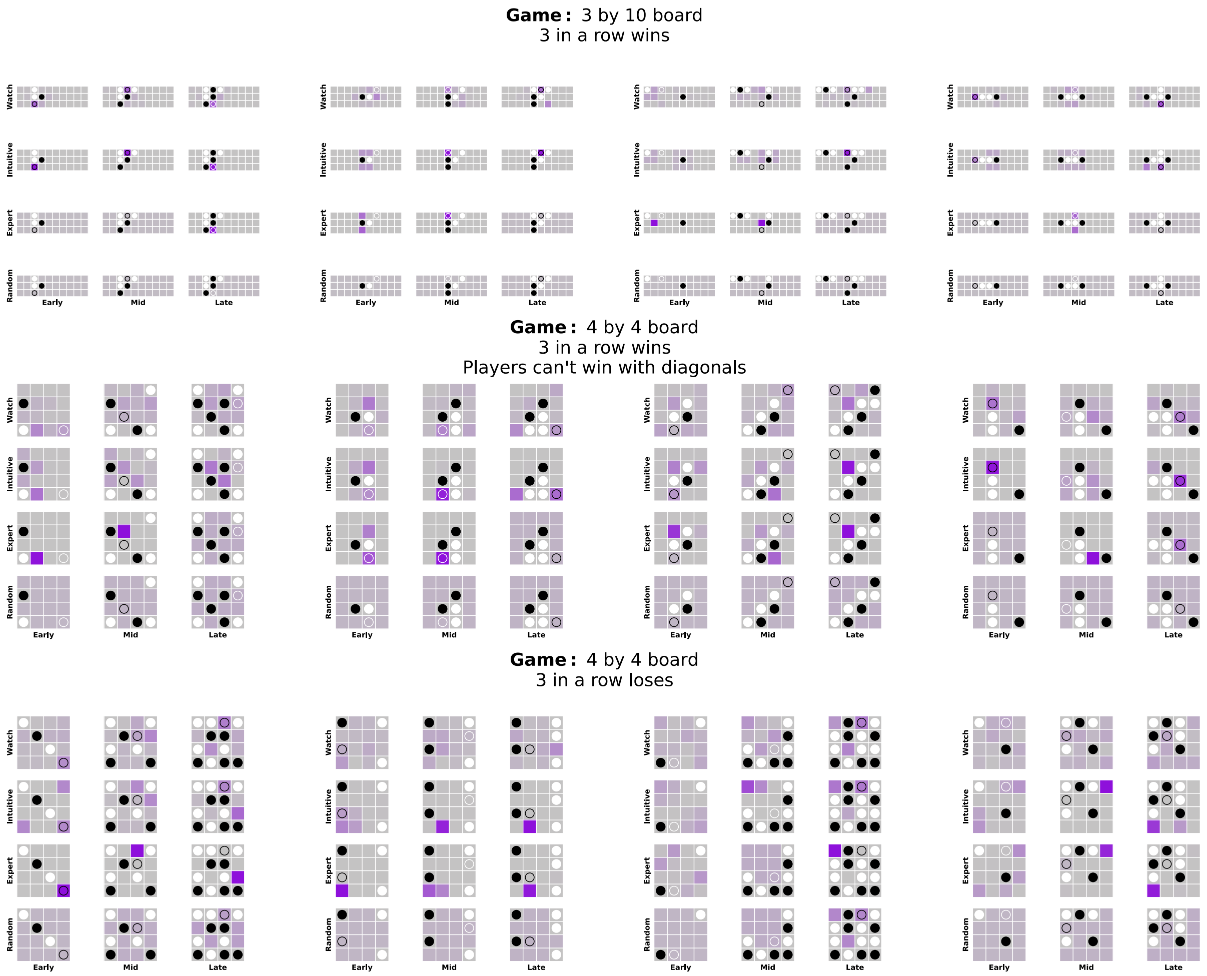

Figure 40: Full set of "watch-and-predict" distributions per match (continued).

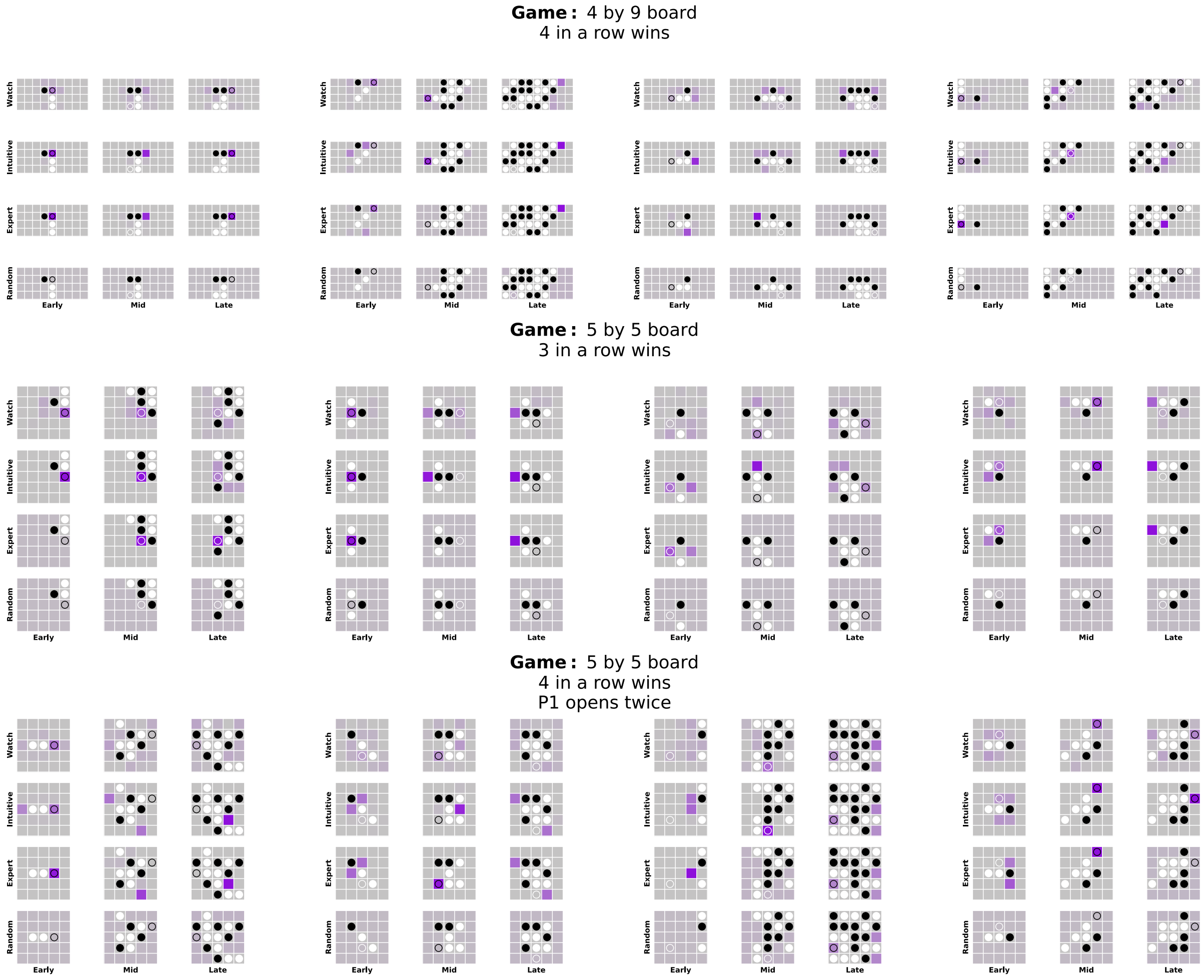

Figure 41: Full set of “watch-and-predict” distributions per match (continued).

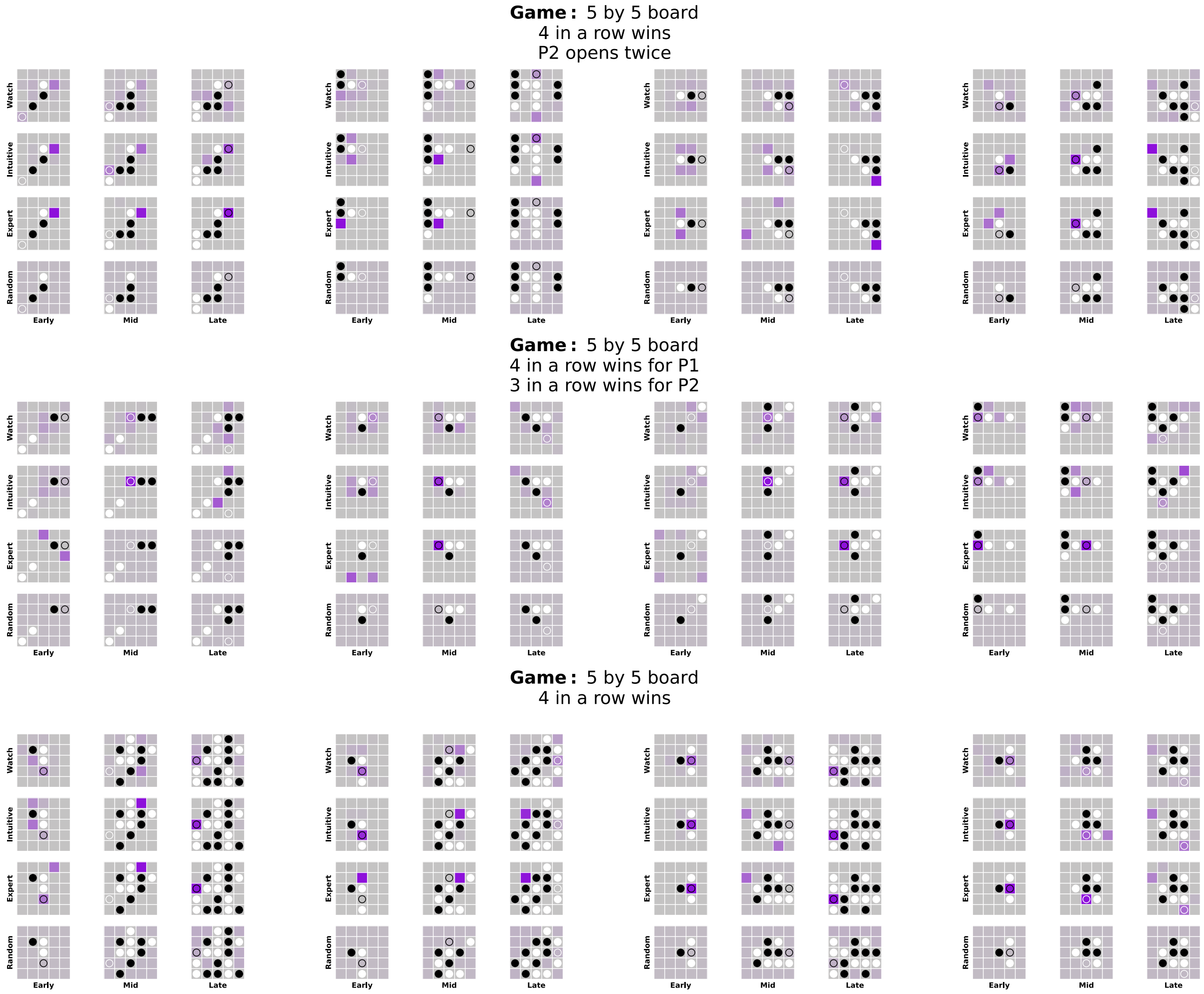

Figure 42: Full set of "watch-and-predict" distributions per match (continued).

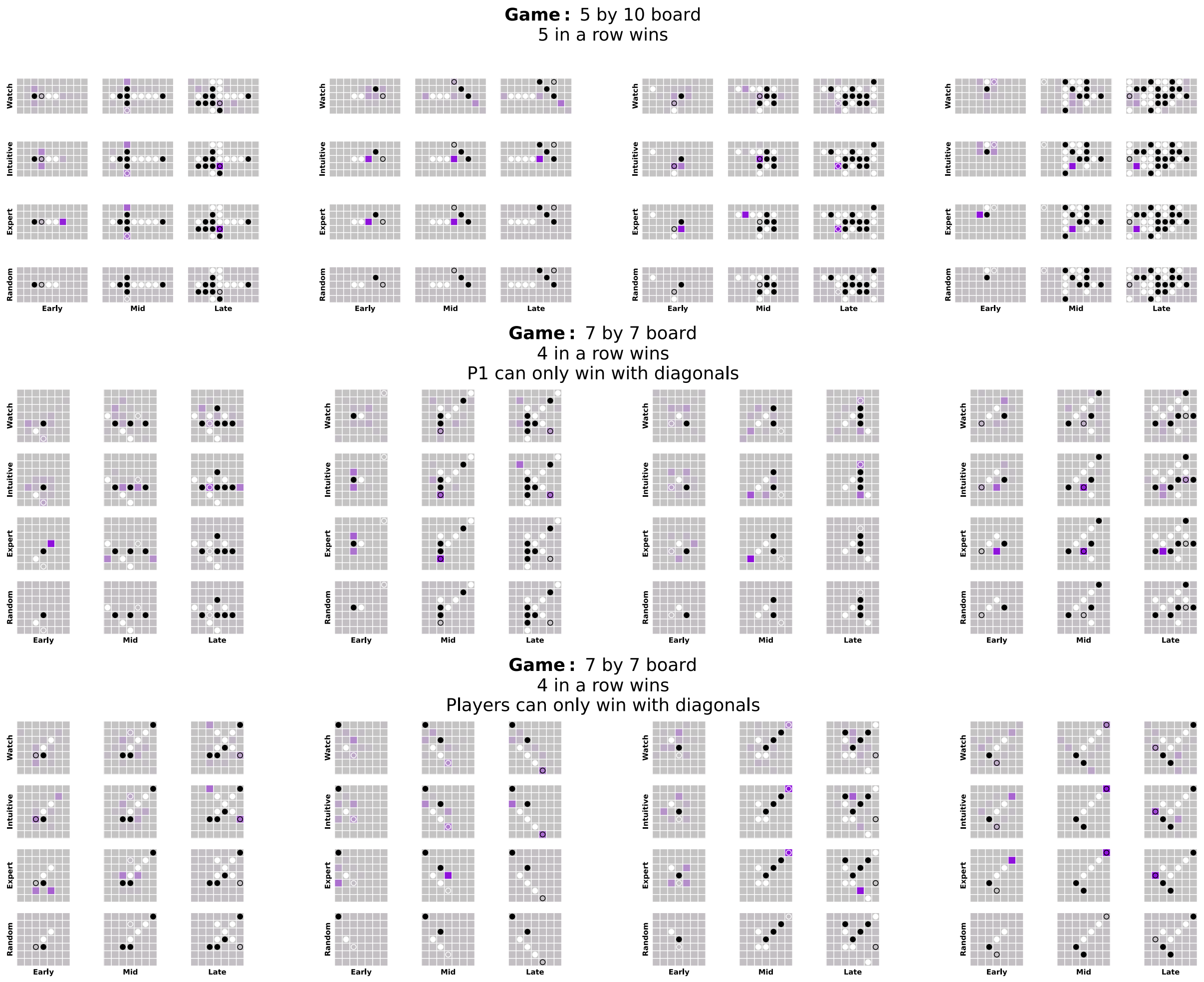

Figure 43: Full set of "watch-and-predict" distributions per match (continued).

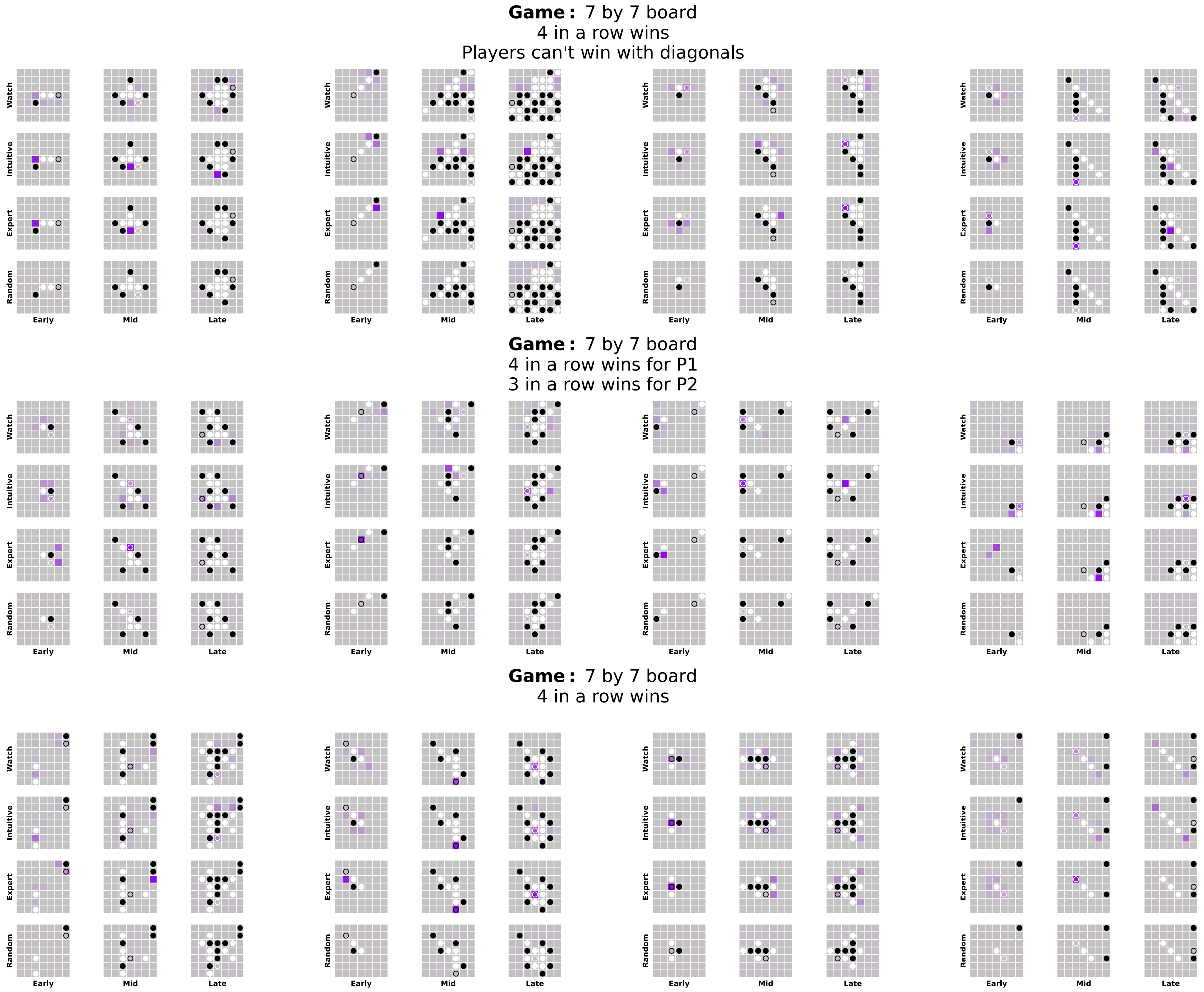

Figure 44: Full set of "watch-and-predict" distributions per match (continued).

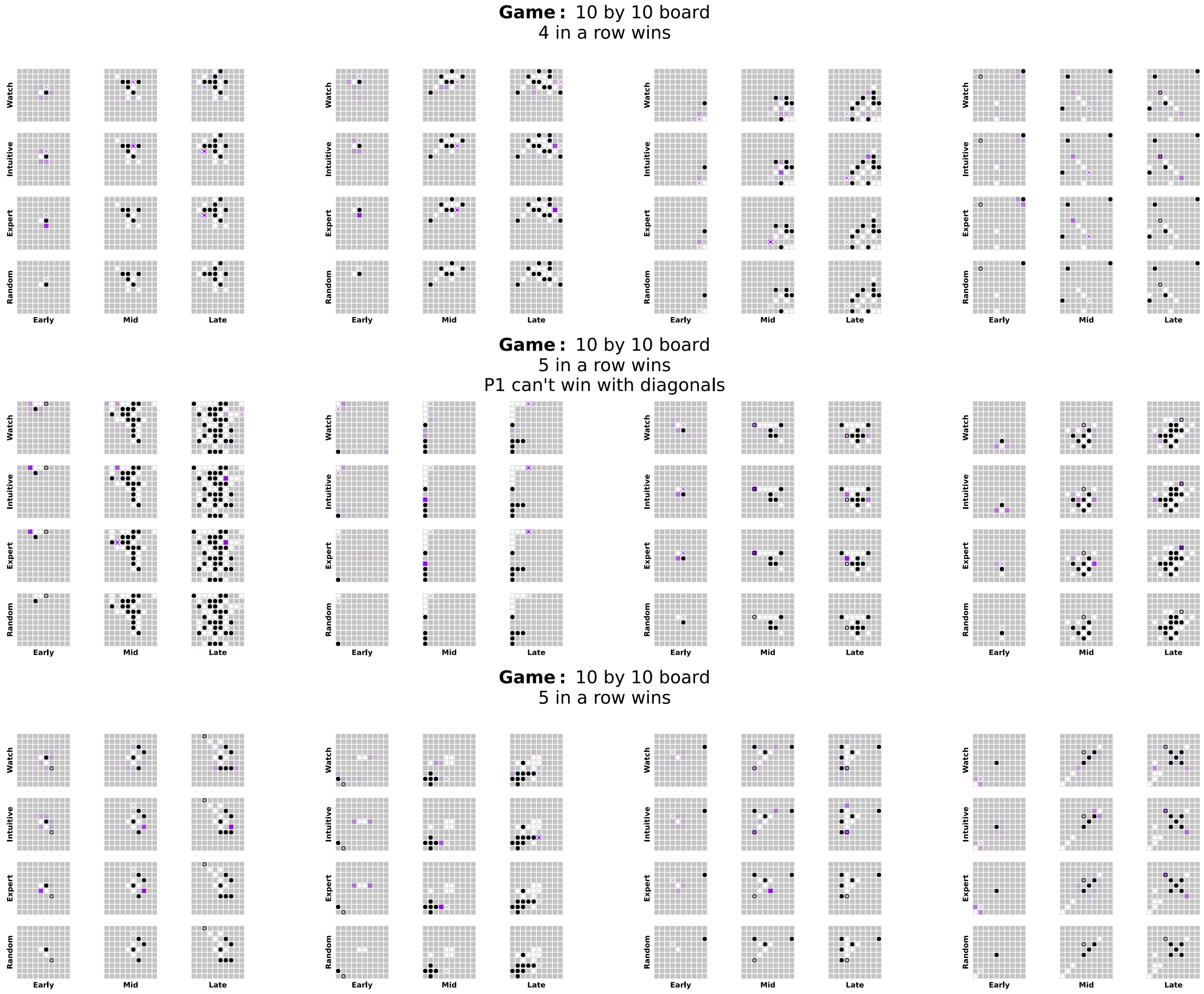

Figure 45: Full set of "watch-and-predict" distributions per match (continued).

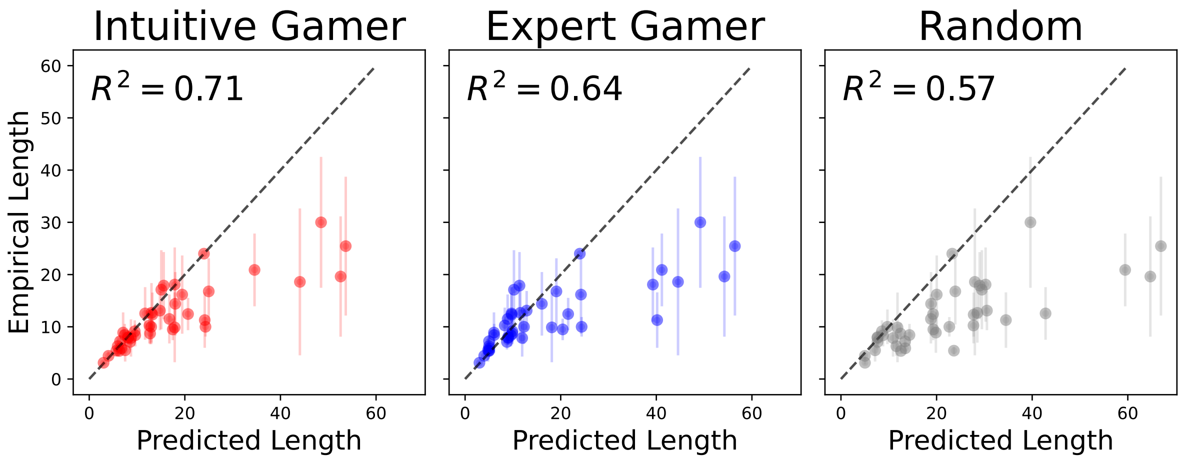

Figure 46: Empirical human- vs. model-predicted expected game length. Comparing the observed game length in human-human played games against the expected game length under simulations from the various game reasoning models. Game simulations are conducted between the same agent type (e.g., Intuitive Gamer against another instanced of itself). Empirical game length is coded as whenever the game ended: either from a player winning, the game ending in a draw. Games that ended early from an accepted draw request or a player surrendering are excluded.

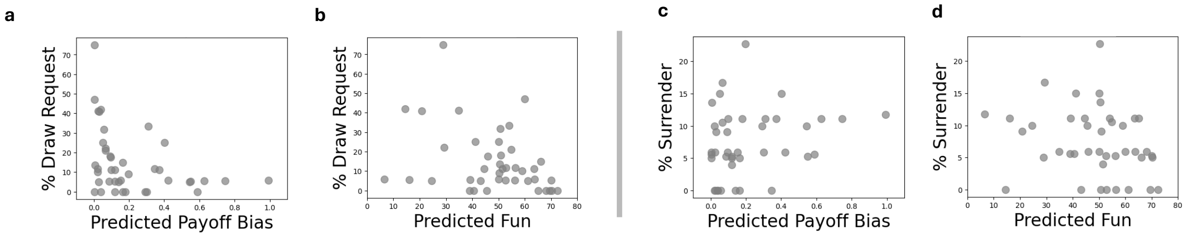

Figure 47: Draw and surrender rates and people's "just think" game evaluation. Frequency of draw requests (**a-b**) and surrenders (**c-d**) across all played matches per game (each point is a game). **a,** Games that are more biased (absolute difference in payoff from zero, under the averaged human-predicted payoffs in the "just think" study) are generally associated with higher draw rates. **b,** Games that are more fun (under the people's averaged funness judgments in the "just think experiment before any play) are generally associated with fewer draw requests. **c,** There is not a clear relationship between people's evaluation of game fairness (before any play) and surrender rates; **d,** in contrast, there games that are less fun tend to have somewhat higher surrender rates.

players (in the "watch" condition). We next conduct an initial exploration into these post-match game evaluations.

6.8.1 Evaluating games after one round of play

Participants' payoff predictions are highly correlated with their payoff predictions before any play and well-align with the Intuitive Gamer model (Figure 48). Participants' post-play funness judgments are less correlated with pre-play funness judgments (Figure 48) and are overall noisier (split-halfs $R^2 = 0.49$ [95% CI: $0.33, 0.64$] for post-play funness compared to a split-half of $R^2 = 0.69$ [95% CI: $0.56, 0.79$] for the same subset of 41 games rated on funness by participants who "just thought" about the games without any play). While we asked participants to assess the funness of the game overall—not just with respect to the match they played—it is possible people were biased in some form by the outcome of the game (Figure 50) or experience playing against the particular other player.

To assess the relative contribution of each simulated feature in our funness model, we rerun the same regression modeling analyses. The refit post-play funness model derived from the Intuitive Gamer features again reaches the split-half noise ceiling, capturing essentially all of the explainable variance in participants' judgments (Figure 49). Reward for thinking and game balance generally matter less for a fun game in people's post-play judgments compare to game length (Figure 49); however, we caveat that the post-play judgments are generally more variable (as noted above in the split-halfs).

Added Feature	F	p	ΔAIC
Board Size	7.651	9.00e-03	6.2
Approx Novelty	1.838	1.84e-01	0.1
Binary Traits	2.043	8.32e-02	2.5

Table 8: Assessing impact of non-simulation based features on funness model. Comparing the inclusion of non-simulation based linguistic features into the funness model fit to peoples' post-play funness judgments. ΔAIC is $\text{AIC}_{\text{sim-only}}$ - $\text{AIC}_{\text{expanded}}$ (higher indicates better from including the additional feature). There is a slight effect from incorporating either board size or all binary game traits (in contrast to adding an aggregate "count" of the number of traits that are "on" for any game, i.e., "approximate novelty"). It is possible the potential benefit of incorporating board size is related to the seemingly higher role game length plays in people's funness judgments post-play.

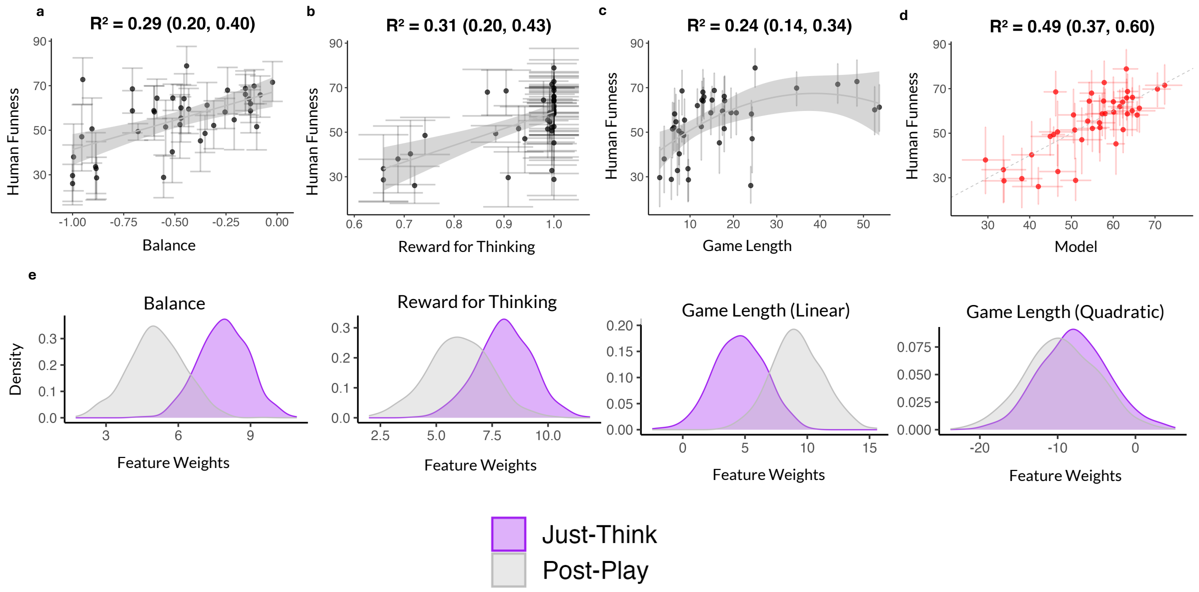

Figure 48: Impact of a single instance of play experience on judgments. **a,** bootstrapped mean predicted payoff for "just think" versus "post-play" participant judgments; **b,** bootstrapped mean predicted funness from "just thinking" versus "post-play".

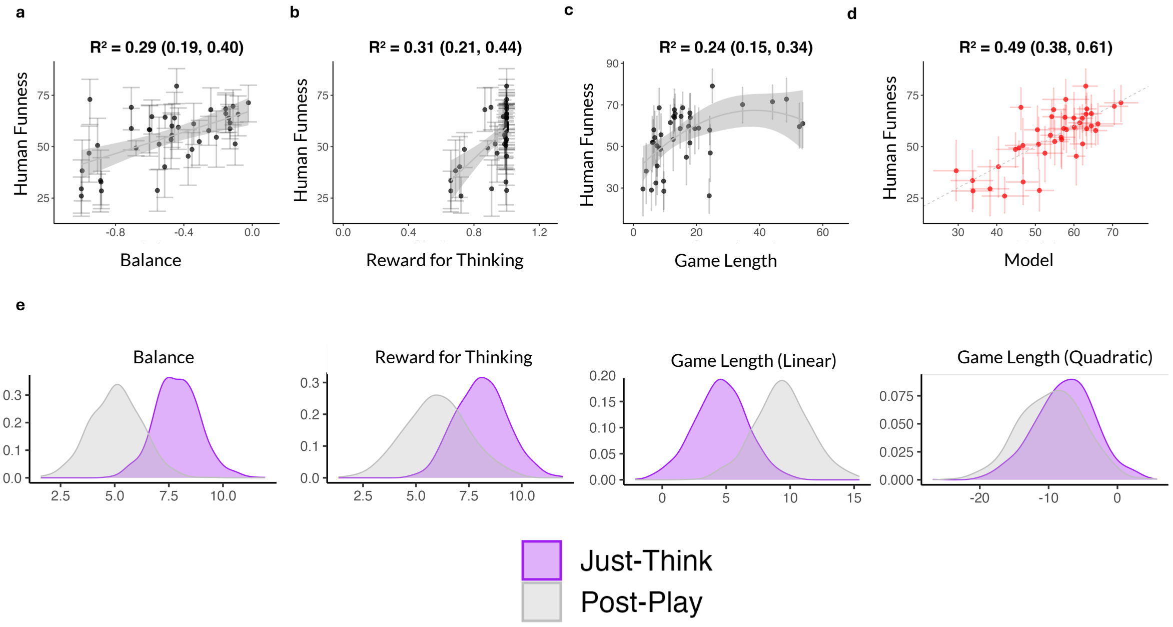

Figure 49: Post-play funness modeling. **a-c,** Features derived from the Intuitive Gamer model compared against post-play participant funness judgments. **d,** Regression model fit to the post-play data. 95% CIs are bootstrapped over participant ratings per games. **e,** Comparison of bootstrapped funness model parameter fits for the 41 games using the just think human ratings (purple) versus the ratings provided by participants after one round of play (grey).

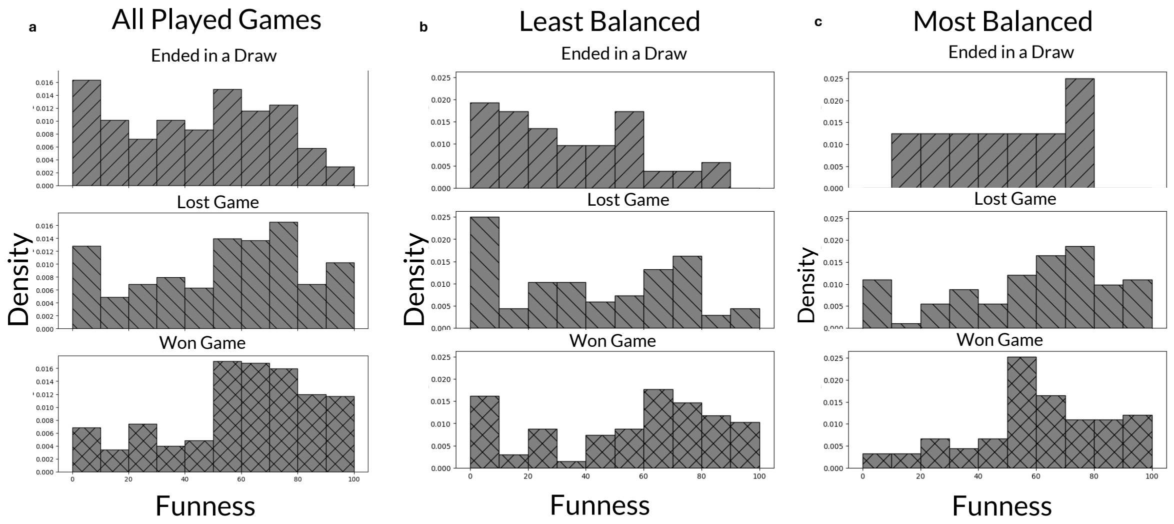

Figure 50: Relationship between attained outcome and funness judgments, after a single round of play in a new game. Funness judgments made by players after each match, broken down by whether they drew (top), lost (middle), or won (bottom). Even in cases where players lost, many still found the game fun. **a,** Funness for all matches for all played games. **b-c,** Post-play funness judgments for matches in the least balanced games (according to the Intuitive Gamer "balance" feature; the bottom 25 percentile; **b**) and the most balanced games (upper 75 percentile; **c**). Winning in an unbalanced game (**b**) still tends to lead to somewhat higher funness ratings; losing in a balanced game can also still lead to relatively high funness ratings.

6.8.2 Evaluating games after one round of watching

Participants' judgments after watching a single match are highly noisy for both payoff and funness queries (split-half $R^2 = 0.54$ [95% CI: $0.30, 0.76$] and $R^2 = 0.07$ [95% CI: $0.0001, 0.27$] for post-watch payoff and funness, respectively). In contrast to both the "just think" and post-play funness ratings which saw Tic-Tac-Toe rated around 50 (average fun rating of 51.4 ± 29.5 SD for "just think," and average fun rating of 49.4 ± 21.8 SD for post-play), the watchers rated Tic-Tac-Toe at an average of 77.6 ± 16.6 SD on funness. We observe that most funness scores are inflated and payoffs collapse towards zero (Figure 51). We posit that the increased variability in the post-watch participants' judgments may have arisen from participants not having external pressure to "force" some degree of thinking. Notably, participants in the watch study were not held from submitting their answers until one minute passed as in the "just think" experiment (nor given an optional scratchpad) before making their judgments, which may have impacted effort. Participants in the post-play experiment also were required to spend time before responding (at least 30 seconds); however, post-play participants were not given an optional scratchpad after they played their game. Future work should explore the role of forced response time on effort in encouraging thinking about game evaluations.

7 Exploratory analyses with a intermediate depth model

While our primary model focuses on highly computation-bounded, single-step look-ahead, our model class can naturally be extended to capture behavior of some game reasoners who may think more. We next consider a variant of the Intuitive Gamer model that incorporates the potential heuristic value of likely subsequent states in addition to the current state. This intermediate depth (Intuitive Gamer depth-3) model—so-called because it considers the current state, the opponent's likely response, and the possible responses to those actions—represents a

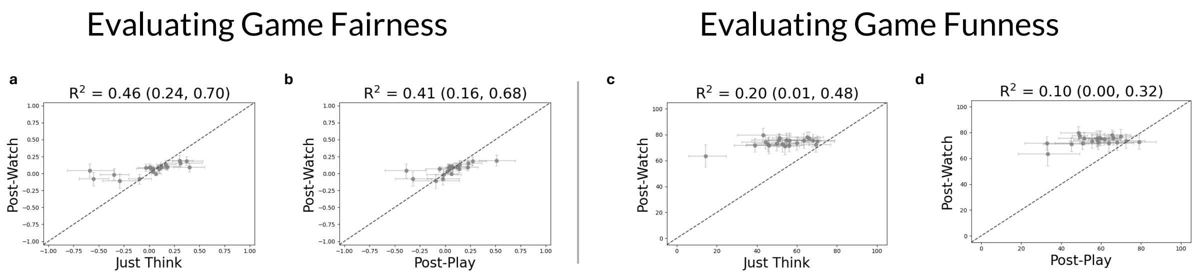

Figure 51: Judgments after watching a match. Comparing post-watch judgments of predicted payoff against those made in the **a,** "just think" and **b,** "play" experiments, on only the subset of 21 games considered in the watch experiment, as well as the predicted funness for **c,** "just think" and **d,** after play. Participants in the post-watch experiment are generally more variable with each other; the noisier responses may be due to not having been held for 30 to 60 seconds before submitting their judgment like the other studies.

moderate increase in computational load, though still requires far less computation than the Expert Gamer model.

7.1 Model definition

The primary difference of the Intuitive Gamer depth-3 model and our primary model is the value function. Concretely, the value assigned to a position under the Intuitive Gamer depth-3 model is as follows:

$$\tilde{\mathcal{V}}^3(s_t, a_t) = \tilde{\mathcal{V}}(s_t, a_t) + \beta \times \mathcal{V}^{\text{next}}(s_t, a_t). \tag{9}$$

To compute $\mathcal{V}^{\text{next}}(s_t, a_t)$ we first apply the action a_t to obtain the subsequent state s_{t+1} and pass the turn to the opponent. We then assume that the opponent takes the most likely action under the Intuitive Gamer model, or the action that maximizes $\tilde{\mathcal{V}}(s_{t+1}, a_{t+1})$. This results in the state s_{t+2} and returns play to the original player. We then say that

$$\tilde{\mathcal{V}}^{\text{next}}(s_t, a_t) = \max_{a \in \mathcal{A}} \tilde{\mathcal{V}}(s_{t+2}, a).$$

In special cases where either the original player or opponent move twice in a row, we either decrease or increase the depth of the simulation by one move. In each case, s_{t+2} is the state in which the original player takes another turn. Actions are selected via softmax sampling over $\mathcal{V}^3$, as in the standard Intuitive Gamer model. For our experiments, we set the discount factor β to 0.5.

7.2 Results

We report predicted payoffs under the Intuitive Gamer depth-3 model in Figure 52, as well as aggregate match log likelihoods and measure of distributional alignment (under TVD) to the play and "watch-and-predict" data, respectively (Figure 53 and Figure 54). As expected, this depth-3 model is a slightly worse fit to human behavior than the standard flat (shallow; depth-1) Intuitive Gamer, though it still outperforms the expert model in many cases. Notably, the differences between the depth-3 and depth-1 models largely disappear in board states toward the end of a game (see Figure 53, right). This might be due to the fact that there are fewer possible moves in such states (making a deeper search easier to complete) or because the final outcome is more salient (encouraging the expenditure of more computational resources on search). We leave further analysis of these possibilities to future work.

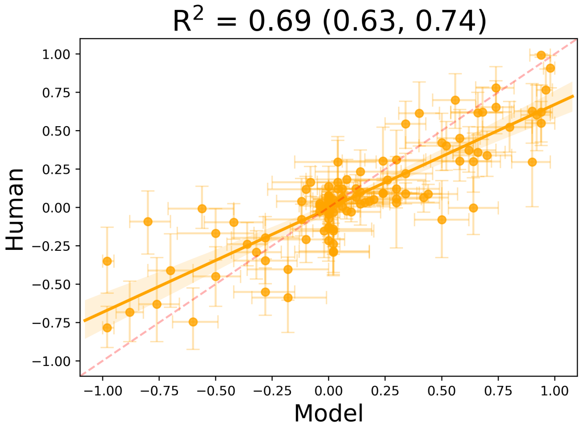

Figure 52: Intermediate depth payoff predictions. Non-flat (depth-3) variant of the Intuitive Gamer model's predicted payoffs for the 121 games in the "just think" experiment, compared against people's predicted payoffs. Error bars depict 95% CIs over bootstrapped human means and bootstrapped model-predicted payoffs under $k = 6$ simulated games for 20 simulated participants.

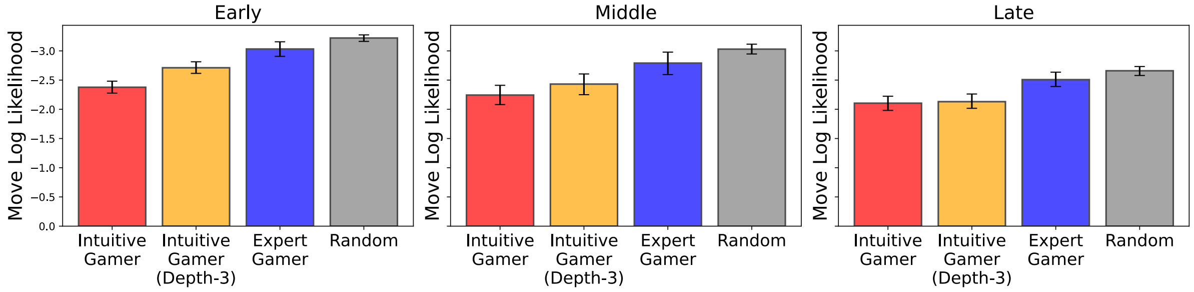

Figure 53: "Human-human play" analyses broken down by game stage. The bar plots show the log likelihood of an observed subjects' move under our different models. Error bars depict 95% bootstrapped confidence intervals around the mean log-likelihood over all games.

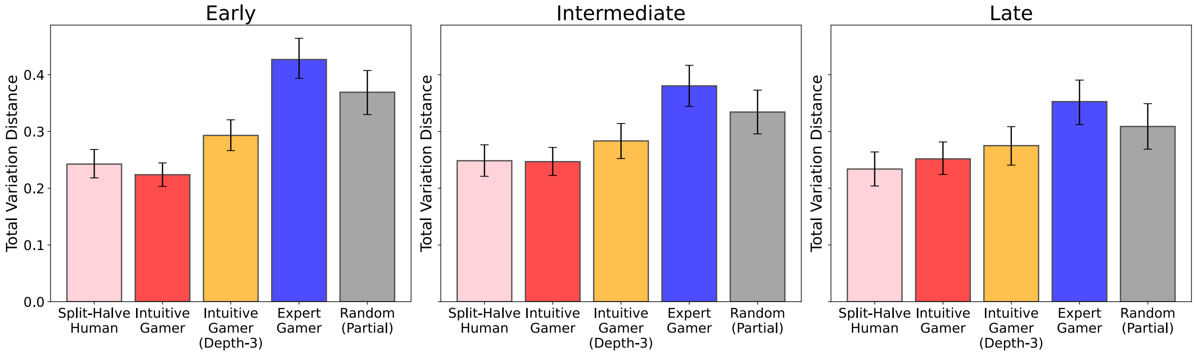

Figure 54: "Watch-and-predict" analyses broken down by game stage. The bar plots show the Total Variation Distance (lower is better) of model distributions to the human watchers' distribution, as well as compared to the split-half human watcher TVD.

8 Game list

We also include the full list of the 121 games we explored in this study below. For each game, we include the average human funness rating and average human payoff prediction. We also include the averaged decomposed scores (i.e., $P(\text{draw})$ and $P(\text{P1 win})$, where $P(\text{win})$ is computed from each participants' $P(\text{draw})$ and $P(\text{P1 wins}|\text{not draw})$ as described in Section 5.4.

Board	Rules	Fun	Payoff	P(Draw)	P(P1 Wins)
5x5	3 pieces in a row loses.	72.4	0.2	0.4	0.4
10x10	4 pieces in a row wins.	70.2	0.1	0.5	0.3
5x10	4 pieces in a row wins.	70.1	0.1	0.6	0.3
4x4	3 pieces in a row loses.	69.4	0.0	0.5	0.2
10x10	5 pieces in a row wins. The first player can place 2 pieces as their first move, while the second player can only place 1 piece as their first move.	69.2	0.3	0.4	0.5
5x5	3 pieces in a row wins.	68.1	0.3	0.3	0.5
10x10	4 pieces in a row wins. However, a player can only win by making a diagonal row. Horizontal and vertical rows do not count.	66.7	0.2	0.6	0.3
10x10	5 pieces in a row wins.	66.2	0.2	0.5	0.4
7x7	4 pieces in a row wins.	65.1	0.2	0.5	0.4
7x7	4 pieces in a row wins. The first player can place 2 pieces as their first move, while the second player can only place 1 piece as their first move.	63.8	0.4	0.4	0.5
10x10	5 pieces in a row wins. The second player can place 2 pieces as their first move, while the first player can only place 1 piece as their first move.	63.6	-0.1	0.5	0.2
7x7	4 pieces in a row wins. However, a player can only win by making a diagonal row. Horizontal and vertical rows do not count.	63.5	0.1	0.6	0.2
5x5	4 pieces in a row loses.	62.6	0.1	0.7	0.2
10x10	5 pieces in a row wins. However, a player can only win by making a diagonal row. Horizontal and vertical rows do not count.	61.8	0.1	0.6	0.2
4x12	4 pieces in a row wins.	61.6	0.1	0.6	0.3
InfxInf	5 pieces in a row wins.	61.3	0.1	0.3	0.4
7x7	Each player needs 4 pieces in a row to win. The first player cannot win by making a diagonal row (only horizontal and vertical rows count), but the second player does not have this restriction.	61.1	-0.1	0.5	0.2
10x10	4 pieces in a row wins. The first player can place 2 pieces as their first move, while the second player can only place 1 piece as their first move.	60.2	0.6	0.2	0.7
5x10	5 pieces in a row wins.	60.0	0.0	0.8	0.1
4x4	3 pieces in a row wins.	59.5	0.5	0.3	0.6
10x10	3 pieces in a row loses.	59.4	0.0	0.4	0.3
7x7	4 pieces in a row loses.	58.9	-0.0	0.6	0.2
10x10	4 pieces in a row wins. The second player can place 2 pieces as their first move, while the first player can only place 1 piece as their first move.	58.4	-0.2	0.5	0.2
10x10	5 pieces in a row loses.	57.6	0.1	0.5	0.3
10x10	4 pieces in a row wins. However, a player cannot win by making a diagonal row. Only horizontal and vertical rows count.	57.0	0.2	0.5	0.4

Board	Rules	Fun	Payoff	P(Draw)	P(P1 Wins)
10x10	Each player needs 5 pieces in a row to win. The first player cannot win by making a diagonal row (only horizontal and vertical rows count), but the second player does not have this restriction.	56.4	-0.3	0.4	0.1
5x5	The first player needs 4 pieces in a row to win, but the second player only needs 3 pieces in a row to win.	56.2	-0.6	0.2	0.1
5x5	4 pieces in a row wins.	54.8	0.1	0.7	0.2
5x5	3 pieces in a row wins. However, a player cannot win by making a diagonal row. Only horizontal and vertical rows count.	54.6	0.3	0.4	0.5
10x10	5 pieces in a row wins. However, a player cannot win by making a diagonal row. Only horizontal and vertical rows count.	54.5	0.1	0.7	0.2
5x5	4 pieces in a row wins. The first player can place 2 pieces as their first move, while the second player can only place 1 piece as their first move.	54.1	0.3	0.4	0.4
5x5	3 pieces in a row wins. However, a player can only win by making a diagonal row. Horizontal and vertical rows do not count.	53.9	0.1	0.5	0.3
10x10	Each player needs 4 pieces in a row to win. The first player cannot win by making a diagonal row (only horizontal and vertical rows count), but the second player does not have this restriction.	52.9	-0.2	0.4	0.2
4x9	4 pieces in a row wins.	52.8	0.0	0.5	0.2
5x5	Each player needs 3 pieces in a row to win. The first player can only win by making a diagonal row, but the second player does not have this restriction.	52.6	0.1	0.2	0.5
10x10	6 pieces in a row wins.	51.8	0.0	0.7	0.2
10x10	3 pieces in a row wins. However, a player cannot win by making a diagonal row. Only horizontal and vertical rows count.	51.4	0.5	0.2	0.7
3x3	3 pieces in a row wins.	51.4	0.1	0.7	0.2
3x5	3 pieces in a row wins.	51.1	0.2	0.6	0.3
7x7	4 pieces in a row wins. However, a player cannot win by making a diagonal row. Only horizontal and vertical rows count.	50.9	0.1	0.5	0.3
4x6	4 pieces in a row wins.	50.6	0.1	0.7	0.2
7x7	4 pieces in a row wins. The second player can place 2 pieces as their first move, while the first player can only place 1 piece as their first move.	50.5	-0.0	0.4	0.3
10x10	Each player needs 5 pieces in a row to win. The first player can only win by making a diagonal row, but the second player does not have this restriction.	50.3	-0.2	0.4	0.2
3x3	3 pieces in a row wins. The second player can place 2 pieces as their first move, while the first player can only place 1 piece as their first move.	50.1	-0.2	0.5	0.2

Board	Rules	Fun	Payoff	P(Draw)	P(P1 Wins)
4x4	3 pieces in a row wins. However, a player cannot win by making a diagonal row. Only horizontal and vertical rows count.	50.0	0.4	0.3	0.5
10x10	8 pieces in a row wins.	49.2	0.0	0.7	0.2
4x4	Each player needs 3 pieces in a row to win. The first player cannot win by making a diagonal row (only horizontal and vertical rows count), but the second player does not have this restriction.	49.0	-0.1	0.4	0.3
5x5	Each player needs 4 pieces in a row to win. The first player cannot win by making a diagonal row (only horizontal and vertical rows count), but the second player does not have this restriction.	48.6	-0.0	0.7	0.2
10x10	Each player needs 3 pieces in a row to win. The first player can only win by making a diagonal row, but the second player does not have this restriction.	48.2	0.0	0.2	0.4
5x5	4 pieces in a row wins. The second player can place 2 pieces as their first move, while the first player can only place 1 piece as their first move.	46.0	-0.1	0.7	0.1
5x5	5 pieces in a row wins. The second player can place 2 pieces as their first move, while the first player can only place 1 piece as their first move.	45.9	-0.1	0.7	0.1
7x7	Each player needs 4 pieces in a row to win. The first player can only win by making a diagonal row, but the second player does not have this restriction.	45.6	-0.3	0.5	0.1
10x10	3 pieces in a row wins. However, a player can only win by making a diagonal row. Horizontal and vertical rows do not count.	44.9	0.3	0.4	0.5
3x10	3 pieces in a row wins.	44.5	0.4	0.3	0.5
10x10	4 pieces in a row loses.	44.4	0.0	0.5	0.3
5x5	The first player needs 5 pieces in a row to win, but the second player only needs 4 pieces in a row to win.	44.4	-0.2	0.5	0.1
3x3	3 pieces in a row loses.	43.2	-0.0	0.8	0.1
5x5	5 pieces in a row wins. However, a player cannot win by making a diagonal row. Only horizontal and vertical rows count.	41.7	0.0	0.9	0.0
8x8	3 pieces in a row wins.	41.3	0.5	0.2	0.7
10x10	The first player needs 10 pieces in a row to win, but the second player only needs 9 pieces in a row to win.	41.2	-0.0	0.7	0.1
4x4	3 pieces in a row wins. However, a player can only win by making a diagonal row. Horizontal and vertical rows do not count.	41.2	0.1	0.8	0.1
10x10	7 pieces in a row wins.	40.9	0.1	0.7	0.2
10x10	Each player needs 4 pieces in a row to win. The first player can only win by making a diagonal row, but the second player does not have this restriction.	40.9	-0.2	0.4	0.2

Board	Rules	Fun	Payoff	P(Draw)	P(P1 Wins)
5x5	Each player needs 3 pieces in a row to win. The first player cannot win by making a diagonal row (only horizontal and vertical rows count), but the second player does not have this restriction.	40.6	-0.0	0.4	0.3
InfxInf	3 pieces in a row wins.	40.1	0.6	0.2	0.7
10x10	10 pieces in a row wins. However, a player cannot win by making a diagonal row. Only horizontal and vertical rows count.	40.1	0.0	0.8	0.1
4x6	3 pieces in a row wins.	39.7	0.6	0.2	0.7
10x10	3 pieces in a row wins.	39.2	0.6	0.1	0.7
7x7	The first player needs 4 pieces in a row to win, but the second player only needs 3 pieces in a row to win.	39.1	-0.6	0.2	0.1
4x12	5 pieces in a row wins.	38.3	0.0	0.8	0.1
10x10	The first player needs 5 pieces in a row to win, but the second player only needs 4 pieces in a row to win.	38.1	-0.1	0.3	0.3
9x9	3 pieces in a row wins.	37.9	0.6	0.1	0.7
5x5	5 pieces in a row loses.	37.2	0.0	0.7	0.1
10x10	The first player needs 4 pieces in a row to win, but the second player only needs 3 pieces in a row to win.	37.2	-0.4	0.2	0.2
10x10	10 pieces in a row wins. The first player can place 2 pieces as their first move, while the second player can only place 1 piece as their first move.	36.8	0.1	0.7	0.2
7x7	3 pieces in a row wins.	36.4	0.3	0.3	0.5
10x10	10 pieces in a row wins. The second player can place 2 pieces as their first move, while the first player can only place 1 piece as their first move.	36.2	0.0	0.6	0.2
5x5	Each player needs 5 pieces in a row to win. The first player cannot win by making a diagonal row (only horizontal and vertical rows count), but the second player does not have this restriction.	35.7	-0.1	0.6	0.1
10x10	9 pieces in a row wins.	35.6	0.0	0.8	0.1
5x5	Each player needs 4 pieces in a row to win. The first player can only win by making a diagonal row, but the second player does not have this restriction.	35.4	-0.3	0.6	0.1
5x5	5 pieces in a row wins.	35.4	0.1	0.7	0.2
3x3	3 pieces in a row wins. However, a player cannot win by making a diagonal row. Only horizontal and vertical rows count.	35.0	0.0	0.9	0.1
5x5	4 pieces in a row wins. However, a player can only win by making a diagonal row. Horizontal and vertical rows do not count.	34.8	0.0	0.8	0.1
4x4	Each player needs 3 pieces in a row to win. The first player can only win by making a diagonal row, but the second player does not have this restriction.	34.0	-0.3	0.6	0.1

Board	Rules	Fun	Payoff	P(Draw)	P(P1 Wins)
10x10	Each player needs 3 pieces in a row to win. The first player cannot win by making a diagonal row (only horizontal and vertical rows count), but the second player does not have this restriction.	33.9	0.3	0.2	0.5
5x10	6 pieces in a row wins.	33.9	0.0	0.7	0.2
InfxInf	10 pieces in a row wins.	33.9	0.1	0.5	0.3
5x5	5 pieces in a row wins. The first player can place 2 pieces as their first move, while the second player can only place 1 piece as their first move.	33.8	0.0	0.8	0.1
10x10	Each player needs 10 pieces in a row to win. The first player cannot win by making a diagonal row (only horizontal and vertical rows count), but the second player does not have this restriction.	32.9	-0.0	0.8	0.1
6x6	3 pieces in a row wins.	32.4	0.4	0.3	0.5
10x10	10 pieces in a row loses.	32.3	-0.0	0.9	0.0
5x5	3 pieces in a row wins. The second player can place 2 pieces as their first move, while the first player can only place 1 piece as their first move.	32.2	-0.4	0.3	0.1
2x10	3 pieces in a row wins.	32.0	0.1	0.7	0.2
4x4	3 pieces in a row wins. The first player can place 2 pieces as their first move, while the second player can only place 1 piece as their first move.	32.0	0.7	0.1	0.8
10x10	3 pieces in a row wins. The second player can place 2 pieces as their first move, while the first player can only place 1 piece as their first move.	31.8	-0.3	0.3	0.2
3x3	Each player needs 3 pieces in a row to win. The first player cannot win by making a diagonal row (only horizontal and vertical rows count), but the second player does not have this restriction.	30.3	-0.0	0.7	0.1
4x9	5 pieces in a row wins.	29.9	0.0	0.8	0.1
5x5	4 pieces in a row wins. However, a player cannot win by making a diagonal row. Only horizontal and vertical rows count.	29.2	0.1	0.6	0.2
4x6	5 pieces in a row wins.	29.0	-0.0	0.9	0.0
10x10	3 pieces in a row wins. The first player can place 2 pieces as their first move, while the second player can only place 1 piece as their first move.	28.8	0.8	0.1	0.8
4x4	3 pieces in a row wins. The second player can place 2 pieces as their first move, while the first player can only place 1 piece as their first move.	28.1	-0.5	0.3	0.1
10x10	10 pieces in a row wins.	26.1	0.0	0.9	0.1
5x5	Each player needs 5 pieces in a row to win. The first player can only win by making a diagonal row, but the second player does not have this restriction.	25.8	-0.1	0.7	0.1
3x3	3 pieces in a row wins. However, a player can only win by making a diagonal row. Horizontal and vertical rows do not count.	25.1	0.0	0.9	0.1

Board	Rules	Fun	Payoff	P(Draw)	P(P1 Wins)
5x5	5 pieces in a row wins. However, a player can only win by making a diagonal row. Horizontal and vertical rows do not count.	25.1	0.0	0.8	0.1
10x10	The first player needs 3 pieces in a row to win, but the second player only needs 2 pieces in a row to win.	24.7	-0.8	0.1	0.1
3x3	3 pieces in a row wins. The first player can place 2 pieces as their first move, while the second player can only place 1 piece as their first move.	24.6	0.5	0.3	0.6
4x4	The first player needs 3 pieces in a row to win, but the second player only needs 2 pieces in a row to win.	23.2	-0.6	0.1	0.2
3x3	2 pieces in a row wins.	22.5	0.8	0.1	0.8
3x3	Each player needs 3 pieces in a row to win. The first player can only win by making a diagonal row, but the second player does not have this restriction.	21.1	-0.2	0.7	0.0
5x5	3 pieces in a row wins. The first player can place 2 pieces as their first move, while the second player can only place 1 piece as their first move.	20.8	0.7	0.2	0.7
2x5	3 pieces in a row wins.	20.7	0.0	0.8	0.1
10x10	10 pieces in a row wins. However, a player can only win by making a diagonal row. Horizontal and vertical rows do not count.	20.4	0.0	0.7	0.1
10x10	Each player needs 10 pieces in a row to win. The first player can only win by making a diagonal row, but the second player does not have this restriction.	20.2	-0.2	0.6	0.1
3x3	The first player needs 3 pieces in a row to win, but the second player only needs 2 pieces in a row to win.	16.1	-0.7	0.1	0.1
1x10	3 pieces in a row wins.	14.4	0.0	0.8	0.1
1x5	3 pieces in a row wins.	11.8	0.0	0.9	0.1
10x10	2 pieces in a row wins.	11.8	0.9	0.0	1.0
5x5	The first player needs 3 pieces in a row to win, but the second player only needs 2 pieces in a row to win.	8.8	-0.7	0.1	0.1
1x5	2 pieces in a row wins.	8.5	0.6	0.3	0.6
5x5	2 pieces in a row wins.	6.5	1.0	0.0	1.0

References

[74] van Opheusden, B. *et al.* Expertise increases planning depth in human gameplay. *Nature* 1–6 (2023).

[75] Browne, C. B. *et al.* A survey of monte carlo tree search methods. *IEEE Transactions on Computational Intelligence and AI in Games* **4**, 1–43 (2012).

[76] OpenAI Team *et al.* OpenAI o1 System Card (2024). URL https://arxiv.org/abs/2412.16720. 2412.16720.

[77] Menéndez, M. L., Pardo, J. A., Pardo, L. & Pardo, M. d. C. The Jensen-Shannon divergence. *Journal of the Franklin Institute* **334**, 307–318 (1997).
