# Supplementary material for: People use fast and flat simulation to reason about new games
Source: Nature. 2026 Jul 15;655(8123):598–607. doi: 10.1038/s41586-026-10722-1 (PMC13372689; doi:10.1038/s41586-026-10722-1)
Supplement: Supplementary file 4 — Peer Review File [file 41586_2026_10722_MOESM4_ESM.pdf]

nature portfolio

Peer Review File

People use fast and flat simulation to reason about new games

Corresponding Author: Dr Katherine Collins

Any redactions in this file are there to maintain patient confidentiality, the confidentiality of unpublished data, or to remove third-party material.

This file contains all reviewer reports in order by version, followed by all author rebuttals in order by version.

Version 0:

Reviewer comments:

Referee #1

(Remarks to the Author)
This paper presents an investigation into human decision making through a large variation of simple combinatorial puzzles or two-player games akin to board games. Hypothesizing that human beings are systematic and adaptively rational in how they play a game with rules they are not familiar with for the first time, the paper posits that human behavior in such situations can be partially explained through a cognitive model the authors name "The Intuitive Gamer".

The model is characterized by being a single-step look-ahead model with a sparse, shallow tree, supported by heuristics. Mapping experimental data from human decision makers, the paper shows that this model describes actual human decision making more accurately than a naive, random model or an Expert Gamer model with deeper search, and even more computation-heavy approaches such as Monte Carlo Tree Search. This conclusion is formed through the analysis of a large empirical dataset and suggests that the authors' model is more appropriate for describing human decision making in new/novel, to the decision maker, rule systems than a stochastic deep search-based model. Extrapolating from the findings in the paper, it may be reasonable to assume that humans, when met with other novel rule systems or decision making problems, make judgments in a similar manner - by extrapolating a few steps ahead a few branches to a few likely outcomes, with the branches investigated heavily informed by common sense or generalistic heuristics.

As such, the results could be interpreted to showcase an example of guided System 1/System 2 interaction in decision making when presented with new rule sets/decision making environments. The paper is well-written with a clear and coherent narrative and solid, comprehensive methodological work. Additionally, it is supported by a dataset of an impressive size for this kind of highly structured experiment.

I believe the main contribution of the paper is formulating a crisp hypothesis about decision making in structured, novel problems, exemplifying it with a range of reasonable models as explanatory candidates, and showcasing how the authors' model is the best candidate out of the selection.

There are a few points, that I would be interested in seeing expanded or clarified from the paper:

How did the authors come up with the three heuristics; were others considered and rejected? The three heuristics chosen - whether they progress the player's goal, block opponents, and provide centrality on the board (perhaps to offer optionality or empowerment in the sense of e.g. Polani & Salge) - all seem intuitively reasonable, but it would be helpful to understand if something other than intuition and their use i prior literature motivated them.

On the matter of whether the games are fun, It might be useful for the discussion to engage with some of the literature already in existence studying fun and appeal in board and card games, e.g. Lantz et al.'s paper on Depth in Strategic Games comes to mind. If the proposed grid based problems truly are posited as games (which I think is totally reasonable and fine) it would make sense to slightly expand to a broader engagement with the literature on board and card games, especially on the question of fun, which has been discussed at length in the game studies literature. I understand the paper acknowledges that fun is elusive and difficult to operationalize, but e.g. looking at how the PENS instrument (Ryan et al.) operationalizes

fun with five dimensions: competence, autonomy, relatedness, immersion/presence, and intuitive control. It would seem like what is being measured in this study is closest to concepts depth (Lantz) or competence (Ryan), and seems worthwhile to visit since the paper is already commenting on what fun is and how to measure it.

In the same vein, the paper in the discussion mentions how the findings might extend to provide partial explanation of cognition and behavior in novel areas, for instance scientific work. I think it is very reasonable to think that (some of) the same cognitive strategies applied when encountering new games are used in other novel problem contexts, but extrapolating strongly from a model that can describe intuitive reasoning in novel grid-based problems to these highly complex, situated, or embodied decision making contexts seems difficult, and it might be worth including a short examination of all the other aspects of experience, learning, play, and motivation that impact decision making in such situations. It would be unreasonable to ask for a full discussion of the decision science space here, but e.g. Klein's (~1998) work on recognition-primed decision making could be relevant, as well as Griffith's work on resource-rational cognition (e.g. ~2020) or Gigerenzer's work (e.g. 2001) might be worth mentioning and the findings of this study seem to align with the theories and findings of these bodies of work.

Here, one could also visit the foundational question of whether doing mental simulation in response to a novel problem before making decisions is even natural or typical human behavior at all or whether humans are more likely to learn iteratively in a multi-step, multi-session cyclical manner (as described in e.g. Kolb's Experiential Learning Theory, e.g. ~1984). I believe this ties into a characteristic of the models employed in the paper in that they treat each problem addressed by the participants as independent from the rest, which is reasonable to reduce complexity - but in reality must be considered likely that the participants are learning some sort of meta-information and applying it across individual problems.

The three perspectives/fields I list with examples above all provide related lenses on how humans actually do "zero-shot, zero-experience" problem solving. Since this paper sits at an intersection of cognitive modeling, decision making theory, and game theory, I would suggest briefly including all three areas in the discussion - I think it would help situate the contribution of the paper, and much of the work above aligns with the conclusion and extrapolations of the paper, albeit from slightly adjacent fields. The paper speaks broadly about a range of human domains of activity, so I think that motivates a broadening of the discussion.

(Remarks on code availability)

Referee #2

(Remarks to the Author)
Summary of the key results

This paper investigates how novice players reason when first learning to play board games and proposes computational models that predict their initial moves. The authors identify a research gap: prior work emphasizes optimal play, but there has been little quantitative modelling of novice behaviour. To study this, the paper constructs 121 Tic-Tac-Toe-style game variants by varying board size, play dynamics, and winning rules.

The proposed model, Intuitive Gamer, has two components:

* Player Module. Predicts a single move from a given game state using fast, one-step lookahead search over handcrafted evaluation functions. The module is designed to be (i) computationally light, (ii) flat (one-step search), (iii) goal-directed (optimizes win probability), and (iv) probabilistic: action selection follows a Boltzmann policy over a linear combination of action utilities.

* Reasoning Module. Estimates players' judgments about game properties by running a small number of simulated rollouts (self-play with the Player Module) and examining the resulting distributions over queried metrics. This module can produce game evaluations (e.g., balance, complexity) without requiring human play.

Two behavioural study suites evaluate the model. The first asks participants to judge unplayed games (fairness and fun) and compares human judgements to the Intuitive Gamer's predicted payoffs and to regressed values combining balance, reward thinking, and game length. The second compares human first moves directly against model predictions. Across experiments the Intuitive Gamer is compared to three baselines: an Expert Game (a deeper variant of the Intuitive Gamer), Monte Carlo Tree Search (MCTS), and a Random policy.

Originality and significance

Modelling novice reasoning in games is an important and underexplored area. While optimal play (e.g., Chess, Go) has been extensively studied and has produced powerful methods such as MCTS-based systems, there is comparatively little work that explicitly models early, novice reasoning. The paper's central idea (designing a lightweight, psychologically informed model to capture early, "intuitive" decisions) is timely and has the potential to complement research on expert play by filling the gap at the start of the skill training.

The paper's behavioural studies demonstrate that Intuitive Gamer aligns reasonably well with human judgments and first-move choices in Tic-Tac-Toe-like games. If these results generalize beyond the tested family, the approach could inform (a)

the design of heuristics and value functions used inside search methods such as MCTS when initial (novice-like) behaviour is relevant, and (b) design principles for game designers interested in creating games that feel "fair" or "fun" to new players.

Data & methodology

The Intuitive Gamer combines handcrafted state-evaluation functions with a one-step tree search and Monte Carlo rollouts. Specifically:

* The Player Module performs a single-step look-ahead and evaluates actions using a weighted sum of utility components; it then samples moves with a Boltzmann policy. This resembles the selection/expansion phase of MCTS but constrained to depth-one and using handcrafted utilities rather than learned value nets.

* The Reasoning Module uses a small number of self-play rollouts (by the Player Module) to estimate distributions over game metrics. Methodologically, this is analogous to MCTS simulation, but the simulations are short and use the lighter Player Module policy.

This design makes Intuitive Gamer computationally cheaper and psychologically plausible for modelling fast, shallow human reasoning. However, because it is closely related to MCTS (and can be viewed as a shallow MCTS with specific handcrafted heuristics), the paper should more clearly articulate what distinguishes the contribution in methodology, as "the computation account of how people reason" was claimed as the core contribution by the paper.

Suggested improvements

1. Limit grand claims in the introduction / tighten scope.
The introduction references a wide range of games (e.g., Mancala, Chess, Go) but experiments focus on Tic-Tac-Toe variants. Reframe the introduction to foreground Tic-Tac-Toe-style games as the study domain and justify why these variants are an appropriate experimental testbed for novice reasoning. If generalization beyond this family is claimed, provide clearer argumentation and/or preliminary evidence.

2. Clarify and motivate the utility decomposition; discuss generalizability.
The Player Module's action value is a sum of three utilities (e.g., U_self, U_opp, U_aux). Explain why this particular decomposition was chosen, how sensitive results are to the linear combination, and whether alternative decompositions were considered (e.g., data-driven learning-based decompositions). Discuss explicit limitations for transferring these handcrafted utilities to games like Go or Chess and propose how the framework could be adapted (e.g., by learning weights or feature transforms, or by replacing contiguous-line features with more general connectivity/territory metrics).

3. Position relative to MCTS more explicitly.
Intuitive Gamer may be read as a constrained MCTS variant (depth-one search + handcrafted evaluation + short simulations). The paper should (a) clearly state the similarities and differences to MCTS, (b) explain why a dedicated model is warranted for novice reasoning (or why the existing model can't be applied if the search depth is limited and value functions are heuristic-based), and (c) provide experiments or analysis showing where Intuitive Gamer better matches human data than a not-tuned shallow MCTS with the same action selection policy (e.g., MCTS configured with one-step rollout and Boltzmann selection, but with the same value functions as in Intuitive Gamer).

4. Explain practical computation details for the Reasoning Module.
Provide explicit pseudocode or complexity analysis: how many rollouts are used, how rollout length and sample size affect the estimates, what it means "the simulation ... may terminate early with some probability distribution on stopping times", and how "Computations over the simulated gameplay traces then inform the resulting distribution over the query values" (how to inform?). This will help readers evaluate the claim that the module "can evaluate games without ever playing" while remaining efficient.

5. Report participant novelty controls and provide more participant detail.
Clarify how "novice" status was enforced or assessed in the Prolific study (screening questions, self-reports, prior experience checks, or exclusion criteria). If such controls were not used, report participants' prior exposure to Tic-Tac-Toe and the variants so readers can judge whether the cohort was truly novice.

Clarity and questions for the authors

1. Self-play in asymmetric games. How is self-play implemented for instances where player roles differ (different dynamics or winning rules for player 1 vs player 2)? Are both roles simulated and aggregated, or is a single-role simulation used?

2. Center definition for U_aux. How is the board center computed on non square boards (e.g., 4×6) and on variant board topologies? Was any normalization applied across board sizes when using Euclidean distance?

3. Equation (3) correction. Equation (3) appears to be missing a term on its first right-hand side. Please confirm the intended formulation and update the manuscript.

4. Please clarify the necessity of a separate Intuitive Gamer model (since it's a combination of value functions and tree

search). Could a shallow, lightly-tuned MCTS with a Boltzmann action policy and simple handcrafted heuristics replicate the Intuitive Gamer's behaviour? If not, present evidence (or an argument) showing which element in the Intuitive Gamer's architecture is necessary to capture the behavioural signatures observed (i.e., the fundamental difference in the model)

(Remarks on code availability)
The provided code package includes a comprehensive README file containing installation instructions, directory structure descriptions, and information on the human participants' data. A requirements.txt file is also included, clearly specifying all necessary Python modules and their corresponding versions. Most of the experimental results are stored in CSV and JSON formats. (Note: I did not install or execute the code myself.)

There are, however, a few minor issues worth noting:

* The directory related to "funness" (Reasoning Module) appears to be incomplete: it contains only an __init__.py file without any implementation scripts.

* The codebase includes an llm component that appears to provide LLM-based baselines and analyses related to game funness. However, these elements are not described or discussed in either the main paper or the appendix, leaving their purpose and relevance unclear.

Referee #3

(Remarks to the Author)
This is a highly original and elegant piece of work focusing on building a model of intuitive gameplay: what predicts whether people think a (novel) game is fun; whether to accept a draw; which move to make? The authors develop an "Intuitive Gamer" model and pit it against both simpler and more complex models.

I found the motivations and execution of the project to be compelling and have no major concerns (though I admit to only a cursory read through the supplementary materials). There is an abundance of intriguing results in the supp materials that are not discussed in the main manuscript, but which offers many opportunities for follow-ups (e.g., in what ways are LLMs aligning with and departing from the intuitive gamer model?).

(1)
My main concern with the manuscript in its present form is that the model is pitched as a model of games (the authors do acknowledge that they hope there will be followup work to extend it to other types of games). My worry is that as implemented the model is *too* specific to tic-tac-toe style games. Is there any reason to think the present model is the right one for board games more generally? For card games? For physical games? The reason it matters is that while the difference between a beginner and an expert in a tic-tac-toe style game may be well captured by depth of search, in many games experts may be using completely different sources of information compared to beginners. For example, a beginning poker player may not even be considering the social dimension of the game. A beginning tennis player may be focused on just returning the ball and is just unaware of most of the factors that characterize expert play.

The concrete request is to acknowledge the limitations more explciitly. What types of games do you think this model applies to?

(2)
In my judgment, the present visualizations are toofocused on showing model fits, and do not effectively convey the patterns in the human data. Which games are judged to be most and least fun? Which factors are most predictive? (ditto for some of the other factors like likelihood of a draw and the advantage of going first).

Other comments:

Please add color legens to the figures where applicable so that the reader doesn't have to read through the whole caption or refer to other figures to understand the color mapping.

Figure 1 caption: "how many pieces a any player can make" (can make?)

(Remarks on code availability)

Version 1:

Reviewer comments:

Referee #2

(Remarks to the Author)
The reviewer thanks the authors for the detailed and constructive response. The revisions substantially improve clarity and address most of my concerns.

1. Behavioural Studies
The clarification that participants played full matches (not only first moves), and that action prediction covered early, mid, and late stages, resolves a key misunderstanding. The clearer description of the three study types strengthens the empirical validation.

2. Positioning Relative to MCTS
The added subsection comparing Intuitive Gamer and MCTS improves clarity. However, I remain partially unconvinced that the distinction is entirely conceptual rather than parametric. While the authors argue that “any stochastic tree search” could resemble MCTS at a high level, my original suggestion was more specific: to empirically compare against a deliberately constrained MCTS configured with the same heuristics and action policy. I think the paper may explicitly acknowledge that the boundary between “shallow MCTS” and “Intuitive Gamer” is partly architectural emphasis rather than algorithmic novelty and that the contribution lies in the cognitive interpretation and modular framing rather than proposing a fundamentally new search algorithm, i.e., intuitive gamer.

3. Utility Decomposition
The grounding of progress/blocking/cantering in prior literature and the sensitivity analyses clarify motivation. However, I do not find the exclusion of learning-based decompositions fully convincing. Even under a zero-experience framing, one could test features learned across related games (but not the specific instance) to assess whether handcrafted heuristics are uniquely necessary. Without such a comparison, it remains unclear whether the proposed decomposition reflects psychologically grounded priors or modelling convenience. This should be either better justified or explicitly acknowledged as a limitation.

4. Reasoning Module & Computation
The addition of pseudocode and clearer explanations significantly improves transparency.

5. Novice Status
Supplemented participant experience statistics are helpful. However, post-survey self-reports of prior experience may not be very rigorous and can be vulnerable to bias. If the novice framing is central, stronger controls (e.g., pre-screening or exclusion criteria) are needed. Otherwise, I recommend careful phrasing: this reflects novelty within a familiar genre, not complete inexperience.

6. Component Necessity
The consolidated summary of model ablations (flat, fast, goal-directed, probabilistic) provides a clear theoretical account of what is necessary to match human behaviour.

7. Code & LLM Baselines
Repository restructuring clarifies earlier issues. The main text clarifying that LLM baselines are exploratory helps avoid confusion.

(Remarks on code availability)

Open Access This Peer Review File is licensed under a Creative Commons Attribution 4.0 International License, which permits use, sharing, adaptation, distribution and reproduction in any medium or format, as long as you give appropriate credit to the original author(s) and the source, provide a link to the Creative Commons license, and indicate if changes were made.
In cases where reviewers are anonymous, credit should be given to 'Anonymous Referee' and the source.
The images or other third party material in this Peer Review File are included in the article's Creative Commons license, unless indicated otherwise in a credit line to the material. If material is not included in the article's Creative Commons license and your intended use is not permitted by statutory regulation or exceeds the permitted use, you will need to obtain permission directly from the copyright holder.
To view a copy of this license, visit https://creativecommons.org/licenses/by/4.0/

Dear Dr. Sweeney,

We thank you and the reviewers for your time and thoughtful comments. We appreciate that the reviewers generally found our work original and elegant, both in the motivation of the work and our execution. We are especially glad that reviewers across such diverse disciplines (game AI, reinforcement learning, and cognitive science) found value in our work. We hope such cross-disciplinary excitement is a precursor for wider interest across the kind of diverse readership of *Nature*.

We have prepared a revision that addresses the key points requested by the reviewers and also significantly tightened the main text, as requested in the editorial comments. The main text (not including Methods, captions, or Supplement) is now approximately 5,457 words. This has primarily involved a restructure of our model section (with details now in Methods and better clarity on alternate models, per Reviewer 2's questions); substantially reducing the Introduction and fully-referenced first summary; and some tightening of Results. We think the shorter version is substantially clearer, and hope you and the Reviewers do too. However, in order to address the Reviewers' questions and concerns (particularly in the Discussion and description of the funness data and model in Results), we felt it was important to submit this version of our manuscript at slightly over 5k words. If the paper is accepted and further cuts are deemed necessary, of course we would be happy to make any further edits you request.

We have also trimmed the title to meet the character count (removing the "," and changing "novel" to "new"), reduced the number of references in the main text to the requested 50, and tightened our abstract to less than 230 words. And as recommended, we have also now annotated each main figure with a data file for reproducibility and uploaded our code to Zenodo (as described in our Code and Data Availability statements); we will also release a public GitHub repository containing code and data upon publication of the work.

Below, we provide point-by-point responses to each reviewer. Reviewer text is in black, our responses are in green.

Reviewer 1

We thank the Reviewer for the time and thoughtfulness they put into the review. We are glad they appreciated the work's core value proposition, and we particularly appreciated the additional related work they pointed us to that helped us better situate our work across fields. We now address point-by-point those aspects of the paper that they asked to be expanded upon or clarified:

“How did the authors come up with the three heuristics; were others considered and rejected? The three heuristics chosen - whether they progress the player’s goal, block opponents, and provide centrality on the board (perhaps to offer optionality or empowerment in the sense of e.g. Polani & Salge) - all seem intuitively reasonable, but it would be helpful to understand if something other than intuition and their use in prior literature motivated them.”

This is a reasonable question, and echoes points raised by Reviewer 2. The heuristics were indeed primarily motivated by prior literature backed by expert intuitions. As you indicate, concepts of progress and blocking are present in the classic study of Crowley and Siegler (1993), and similarly progress patterns and central tendencies are used in the recent work by Opheusden et al. (2023), on which we base our expert model. More generally, features based on connections (or lack thereof), implemented differently based on games, is pretty common in board game-related AI. Accordingly, our team (consisting of both cognitive scientists and expert game players) judged the combination of connection, blocking, and center-bias terms to be a reasonable set of heuristics for our collection of games. In addition, we considered subsets of these three heuristics, which led to the study in S.I. 3.3. We have better clarified the motivation for this utility decomposition in the Methods under "Intuitive Gamer player module” (starting on pg. 20).

“On the matter of whether the games are fun, It might be useful for the discussion to engage with some of the literature already in existence studying fun and appeal in board and card games, e.g. Lantz et al.’s paper on Depth in Strategic Games comes to mind. If the proposed grid based problems truly are posited as games (which I think is totally reasonable and fine) it would make sense to slightly expand to a broader engagement with the literature on board and card games, especially on the question of fun, which has been discussed at length in the game studies literature. I understand the paper acknowledges that fun is elusive and difficult to operationalize, but e.g. looking at how the PENS instrument (Ryan et al.) operationalizes fun with five dimensions: competence, autonomy, relatedness, immersion/presence, and intuitive control. It would seem like what is being measured in this study is closest to concepts depth (Lantz) or competence (Ryan), and seems worthwhile to visit since the paper is already commenting on what fun is and how to measure it.”

Thank you for the suggestion and the pointers to important related work. We have revised our Discussion with a new paragraph (pg. 13) that highlights the connections of our funness model to the literature you cite (and several other related works), but also marks an important contrast: We are modeling people's intuitive theories of funness and how those guide their judgments about what they expect will be fun or worth doing before they even try a game, rather than measuring or theorizing about what people will actually experience as fun when they play a game. We also note that future work should investigate whether other dimensions of the PENS instrument may

have correlates in people's intuitive theories of fun in games, and whether the Intuitive Gamer could be extended to model them.

“In the same vein, the paper in the discussion mentions how the findings might extend to provide partial explanation of cognition and behavior in novel areas, for instance scientific work. I think it is very reasonable to think that (some of) the same cognitive strategies applied when encountering new games are used in other novel problem contexts, but extrapolating strongly from a model that can describe intuitive reasoning in novel grid-based problems to these highly complex, situated, or embodied decision making contexts seems difficult, and it might be worth including a short examination of all the other aspects of experience, learning, play, and motivation that impact decision making in such situations. It would be unreasonable to ask for a full discussion of the decision science space here, but e.g. Klein’s (~1998) work on recognition-primed decision making could be relevant, as well as Griffith’s work on resource-rational cognition (e.g. ~2020) or Gigerenzer’s work (e.g. 2001) might be worth mentioning and the findings of this study seem to align with the theories and findings of these bodies of work.”

Thank you for the comments. We agree with your perspective here and have made targeted changes to our Discussion in response. We have kept these changes short, however, and we hope the Reviewer will understand, bearing in mind that space is very much at a premium especially with the Editor's request to substantially reduce the overall length of our Main Text.

Specifically, we agree that work from Klein, Griffiths, Gigerenzer and their colleagues is relevant and making these connections would help to show both how our work aligns with other research in the decision science space, as well as how ideas from that space could be valuable in extending and deepening our account. We have now included these references along with brief notes to this effect in the Discussion paragraph on p. 13 that begins "*Additionally, there is a need for more fine-grained process-level and individual-level accounts of how people reason about novel games.*"

We also agree that the connections to scientific reasoning raised at the end of our Discussion extrapolate strongly from the model we have presented so far, and that this should be marked more clearly, as well as qualified to note that our work is relevant (at best) to only some aspects of scientific thinking. Our goal here was to highlight how our modeling could form the basis for a more general class of models that extends to other kinds of problems and systems of rules and reward (games, broadly construed), using science as an example of a “game against nature.” We intended for that paragraph to be more speculative and inspire a general reader of *Nature*. We have now changed the text (pg. 14) to make clear that we are intending the ending to be speculative, by beginning our key sentence with "Suppose, more speculatively, that …" We have also clarified that our model is intended not as a full explanation here, but that it could "help to

explain aspects of" how scientists choose problems to think about or how to approach them. We hope that these changes, along with the additional references to the larger decision science space which we have added, better contextualizes the claims and contributions we can make. And we are grateful to the Reviewer for helping to strengthen the paper in these ways.

“one could also visit the foundational question of whether doing mental simulation in response to a novel problem before making decisions is even natural or typical human behavior at all or whether humans are more likely to learn iteratively in a multi-step, multi-session cyclical manner (as described in e.g. Kolb’s Experiential Learning Theory, e.g. ~1984).”

Excellent point. Based on your suggestion, we have incorporated a pointer to connecting to experiential learning in future work in our Discussion (pg. 13).

“in the paper in that they treat each problem addressed by the participants as independent from the rest, which is reasonable to reduce complexity - but in reality must be considered likely that the participants are learning some sort of meta-information and applying it across individual problems.”

Indeed, we assume people approach each game as if it is a new game (not re-using any computation from past games). However, as you note, it is possible that just as people learn within a game, they may learn across games. We think this is ripe ground for future work. We have clarified this assumption of independence in the model section (pg. 4) and noted in the Discussion (pg. 13) the potential for future investigation here (and situating relative to other work around computation reuse, e.g., Dasgupta et al, 2018).

“Since this paper sits at an intersection of cognitive modeling, decision making theory, and game theory, I would suggest briefly including all three areas in the discussion - I think it would help situate the contribution of the paper, and much of the work above aligns with the conclusion and extrapolations of the paper, albeit from slightly adjacent fields.”

Thank you for this suggestion and we agree that our work's connections to all these fields is central to its contributions. Based on your comments, we have explicitly mentioned all three areas in the Discussion, and we have made reference to the connections the work may have in other fields such as economics (pg. 13). We hope the work will appeal and directly connect to work amongst the broad readership of *Nature*.

Reviewer 2

We thank the Reviewer for their thinking and questions! We appreciate that they found our work important and timely — quoting from the "Originality and significance" section of the review:

"Modelling novice reasoning in games is an important and underexplored area. While optimal play (e.g., Chess, Go) has been extensively studied and has produced powerful methods such as MCTS-based systems, there is comparatively little work that explicitly models early, novice reasoning. The paper's central idea (designing a lightweight, psychologically informed model to capture early, "intuitive" decisions) is timely and has the potential to complement research on expert play by filling the gap at the start of the skill training."

Below we respond point-by-point in order to all comments, questions and recommendations, beginning after the first few paragraphs summarizing the paper and our modeling. We start with the paragraphs on our behavioral studies (first mentioned at the end of the Review's "Summary of the Key Results" section) where we believe there may have been some misunderstandings about the experiments we ran.

"Two behavioural study suites evaluate the model. The first asks participants to judge unplayed games (fairness and fun) and compares human judgements to the Intuitive Gamer's predicted payoffs and to regressed values combining balance, reward thinking, and game length. The second compares human first moves directly against model predictions. Across experiments the Intuitive Gamer is compared to three baselines: an Expert Game (a deeper variant of the Intuitive Gamer), Monte Carlo Tree Search (MCTS), and a Random policy."

The Reviewer writes that “Two behavioural study suites evaluate the model.” As shown in Figure 1, we ran *three* distinct studies, with people evaluating games before any play, people playing games, and people predicting actions others should take from watching their play. We compared model and human predictions for all studies. The first study had two substudies: one for predicting payoff/fairness and the other for predicting funness. We have now clarified this in the Main Text (p. 2).

The Reviewer also writes that “the second [study] compares human first moves directly against model predictions” and later mentions (under "Originality and significance") that we compare “first-move choices”:

[...] The paper's behavioural studies demonstrate that Intuitive Gamer aligns reasonably well with human judgments and first-move choices in Tic-Tac-Toe-like games. If these results generalize beyond the tested family, the approach could inform (a) the design of heuristics and value functions used inside search methods such as MCTS when initial (novice-like) behaviour is relevant, and (b) design principles for game designers interested in creating games that feel "fair" or "fun" to new players.

We would like to clarify that the participants in the gameplay experiment played through entire matches. We compared model and human predictions on *all moves*, not just the first move. And even in the action prediction (watch-and-predict) experiments, participants (and models) were assessed at their prediction on an early-stage, mid-stage, and late-stage move during matches; not the first move only. We have also clarified this in the Results and Methods (pgs. 10 and 26).

We regret that these points were not as evident before and we hope that the revision better shows the breadth and depth of our human studies. The capacity for our Intuitive Gamer model to capture human judgements across such a diverse range of behavioral tasks and games is a key contribution of the work — and one that we think sets it apart from past work (which often looks just at gameplay; or just at one game).

We also appreciate the recognition that our work is significant potentially for game design and AI. We believe our work is, at its heart, a contribution to the study of people—to cognitive science and computational models of cognition—but we agree it has implications for AI, game theory and decision making, as well as game design and other fields (as also noted by Reviewer 1).

"## Data & methodology

"The Intuitive Gamer combines handcrafted state-evaluation functions with a one-step tree search and Monte Carlo rollouts. Specifically:

* The Player Module performs a single-step look-ahead and evaluates actions using a weighted sum of utility components; it then samples moves with a Boltzmann policy. This resembles the selection/expansion phase of MCTS but constrained to depth-one and using handcrafted utilities rather than learned value nets.

* The Reasoning Module uses a small number of self-play rollouts (by the Player Module) to estimate distributions over game metrics. Methodologically, this is analogous to MCTS simulation, but the simulations are short and use the lighter Player Module policy.

This design makes Intuitive Gamer computationally cheaper and psychologically plausible for modelling fast, shallow human reasoning. However, because it is closely related to MCTS (and can be viewed as a shallow MCTS with specific handcrafted heuristics), the paper should more clearly articulate what distinguishes the contribution in methodology, as "the computation account of how people reason" was claimed as the core contribution by the paper."

Both above and in several places later on, the Reviewer asks us to discuss how our work is related to Monte Carlo Tree Search (MCTS)—the planning algorithm behind many AI systems

that achieve expert-level gameplay such as AlphaGo and AlphaZero—and what distinctive contribution we offer as a model of human reasoning.

We agree there are important points to clarify here, about why we did not frame our Intuitive Gamer in the way the Reviewer does and why we think it would be misleading or confusing to present it as "shallow MCTS". We also now see that clarifying this will better highlight our work's distinctive contribution as a computational account of human reasoning.

First, while we recognize there is some high-level resemblance between the Intuitive Gamer's play and restricted or shallow variants of MCTS, such a connection is so general as to capture almost any kind of stochastic tree search. (The same could be said of the Random Gamer agent, for example.) While this could potentially be discussed in the paper, we don't see it as the most valuable use of precious space.

We also worry that trying to make this connection could be confusing to many readers. The most distinctive features of the Intuitive Gamer model as a cognitive model are not what it has in common with MCTS, but how it departs from MCTS or any general-purpose tree-search-based algorithm that can approach the game play of strong human experts. MCTS made such a big impact on AI gameplay and reinforcement learning precisely because it could achieve very strong play without the need for heuristics to guide search. Instead, it uses extensive inference-time computation and sophisticated graph traversal arithmetic (backtracking, node visit counting) to efficiently explore a very large, unstructured game tree. This is in contrast to the Intuitive Gamer, which does not require any sophisticated algorithms and uses very minimal computation, although it *does* rely on a small number of abstract heuristics when assessing the value of next moves.

Such a design leads the Intuitive Gamer to be a highly efficient if less than optimal player, and one which we believe better captures how people reason (as evidenced in our behavioral action selection and action prediction studies). Indeed, as described in the “Resource-rational reasoning” section of the Main Text and captured in Extended Data Table 2, the Intuitive Gamer is **orders of magnitude** more efficient than either MCTS (run for 5k iterations) or what we call the Expert Gamer model, in terms of wallclock time and number of board states evaluated.

Recognizing that these points of contrast between the Intuitive Gamer and MCTS might not have been as clear as they could be, we have now marked them briefly but saliently on pg. 24 of the revised paper in the “Methods” in a new subsection “Comparison of MCTS and Intuitive Gamer”, and inserted a pointer to this subsection in the Main Text under “Alternative Models” (pg. 4-5) where we first mention MCTS as a comparison model.

A final set of considerations went into why we felt it was important to give a distinct name for our model, the Intuitive Gamer. The model we propose is more than just a model of how an agent chooses actions when playing a game, as MCTS is, and as most of gameplay-related AI and cognitive science has focused on. Critically, the Intuitive Gamer spans both the player module (for choosing actions in games) and a reasoning module that can answer questions about game play (e.g., who is likely to win, or how likely is a game to end in a win at all) by setting up and running simulations using the player module. This factorization into modules is crucial for how the model captures the way people think about games (or problems more generally) when confronted with them for the first time, before any experience. We choose to call the integrated system an "Intuitive Gamer", in contrast to the "Expert Gamer" (which is also more MCTS-like) to highlight its focus on how people think about games for the first time *before* developing expertise. This naming choice also connects to the wide body of literature in cognitive science around intuitive theories (e.g., intuitive physics, intuitive psychology, etc).

"## Suggested improvements"

"1. Limit grand claims in the introduction / tighten scope.
The introduction references a wide range of games (e.g., Mancala, Chess, Go) but experiments focus on Tic-Tac-Toe variants. Reframe the introduction to foreground Tic-Tac-Toe-style games as the study domain and justify why these variants are an appropriate experimental testbed for novice reasoning. If generalization beyond this family is claimed, provide clearer argumentation and/or preliminary evidence."

We have now substantially tightened the introduction, per your feedback and editorial requests.

We appreciate the need to be clearer about what class of games our model applies to. In response to your and Reviewer 3's similar comment, we have clarified in the Introduction that we consider a subclass of games that includes Tic-Tac-Toe and Connect-4, and devoted a focused paragraph of the Discussion to the question of game generality. We also now include a full listing of games in the Supplement.

We do want to note that even though the games could be seen as variants of Tic-Tac-Toe or more broadly "Connect-N" games, they do span quite a wide range of mechanics that lead to rich strategic behavior (see Figure 1a, Figure 4a, and Extended Data Table 1). They also capture many hallmarks of real-world decision making and planning problems.Some games are complex enough that there is not even a known game-theoretic optimal value. Moreover, even though we focus on a single class of two-player competitive strategy games here, we do consider many games within that class – in contrast to many works in AI and psychology which focus on just one game (e.g., Go, Chess, Diplomacy, etc). We see value in having a somewhat constrained yet

still rich testbed, where already there are substantial insights to be gleaned into the ways people (and models) evaluate these games before any play.

Nonetheless, we also make clearer now in the Discussion that our work takes just a first step towards the general problem of novel game evaluation, and there is much more to be done across a wider range of game classes.

2. Clarify and motivate the utility decomposition; discuss generalizability.
The Player Module's action value is a sum of three utilities (e.g., U_self, U_opp, U_aux). Explain why this particular decomposition was chosen, how sensitive results are to the linear combination, and whether alternative decompositions were considered (e.g., data-driven learning-based decompositions).

Thank you for raising this question (Reviewer 1 similarly asked about the heuristics); we respond similarly. The utility components are based on prior literature grounding and expert intuitions. Concepts of progress and blocking are present in the classic study of Crowley and Siegler (1993), and similarly progress patterns and central tendencies are used in the recent Opheusden et al. (2023), on which we base our expert model. More generally, features based on connections (or lack thereof), implemented differently based on games, is generally common in game-playing AI. Most of the authors are expert cognitive modelers and some are expert board game players (and played many Connect-N style games), so the combination of progress, blocking, and centering is effectively the first modeling hypothesis that we came up with and judged to be plausible. At the same time, we did consider what happens if we only include a subset of them or change the linear weights, which led to the study in SI 3.3. We have better clarified the motivation for this utility decomposition in the Methods under "Intuitive Gamer player module" (starting on pg. 20).

We also note that we did not consider learning-based decompositions in this work, as a hallmark of our current model is that it can capture how people reason about games *prior* to any experience. The question of how heuristics may be adapted with learning, however, is a very good one and an important next step off of this work. We have included a brief discussion of the importance of studying experiential learning (see response to Reviewer 1) in the Discussion (p. 13).

Discuss explicit limitations for transferring these handcrafted utilities to games like Go or Chess and propose how the framework could be adapted (e.g., by learning weights or feature transforms, or by replacing contiguous-line features with more general connectivity/territory metrics).

Based on your and Reviewer 3's comments on generalizability, we have added a clearer discussion of limitations and open work to assess generalizability to the Discussion (pg. 13). Given tight space constraints, we could not include a more complete discussion of how the framework specifically be adapted to other games; however, we include some possibilities here. While we hypothesize that the higher-level structure of the utility function we consider (i.e., a function which tries to make progress towards a player's goal and block their opponent's progress) would likely extend to other two-player perfect information competitive games, like Chess and Go, the actual lower-level heuristics (e.g. contiguous pieces in a line) would likely not. Both games are highly complex, and players are more likely to generate and adapt new heuristics while playing. One ongoing line of work we are exploring is language-guided automatic heuristic synthesis. There is already exciting work in this space from AI researchers, e.g., Lehrach et al, 2025's "Code World Models for General Game Playing;" however, the question of what heuristics a novice human reasoner employs when encountering a game for the very first time remains a ripe avenue for future work.

3. Position relative to MCTS more explicitly.
Intuitive Gamer may be read as a constrained MCTS variant (depth-one search + handcrafted evaluation + short simulations). The paper should (a) clearly state the similarities and differences to MCTS, (b) explain why a dedicated model is warranted for novice reasoning (or why the existing model can't be applied if the search depth is limited and value functions are heuristic-based), and (c) provide experiments or analysis showing where Intuitive Gamer better matches human data than a not-tuned shallow MCTS with the same action selection policy (e.g., MCTS configured with one-step rollout and Boltzmann selection, but with the same value functions as in Intuitive Gamer).

To the Reviewer's points (a) and (b), we hope these are addressed above in our long response to the first MCTS comment. While the Intuitive Gamer could in some way be recast as a variant of MCTS – it would only be in the sense that any stochastic tree search algorithm is, and it would be misleading as the features that make the Intuitive Gamer valuable as a cognitive model are precisely the opposite of the features that make MCTS so valuable in AI: it requires none of MCTS's sophisticated algorithms or score counting, and thus comes to approximate but reasonable judgements orders of magnitude more efficiently (see above and "Resource-rational reasoning"). We also reiterate that the simulations in the Intuitive Gamer are to answer general queries about the game, e.g., the expected outcomes, unlike MCTS simulations used to decide what action to take.

For these reasons, to point (c), we are unsure how we could improve the paper by trying to empirically distinguish the Intuitive Gamer from such a variant of MCTS, but we agree with the larger point that experiments and analyses of model variants can help to clarify how and to what

exent different components of the Intuitive Gamer model contribute to its better fit to human data. The Reviewer also makes a similar point later, under point 4 of their section "Clarify and Questions for the Authors". In fact our paper already contains several of these experiments, but we understand the reviewer did not appreciate that and we take responsibility for the misunderstanding! We explain below (in the paragraph beginning, "As noted in our response to the Reviewer's point 3…") how our experiments do indeed identify the contributions of the different parts of the Intuitive Gamer model, and how we have revised the paper to make these results more salient.

4 . "Explain practical computation details for the Reasoning Module. Provide explicit pseudocode or complexity analysis"

We agree; it is an excellent suggestion to add this pseudocode. We regret that the original submission did not include the Reasoning Module pseudocode adjacent to the Player Module pseudocode. We have now added pseudocode for the Reasoning Module to the Supplementary Information (see SI 2.1 "Model pseudocode", pg. 40).

On complexity analyses, we point the Reviewer to the Methods section entitled "Intuitive Gamer reasoning module" and the first sections of "Analysis methods" where we described the lower-level details of the Reasoning module (which may have led to some confusion, per your questions below). We realize our description might have been confusing and have tried to clarify the text to remove any potential confusion.

We also point out that Figure 3c and Extended Data Figure 1 are effectively complexity analyses on the number of samples (see below), as is the Extended Data Table 2 depicts efficiency across several of the algorithms considered. We now refer to these as such in the Methods and figure captions.

"how many rollouts are used"

As described in the caption of Figure 3 (pg. 6), the primary results are with k=6 simulated rollouts. This empirically best fits the variance in the human data. We describe how we estimated this in the section "Sample complexity analyses" of the Methods (pg. 25). To make these details easier to find in the paper, we have added clearer signposting towards the methods in the captions of Figure 2 and Figure 3, and the description in the Results. Hopefully the description of the reasoning module is also clearer now in the substantially tightened model description of the main text.

"how rollout length and sample size affect the estimates"

Figure 3c and Extended Data Figure 1 show how fit to the bootstrapped human data is impacted by a varied number of rollouts. This effectively let us estimate, coarsely, the number of samples.

As our main model runs game simulations to the end (see below), the rollout length extends to whenever a player either reaches the end condition (for win, or in misere games, loss), or the board fills up and the game is called a draw. Based on your questions, we have added a plot to the Supplementary Information showing the average length of rollouts across games (Supp Figure 20).

We recognize that the assumption of running full game simulations to the end is a strong one, and probably too simplistic (as we acknowledge in the Discussion, pg. 13). In follow-up work, we are actively exploring early-stopping game simulations, as we also discuss for the point below.

“what it means "the simulation ... may terminate early with some probability distribution on stopping times"”

This is a good question, and we regret our original text was not as clear as it should have been. We apologize for the confusion.

The Intuitive Gamer reasoning module can either estimate the expected outcomes, or other queries (e.g., estimated match length), based on running simulations of a match all the way to the end of the game. That is, as described above, the model simulates players selecting moves until either a player meets the win condition (see below) or the entire board fills up (rendering the match a draw). This is the way the reasoning module works for the main analyses reported in the paper.

Alternatively, however, the reasoning module could halt a simulation early and guess the end. That is, the outcomes of the game could be estimated from only running partial simulations. In exploratory analyses, we also studied such a model (see Methods “Partial game simulations.” and Extended Data Figure 2). However, there are several open design choices that warrant further study: if people are engaging in partial simulations of play, when do they stop simulating and how do they estimate the end of the game in those cases? We made relatively simple choices in this initial exploration: each partial simulation chooses a turn number at which to stop, sampled uniformly over the maximum possible moves based on board size; and a game that ends early is called a draw.

This is what we meant when we wrote that simulations “may terminate early with some probability distribution on stopping times”. We are actively exploring alternatives to these

settings in ongoing follow-up work, including halting probabilistically based on player progress. Based on your comments, we have changed the text to make clearer that the stopping could be probabilistic (but need not be based just on the time in the game).

“how "Computations over the simulated gameplay traces then inform the resulting distribution over the query values" (how to inform?). This will help readers evaluate the claim that the module "can evaluate games without ever playing" while remaining efficient.”

The predictions for the model when estimating the payoff of a game are computed via simulating matches, recording their outcomes (see above), and estimating the payoff based on those outcomes. We vary the number of simulations used to compute outcomes (see above, with respect to choosing k). These are further described in the “Analysis methods” section of the Methods, specifically “Computing and comparing payoff predictions” and “Sensitivity analyses into the number of samples k in the reasoning module.”

We realize our Main Text description of the model was potentially confusing; we have substantially overhauled and tightened our section “The Intuitive Gamer” in the Main Text.

Similar computations over simulations (e.g., assessing game length, and win rate over Random agent simulations) are composed to estimate the funness of a game. See “Funness model” description in Methods (pg. 22-23), and the description in “Is the game fun?” in the Main Text (pg. 7-8) which we have also revised in light of the Reviewers’ comments.

5. Report participant novelty controls and provide more participant detail.
Clarify how "novice" status was enforced or assessed in the Prolific study (screening questions, self-reports, prior experience checks, or exclusion criteria). If such controls were not used, report participants' prior exposure to Tic-Tac-Toe and the variants so readers can judge whether the cohort was truly novice.

We asked participants in the post-survey to self-report how much prior experience they had with several related games (Tic-Tac-Toe, Connect-4, and Gomoku) at the end of the “just think” study. We have added a figure (S.I. Figure 21, pg. 51) showing participant experience statistics to the Supplement. As expected, most participants had experience with Tic-Tac-Toe, fewer with Connect-4, and far less with Gomoku / Five in a Row. We can reasonably infer that most participants are novice for this genre of novel games, because a non-novice would certainly have much experience with Gomoku given its popularity in this genre. We are excited about future work that more properly explores the impact of experience on judgements (see response to Reviewer 1 and revised Discussion pg. 13).

Clarity and questions for the authors

1. Self-play in asymmetric games. How is self-play implemented for instances where player roles differ (different dynamics or winning rules for player 1 vs player 2)? Are both roles simulated and aggregated, or is a single-role simulation used?

Self-play here means that each player is the same player module (e.g., both play like Intuitive Gamers). As the Intuitive Gamer is goal-directed, it can "go after" any goal that it is given (see Equation 1, pg. 20). This means that if Player 1 and Player 2 have different win conditions, they can each play towards their corresponding goal, and we can imagine that a reasoner, imagining play in the game before any play, could imagine two players in these different roles each making decisions based on a few fast-and-flat simulations towards their goal. We have added details to the Methods on pg. 22 to make this clearer.

2. Center definition for U_aux. How is the board center computed on non square boards (e.g., 4×6) and on variant board topologies? Was any normalization applied across board sizes when using Euclidean distance?

Good question. We apologize for not being sufficiently specific in our description of this feature. Based on your question, we have clarified the description of the heuristics in Methods section "Intuitive Gamer player module" on pg. 21. We used Euclidean distance for center computations. On a 4x6 board, intuitively the "middle point" is the center, and the middle four cells are all closest to the center (<2, 3>, <2, 4>, <3, 3>, <3, 4>). This measure generalizes to arbitrary rectangular boards. We did apply normalization—on each board, distance is normalized by the distance between the center and the furthest corner.

3. Equation (3) correction. Equation (3) appears to be missing a term on its first right-hand side. Please confirm the intended formulation and update the manuscript.

Thank you for catching this! This was a typo (missing a term). We have updated the equation accordingly (pg. 2); note, the equation is now numbered Equation 2.

4. Please clarify the necessity of a separate Intuitive Gamer model (since it's a combination of value functions and tree search). Could a shallow, lightly-tuned MCTS with a Boltzmann action policy and simple handcrafted heuristics replicate the Intuitive Gamer's behaviour? If not, present evidence (or an argument) showing which element in the Intuitive Gamer's architecture is necessary to capture the behavioural signatures observed (i.e., the fundamental difference in the model)

As noted in our response to the Reviewer's point 3 of "Suggested improvements," we think that the current set of experiments in our paper varying model components does in fact show the necessity of all the different aspects of the Intuitive Gamer model, but we regret this was not clearly communicated and have tried to clarify here (as well as in the manuscript revision).

In hindsight we realize that the results of many experiments varying model components were distributed among several sections of our paper and thus not so easy for readers to appreciate. We have now put a full summary of all these experiments in Supplemental Information, with a brief pointer in the Main Text (on pg. 7) when we present the first set of variation experiments and overview in Methods section "Variations on the full Intuitive Gamer model" (pg. 24).

To recap this summary here, across multiple experiments varying different components of the Intuitive Gamer model we establish four claims about the model as an account of human reasoning, based on which model variations best fit human data:

- That simulations are flat: by "flat," we mean the Intuitive Gamer player module is depth-limited. Our comparisons to the Expert Gamer (which is approximately depth-5) in Figure 3a, as well as our comparisons to a more intermediate depth gameplayer (depth-3) in Fig 3b and SI Figure 50 show distinctly lower R^2 relative to human payoff predictions. We also find that computing the funness model features under a deeper gameplay agent (Extended Data Figure 4) does not capture human funness judgements as well as a depth-1 Intuitive Gamer agent. Similarly, we find that a depth-limited player module better captures people's prediction actions in-game and their judgements about others' likely play (see Figure 5, comparison between Intuitive Gamer and Expert Gamer).

- That simulations are fast: by "fast", we mean that the Intuitive Gamer's reasoning is fast. This is in part because simulations are shallow, of course, but also because only a small number of simulations need to be run to make each judgment at the level of resolution or confidence we see in people's judgments. The model's judgments are always stochastic, and by decreasing or increasing the number of simulations that each run of the reasoning module conducts per trial, we can obtain more or less variable judgments about game fairness and payoff over different runs of the model. By comparing the variance in judgments across Intuitive Gamer model runs (each run simulating a single human participant) with the variance in judgments across a sample of actual human participants, and equating for number of actual and simulated participants, we can estimate the effective number of simulations people are running (to the extent they are well modeled by an Intuitive Gamer model). We find that using only around 5-7 simulations from the Intuitive Gamer reasoning module best captures the variability in human judgements, and show this result in Figure 3c and Extended Data Figure 1 (the reasoning module is run with 6 simulations for the experiments in the text).

- That simulations are goal-directed: by “goal-directed,” we mean that the Intuitive Gamer player module chooses actions in a motivated fashion, towards its own goal—and assumes the other player is also motivated to meet their goal (and hence, the Intuitive Gamer tries to block the opponent’s progress). Ablating either or both of these two features—progress towards one’s own goal—is necessary to capture people’s payoff judgements (see Figure 3b), as well as their actual in-game moves and suggested moves for others (Extended Data Figure 7). Goal-directedness is also critical for capturing people’s decisions about whether or not to accept a draw request (Extended Data Figure 8).

- That simulations are probabilistic: by “probabilistic,” we mean that the Intuitive Gamer follows Boltzmann rationality, sampling actions from a softmax function over their estimated value (based on the above goal-directed heuristics). We also consider deterministic action selection (taking the estimated best action on each move, equivalent to a softmax at temperature zero), which we find neither qualitatively nor quantitatively captures the variance in people’s payoff predictions (see Figure 3a and 3b). We conduct a wider range of temperature analyses in the Supplementary Information.

We hope that with this summary, it is now clear why we think we have conducted the key experiments and analyses “showing which element in the Intuitive Gamer's architecture is necessary to capture the behavioural signatures observed (i.e., the fundamental difference in the model).”

Referee #2 (Remarks on code availability):

The provided code package includes a comprehensive README file containing installation instructions, directory structure descriptions, and information on the human participants’ data. A requirements.txt file is also included, clearly specifying all necessary Python modules and their corresponding versions. Most of the experimental results are stored in CSV and JSON formats. (Note: I did not install or execute the code myself.)

There are, however, a few minor issues worth noting:

* The directory related to "funness" (Reasoning Module) appears to be incomplete: it contains only an __init__.py file without any implementation scripts.

* The codebase includes an llm component that appears to provide LLM-based baselines and analyses related to game funness. However, these elements are not described or discussed in either the main paper or the appendix, leaving their purpose and relevance unclear.

Thank you for your close attention to detail on our code!

We apologize for the confusion. We had our funness scripts in the analysis/ folder. We have restructured our code, with clearer documentation.

And we do have several LLM baselines as alternate models in the Supplementary Information (see Supplement Figures 25 and 26). We also make reference to these in the Main Text (pg. in the “Alternate Models” subsection (pg. 5). These are exploratory analyses that are not core to the story of this paper, however, and warrant a more expansive paper in their own right!

Reviewer 3

We sincerely thank the Reviewer for their comments. We are grateful to hear your positive assessment of our work, given your cognitive science background (which we view as the primary field for our work, albeit with strong implications for AI and other disciplines, like game theory).

“My main concern with the manuscript in its present form is that the model is pitched as a model of games (the authors do acknowledge that they hope there will be followup work to extend it to other types of games). My worry is that as implemented the model is *too* specific to tic-tac-toe style games. Is there any reason to think the present model is the right one for board games more generally? For card games? For physical games? The reason it matters is that while the difference between a beginner and an expert in a tic-tac-toe style game may be well captured by depth of search, in many games experts may be using completely different sources of information compared to beginners. For example, a beginning poker player may not even be considering the social dimension of the game. A beginning tennis player may be focused on just returning the ball and is just unaware of most of the factors that characterize expert play.

The concrete request is to acknowledge the limitations more explicitly. What types of games do you think this model applies to?”

We take these points to heart. We agree that the paper is much better off acknowledging the limitations of the current model more explicitly. In response, we have made changes to both the Introduction and Discussion. In the Introduction, we now more clearly mark the limited class of games that this first paper treats ("a subclass of strategic grid-based board games", pg. 2). In the Discussion (pg. 13), we have rewritten the paragraph that highlights the challenge of extending and generalizing our model to other classes of games, both to better bring out the limitations of our current work and to present potential directions for addressing them. This paragraph now reads as follows:

"As a model of human reasoning, our work at present is limited to a subset of perfect information, two-player competitive board games. It is an open question how the heuristics our model uses could generalize to more complex games in the same class, such as Go or Chess. Many extensions are possible. For instance, the contiguous-line features we consider could be generalized to more general configural features, or connectivity or territory metrics that reflect partial or graded progress towards a game's win conditions. Natural language could be used to suggest short programs expressing such heuristics, as in verifiable code-based rewards in reinforcement learning (Lehrach et al, 2025). More broadly, we hope that our work will motivate studies of how people think about new games and novel problems well beyond those we have studied here, towards a general model of people's "intuitive theory of games" analogous to the computational models of intuitive theories of physics or intuitive theories of mind that cognitive scientists have constructed (Battaglia et al, 2013; Baker et al, 2017; Ullman et al, 2017). Such a theory requires testing on a wider class of games beyond competitive settings, e.g., towards cooperative or multi-agent contexts. While we hypothesize that key features of the Intuitive Gamer reasoning module---being fast, with a few probabilistic simulations of play, where play is assumed to involve goal-directed agents---would extend to other game classes, it is an open question how "flat" reasoning might be and what more general heuristics might capture intuitive reasoning in the wider range of games that characterize human social life."

Recognizing both the limitations of our work so far and some of the promising directions for extending it, we do hope that our paper inspires others in cognitive science as well as AI, game theory, and other fields, to try generalizing the Intuitive Gamer or analogous models to the vast diversity of games people can think about—and can create.

"In my judgment, the present visualizations are too focused on showing model fits, and do not effectively convey the patterns in the human data. Which games are judged to be most and least fun? Which factors are most predictive? (ditto for some of the other factors like likelihood of a draw and the advantage of going first)."

We greatly appreciate the encouragement to convey more of the structure in our human data, and in response we have added both more illustrations and qualitative results of what we found makes a fun game, as well as richer analyses and discussion of our quantitative results.

Based on the Reviewer's feedback, we wrote a new opening to the funness section of the main text (pg. 7) framed around patterns in the human data. We now show 12 representative games across a spectrum of the least fun to most fun human-rated games in Figure 4a and include a full listing of all 121 games, along with the average human funness rating, payoff prediction, draw likelihood, and first player advantage judgements (see S.I. Section 7).

We also refocused our presentation of quantitative results to better highlight what factors are most predictive. We now include a clearer statement of how all three components of our funness model contribute with different degrees of importance to predicting human judgments, in changes to the Main Text (pg 7-8), and the Methods sections "Funness model" (pg. 22-23) and "Fitting regression models to funness judgments" (pg. 25). Based on the Reviewer's feedback, we also created a new Extended Data figure (Extended Data Figure 4, pg. 35) which specifically shows the importance of each feature contribution and compares it to alternate models. (Previously, the feature contributions were in the SI and the alternate model comparison was a panel in the main text figure.)

We contrast these results with a quantitative analysis of linear models fit to superficial game features (e.g., board size, number of pieces in a row needed to win) and show these factors do not capture much variance in human fun judgements. This analysis was present in our previous submission, but is now referred to more explicitly in the Main Text (p. 8) and detailed in the Methods section "Assessing contribution of non-simulation-based features to funness judgments" and Extended Data Figure 4. (More granular analysis of individual non-simulation-based feature importances are also in SI Figure 30.)

Finally, to further illustrate patterns in the human data and how our model explains them, we have introduced a new Extended Data Figure 3 which shows quantitatively how human funness (as well as game payoff) judgments varies with K, the number of pieces needed to connect for a win, on 10x10 game boards. We choose this subset as it nicely highlights interesting structure in the human data (e.g., funness peaking at a moderate level K; estimated payoff plateauing towards zero with increased K). The figure also shows how the Intuitive Gamer model captures this trend quantitatively.

Taken together, these results show that what makes people think a game is likely to be fun appears to be not a simple or superficial function of the game's rules, but rather an estimate of its deeper cognitive virtues: whether the game seems like it would lead to a fair competition with a decisive win or loss, and whether it would stimulate productive thinking—for a novice player thinking not too far ahead with reasonable heuristics to guide their thinking. We hope that the revisions described above now much better convey this picture, along with the ability of our Intuitive Gamer approach to express these insights in a principled and quantitative computational model.

"Please add color legends to the figures where applicable so that the reader doesn't have to read through the whole caption or refer to other figures to understand the color mapping."

Thank you for pointing this out! We have added missing color legends to Figure 5b as well as Extended Data Figure 6.

“Figure 1 caption: “how many pieces a any player can make” (can make?)”

Thank you for catching this typo! We have fixed this phrase.

Dear Dr. Sweeney,

We thank you and Reviewer 2 for your continued time and engagement!

We have revised our manuscript to account for Reviewer 2's additional comments and include a point-by-point response below. Reviewer text is in black, our responses are in green; we indicate new text we added in ***bold and italics*****.** We have also changed the title to your editorial request. We are happy to make further revisions and adjust file format for any figures/tables, etc, if needed.

We also plan to submit cover art suggestions within the next two weeks.

Reviewer 2

We thank the Reviewer for their continued engagement with our manuscript. We are glad that most of our changes satisfied the Reviewer's questions and previous concerns. We made further amendments to address your remaining concerns.

2. Positioning Relative to MCTS
The added subsection comparing Intuitive Gamer and MCTS improves clarity. However, I remain partially unconvinced that the distinction is entirely conceptual rather than parametric. While the authors argue that "any stochastic tree search" could resemble MCTS at a high level, my original suggestion was more specific: to empirically compare against a deliberately constrained MCTS configured with the same heuristics and action policy. I think the paper may explicitly acknowledge that the boundary between "shallow MCTS" and "Intuitive Gamer" is partly architectural emphasis rather than algorithmic novelty and that the contribution lies in the cognitive interpretation and modular framing rather than proposing a fundamentally new search algorithm, i.e., intuitive gamer.

Thank you for clarifying your suggestion. We have adjusted the Methods subsection comparing MCTS and the Intuitive Gamer section (pg. 22) to acknowledge the boundary is partly a matter of emphasis that permits a clearer cognitive interpretation (our new changes are in **bold and italics**). The subsection now reads as follows: "We briefly clarify ***the relation*** between MCTS—the planning algorithm behind many AI systems that achieve expert-level gameplay such as AlphaGo and AlphaZero—and ***the*** contribution the Intuitive Gamer model offers as a model of human reasoning. ***One could potentially view the Intuitive Gamer's player module as a particularly restricted or shallow variant of MCTS (as MCTS is also compatible with constraining computational resources and adopting heuristic functions). However, for several reasons we think it is important to distinguish between these classes of models — recognizing***

that this is in part a matter of scientific emphasis and interpretation rather than algorithmic innovation.

First, framing MCTS so generally as to include the Intuitive Gamer's play would also include almost any kind of stochastic tree search, including other models in our comparison set, e.g., the Random Gamer. More deeply, it would leave out core features of MCTS that have made it so powerful in AI systems as well as the most distinctive features of the Intuitive Gamer as a cognitive model. MCTS made such an impact in AI gameplay and reinforcement learning (Coulom, 2006; Genesereth et al, 2014; Silver et al, 2016; Silver et al, 2017) precisely because it could achieve very strong play without the need for heuristics to guide search. Instead, it uses extensive inference-time computation and sophisticated tree traversal arithmetic (backtracking, node visit counting) to effectively explore a very large, unstructured game tree. This is in contrast to the Intuitive Gamer, which does not require any sophisticated algorithms and uses very minimal computation, although it does rely on a small number of abstract heuristics when assessing the value of next moves. Such a design leads the Intuitive Gamer to be a highly efficient if less than optimal player, and one which we believe much better captures how people reason (as evidenced in our behavioral action selection and action prediction studies). As described in the "Resource-rational reasoning" section of the Main Text and captured in Extended Data Table 2b), the Intuitive Gamer is orders of magnitude more efficient than either MCTS (run for 10,000 iterations, as described above) or the Expert Gamer, in terms of wallclock time and number of board states evaluated."

3. Utility Decomposition

The grounding of progress/blocking/cantering in prior literature and the sensitivity analyses clarify motivation. However, I do not find the exclusion of learning-based decompositions fully convincing. Even under a zero-experience framing, one could test features learned across related games (but not the specific instance) to assess whether handcrafted heuristics are uniquely necessary. Without such a comparison, it remains unclear whether the proposed decomposition reflects psychologically grounded priors or modelling convenience. This should be either better justified or explicitly acknowledged as a limitation.

We are glad our revisions to the heuristic description helped clarify motivation. We agree it is important to understand where the heuristics people employ come from, and we are actively exploring efficient heuristic learning and transfer in ongoing work. We have clarified that heuristic learning and transfer are current limitations of our model in the Discussion (pg. 9), in the following passage: ***"Our model also does not address how people may learn and adapt such heuristics within or across games, limitations that suggest important directions for future work."*** We have also added a note to the Methods subsection describing our modeling choices around the heuristics (pg. 19): "***Future work should explore other modeling choices to capture***

how people might represent and combine multiple utility functions, including the role of learning in how people might come to flexibly synthesize these functions."

5. Novice Status

Supplemented participant experience statistics are helpful. However, post-survey self-reports of prior experience may not be very rigorous and can be vulnerable to bias. If the novice framing is central, stronger controls (e.g., pre-screening or exclusion criteria) are needed. Otherwise, I recommend careful phrasing: this reflects novelty within a familiar genre, not complete inexperience.

We have added clarification in the Discussion (pg. 8) that "***these games reflect novelty within a genre that many people are familiar with rather than complete novel experience.***" This aligns with the clarification we added to the Introduction (pg. 2) in our previous revision that we explore "games from a subclass of strategic grid-based board games that are not entirely unlike games they have likely seen before, e.g., Tic-Tac-Toe or Connect-4, but vary in their rules and underlying dynamics."
